# Nonenzymatic synthesis of anomerically pure, mannosyl-based molecular probes for scramblase identification studies

**DOI:** 10.3762/bjoc.16.145

**Published:** 2020-07-20

**Authors:** Giovanni Picca, Markus Probst, Simon M Langenegger, Oleg Khorev, Peter Bütikofer, Anant K Menon, Robert Häner

**Affiliations:** 1 Department of Chemistry and Biochemistry, University of Bern, Freiestrasse 3, 3012 Bern, Switzerland; 2 Institute of Biochemistry and Molecular Medicine, University of Bern, Bühlstrasse 28, 3012 Bern, Switzerland; 3 Department of Biochemistry, Weill Cornell Medical College, 1300 York Avenue, 10065 New York, United States of America

**Keywords:** carbohydrates, citronellol, phosphoramidite, photoclickable glycolipid analogs, scramblase

## Abstract

The chemical synthesis of molecular probes to identify and study membrane proteins involved in the biological pathway of protein glycosylation is described. Two short-chain glycolipid analogs that mimic the naturally occurring substrate mannosyl phosphoryl dolichol exhibit either photoreactive and clickable properties or allow the use of a fluorescence readout. Both probes consist of a hydrophilic mannose headgroup that is linked to a citronellol derivative via a phosphodiester bridge. Moreover, a novel phosphoramidite chemistry-based method offers a straightforward approach for the non-enzymatic incorporation of the saccharide moiety in an anomerically pure form.

## Introduction

Mannosyl phosphoryl dolichol (MPD), an important, multifunctional glycolipid, is used as a mannose donor for protein N-glycosylation, O- and C-mannosylation, and glycosylphosphatidylinositol (GPI) anchoring in the luminal leaflet of the endoplasmic reticulum (ER) [1–8]. Interestingly, MPD is synthesized on the cytoplasmic face of the ER and must be translocated across the ER membrane to participate in luminal glycosyltransfer reactions [3–4]. A specific membrane protein – MPD scramblase – is required to facilitate the transbilayer movement of MPD across the ER. Although the activity of MPD scramblase has been described in microsomal vesicles and reconstituted systems [1–2,9], the molecular identity of this protein remains unknown. To circumvent the need for traditional purification strategies to identify the scramblase, we considered the use of photoreactive, clickable MPD mimics. As such, an attempt already showed great promise in a previous report by Rush et al. from 2015 [10]. Similarly, we envisioned that these analogs could be used to capture MPD-recognizing proteins, including the scramblase, from a crude mixture of ER membrane proteins [11–12]. The captured proteins would be subsequently identified by mass spectrometry, and their function in MPD scrambling validated by biochemical and genetic approaches.

**Figure 1:**
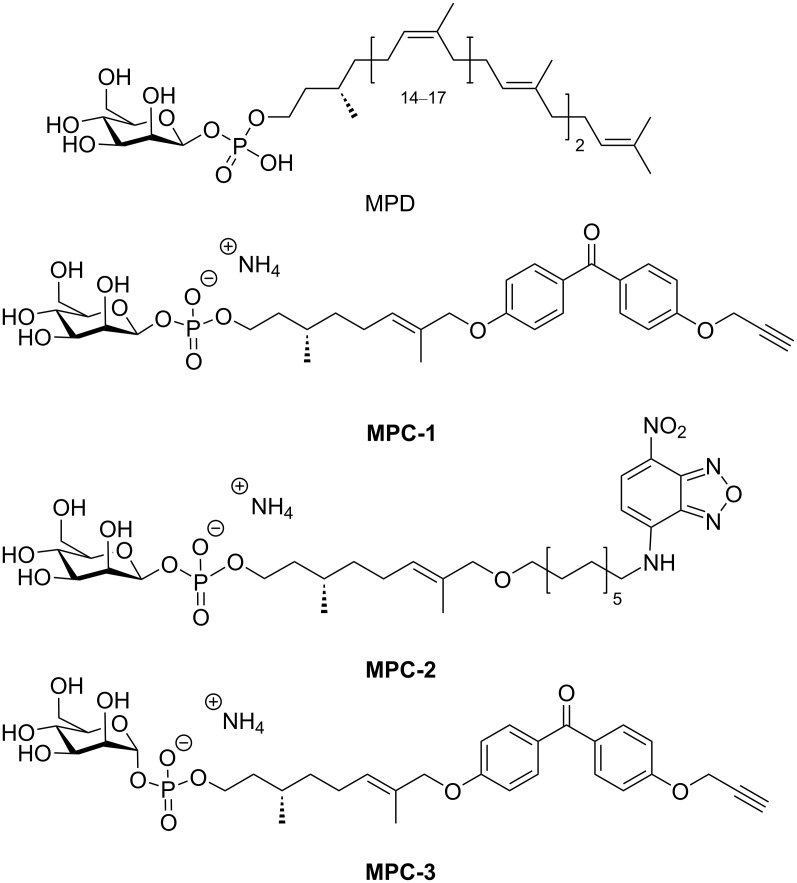
Chemical structures of MPD and the three structural analogs **MPC-1**, **MPC-2**, and **MPC-3**. The molecular probes **MPC-1** and **MPC-3** are photoclickable derivatives, whereas the probe **MPC-2** bears a fluorescent tag.

A suitable molecular probe and mimic of MPD (Figure 1) can be subdivided into three essential components: a β-ᴅ-mannose (the α-anomer also shows biological activity, but to a lesser extent) [13–15], a short-chain (citronellol) mimic of dolichol [1–2,13], and a functional tag. The latter may either be a chemically reactive group (in **MPC-1**) or a fluorescent reporter group (in **MPC-2**). **MPC-1** bears a benzophenone moiety for photocrosslinking to MPD-recognizing proteins. The presence of an additional propargyl group provides a way to further derivatize the probe with biotin azide via click-type chemistry [16] for the isolation of protein–lipid adducts using streptavidin resins [10]. To synthesize such molecular probes, previous studies relied on a chemoenzymatic approach to selectively incorporate the biologically relevant β-configured ᴅ-mannose headgroup as chemical approaches were deemed to be too challenging [10,13]. This method naturally has some limitations regarding the quantity of the obtained compound and the availability of the required mannosyltransferase. Here, we describe a purely chemical approach to synthesize a photoclickable MPD analog containing β-ᴅ-mannose (in **MPC-1**). The chemical synthesis allows for a rapid upscaling of the reactions if necessary. In addition, having access to the individual building blocks (e.g., the phosphoramidites) will enable the creation of a series of molecular probes with different functional groups and varying linker lengths with relatively little effort.

The crucial step in the synthetic pathway consists of the conversion of the carbohydrate intermediates into stereodefined, anomeric phosphoramidite derivatives. The method is based on adapted procedures developed for DNA solid-phase synthesis [17]. Phosphoramidite chemistry allows the connection of two molecular entities via a phosphodiester linkage and was found to be perfectly suited for this purpose. Reports of using phosphoramidite chemistry for the preparation of carbohydrates via the anomeric position are relatively rare [18–23]. Alternatively, the H-phosphonate approach has been used to convert carbohydrates into phosphate-linked derivatives at the anomeric center [24–29].

The phosphoramidite approach was also applied to the synthesis of a second molecular probe, **MPC-2**, an anomerically pure, β-linked ᴅ-mannosephosphate derivative, which serves as a fluorescent MPD analog for scramblase activity screening assays. Although similar experiments have been described with radioactively labeled substrates, the use of fluorescently labeled probes offers several advantages, including the continuous monitoring of the transport and a better time resolution. For reasons of comparison, the α-configured ᴅ-mannose probe **MPC-3** was synthesized in parallel. The characterization of the configuration at the anomeric position was done by HSQC NMR (see Supporting Information File 1). Since the target enzymes are unknown and can be expected to have stereospecific binding sites, the α-configured ᴅ-mannose probe **MPC-3** is also important as a reference for our biochemical assays (work in progress). The possibility to use both probes, **MPC-1** and **MPC-3**, independently may help to reduce false-positive scramblase candidates.

## Results and Discussion

**Figure 2:**
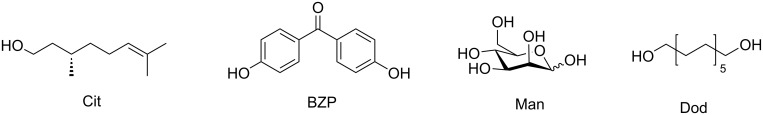
Chemical structures of commercially available (*S*)-citronellol (Cit), 4,4′-dihydroxybenzophenone (BZP), ᴅ-mannose (Man), and 1,12-dodecanediol (Dod), the main starting materials in the synthesis of **MPC-1** and **MPC-2**.

The synthesis of the target compounds **MPC-1** and **MPC-2** started from commercially available (*S*)-citronellol (Cit), 4,4′-dihydroxybenzophenone (BZP), D-mannose (Man), and 1,12-dodecanediol (Dod, Figure 2).

**Figure 3:**
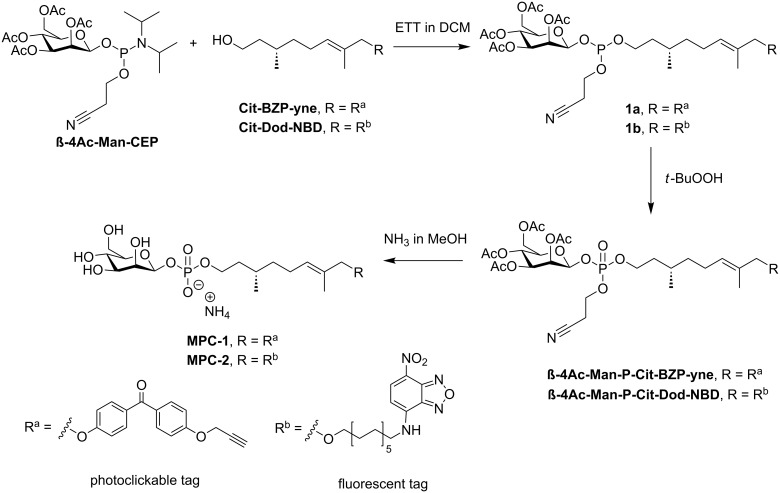
The synthetic route leading to compounds **MPC-1** and **MPC-2**. Compound **β-4Ac-Man-CEP** was prepared in 4 steps from ᴅ-mannose (see Supporting Information File 1) [35–36]. Compound **Cit-BZP-yne** was prepared via a Mitsunobu reaction of tetrahydropyranyl (THP)-protected citronellol and a hydroxylated benzophenone derivative [10,37–38]. Compound **Cit-Dod-NBD** was obtained in 10 steps, starting from citronellol and *tert*-butyldimethylsilyl (TBDMS)-protected dodecanediol [39]. The reaction of either of the alcohols with the sugar phosphoramidite using ETT led to phosphite intermediates **1a** and **1b**, respectively. The intermediates were then oxidized with *t*-BuOOH, and finally, the protecting groups were removed under basic conditions to give either **MPC-1** or **MPC-2** as ammonium salts. ETT = 5-(ethylthio)-1*H*-tetrazole.

Both the mannose and the phosphodiester bond were introduced via phosphoramidite chemistry to yield the final compounds, as shown in Figure 3. As opposed to the photoclickable probe **MPC-1**, the 4-chloro-7-nitro-1,2,3-benzoxadiazole (NBD)-labeled analog **MPC-2** carries an additional dodecanyl linker between the citronellyl unit and the fluorophore. This is intended to increase the hydrophobic interactions between the probe and the lipid bilayer and also to increase the flexibility. More specifically, the probe should adopt a U-shaped structure in which the citronellyl–dodecanyl linker is located in the phospholipid membrane, and the mannose and the NBD are positioned in the surrounding aqueous medium. The close proximity of the NBD fluorophore to the membrane–water interface [30–31] allows for fluorescence-based scramblase assays wherein the NBD can be detected with dithionite. The latter reduces the nitro group of the NBD rapidly into an amino group, rendering the fluorophore nonfluorescent [32–34]. While there are no noteworthy difficulties in the synthesis of the molecular probe **MPC-1** to report, the same cannot be said for **MPC-2**. There, the NBD tag was only partially stable to the conditions used to remove the protecting groups in the final step (NH_3_ in MeOH, Figure 3). Under such alkaline conditions, a considerable amount of degradation of NBD was observed, making an additional purification step necessary to obtain pure **MPC-2** (see Supporting Information File 1).

As shown in Figure 3, the acetylated mannose phosphoramidite **β-4Ac-Man-CEP** was coupled to the free hydroxy group of the citronellol derivatives containing the functional tags (**Cit-BZP-yne** or **Cit-Dod-NBD**). The reactions were performed in dichloromethane (DCM), and ETT served as the activator. The subsequent oxidation of the unstable phosphite triester to the more stable phosphotriester was performed with *t*-BuOOH. The resulting intermediates were then treated overnight with a solution of ammonia in methanol to remove the protecting groups. An overall yield of about 45% was achieved when no further purification was carried out (for details see Supporting Information File 1).

The major goal of this work was the synthesis of molecular probes with a configurationally defined mannosyl headgroup, i.e., a pure β-anomer. In order to verify the preferred configuration at the anomeric position and to prove the working principle of our synthetic strategy, we used 2D NMR experiments. Coupled HSQC measurements revealed the ^1^*J*_CH_ coupling constants of our compounds and thereby the absolute configuration at the anomeric center of the carbohydrate [40–42]. For **MPC-1** and **MPC-2**, ^1^*J*_CH_ values 158 Hz and 161 Hz, respectively, were obtained (for comparison, for **MPC-3** containing almost exclusively the α-anomer, a value of about 171 Hz was determined, see Supporting Information File 1).

**Figure 4:**
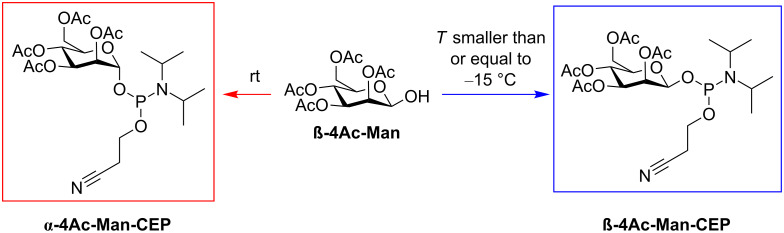
Preparation of mannosyl phosphoramidites. Starting from 2,3,4,6-tetra-*O*-acetyl-β-ᴅ-mannopyranose (**β-4Ac-Man**), the phosphitylation using 2-cyanoethyl *N*,*N*-diisopropylchlorophosphoramidite (CEP-Cl) and DIPEA provides **β-4Ac-Man-CEP** when the reaction is performed at a temperature below −15 °C (15–30 min reaction time). When the reaction is carried out at room temperature, epimerization leads to 2,3,4,6-tetra-*O*-acetyl-α-ᴅ-mannopyranose (not shown), which is subsequently converted to **α-4Ac-Man-CEP**.

As highlighted above, the most important step in the chemical synthesis of the target compounds **MPC-1** and **MPC-2** was the preparation of the phosphoramidite **β-4Ac-Man-CEP** (Figure 4). Two points are of particular importance for the synthesis of this compound. Firstly, we needed access to an anomerically pure starting material, i.e., 2,3,4,6-tetra-*O*-acetyl-β-ᴅ-mannopyranose. This was achieved by following literature procedures [35–36], which provided the pure β-anomer as a crystalline solid. Secondly, suitable conditions had to be identified for the subsequent phosphitylation conditions under which no mutarotation of the carbohydrate occurs. Cooling of the reaction mixture and performing the reaction at a temperature below −15 °C yielded the compound **β-4Ac-Man-CEP**, with no traces of the α-anomer. The configuration was then retained throughout the rest of the synthesis, as indicated by the NMR data of **MPC-1** and **MPC-2** (Supporting Information File 1).

## Conclusion

We report herein the successful chemical synthesis of molecular probes for identifying and studying MPD scramblase, as well as proteins that use MPD as a mannosyl donor. Two types of probes were synthesized; both are short-chain glycolipid analogs. One, **MPC-1**, contains a photoreactive clickable tag to capture scramblase candidates (identification by mass spectrometry), and the other probe, **MPC-2**, consists of a fluorescent label to test candidates for scramblase activity in reconstitution-based assays. The molecular probes were prepared via phosphoramidite chemistry, which allowed the incorporation of the carbohydrate headgroup and simultaneously introduced the linking phosphate group. Furthermore, we demonstrate a novel, reliable, and efficient way to synthesize the carbohydrate phosphoramidite with a defined configuration at the anomeric position, a strategy that gives access to future molecular probes containing pure β-ᴅ-mannose, which was previously only accessible through a chemoenzymatic approach.

## Experimental

### Synthesis

Detailed descriptions of the synthesis and the analytical data (NMR and MS spectra) are provided in Supporting Information File 1.

#### Cit-BZP-yne

**THP-Cit-BZP-yne** (279 mg, 0.57 mmol, 1 equiv) was dissolved in anhydrous ethanol (8 mL), and pyridinium *p*-toluenesulfonate (PPTS, 286 mg, 1.14 mmol, 2 equiv) was added. The solution was stirred at 60 °C for 2.5 h. The reaction mixture (at rt) was poured into a separating funnel containing diethyl ether (40 mL) and brine (16 mL). The organic layer was dried over MgSO_4_, and the volatile components were removed under reduced pressure. The yellowish residue (crude product) was purified by flash chromatography over silica gel, with a mixture of *n*-hexane/ethyl acetate 3:2, v/v as eluent. The fractions containing the product (*R*_f_ = 0.22 in *n*-hexane/ethyl acetate 3:2, v/v) were combined, and the solvents were removed under reduced pressure on a rotary evaporator to give **Cit-BZP-yne** (154 mg, 0.38 mmol, 67%) as white solid. ^1^H NMR (300 MHz, CDCl_3_, δ):7.85–7.72 (m, 4H), 7.09–6.99 (m, 2H), 7.02–6.91 (m, 2H), 5.61–5.49 (m, 1H), 4.77 (d, *J* = 2.4 Hz, 2H), 4.47 (s, 2H), 3.69 (m, 2H), 2.56 (t, *J* = 2.4 Hz, 1H), 2.21–1.98 (m, 2H), 1.74 (s, 3H), 1.70–1.50 (m, 1H), 1.50–1.15 (m, 4H), 0.92 (d, *J* = 6.4 Hz, 3H); ^13^C NMR (75 MHz, CDCl_3_, δ) 194.48, 162.39, 160.64, 132.22, 132.14, 131.64, 130.51, 130.29, 129.82, 114.37, 114.36, 77.92, 76.10, 74.21, 61.12, 55.89, 39.83, 36.63, 29.18, 25.20, 19.46, 13.85.

#### β-4Ac-Man-CEP

**β-4Ac-Man** (100 mg, 0.29 mmol, 1 equiv) was dissolved in anhydrous DCM (4 mL) at −60 °C. Then, DIPEA (75 μL, 0.43 mmol, 1.5 equiv) was added, followed by the addition of CEP-Cl (77 μL, 0.35 mmol, 1.2 equiv). The mixture was stirred for 30 min at −50 °C under argon. The crude product was then directly purified by flash chromatography using ethyl acetate/*n*-hexane 1:1, v/v + 2% triethylamine. The fractions containing the product (*R*_f_ = 0.40) were combined, and the solvents were removed under reduced pressure on a rotary evaporator. The product was extensively dried to give **β-4Ac-Man-CEP** (145 mg, 0.26 mmol, 92%) as colorless foam. ^1^H NMR (300 MHz, CDCl_3_, δ) 5.37 (dd, *J* = 25.2, 3.3 Hz, 1H), 5.19 (t, *J* = 9.9 Hz, 1H), 5.12–4.95 (m, 2H), 4.24–4.09 (m, 2H), 3.91–3.48 (m, 5H), 2.66–2.53 (m, 2H), 2.14 (s, 3H), 2.05–2.00 (m, 8H), 1.95 (d, *J* = 1.6 Hz, 3H), 1.12 (dd, *J* = 10.8; 6.9 Hz, 12H); ^13^C NMR (75 MHz, CDCl_3_, δ): 170.58, 170.55, 170.31, 170.10, 169.98, 169.94, 169.67, 169.63, 117.63, 93.69, 93.57, 93.36, 72.72, 72.66, 70.98, 69.78, 69.69, 65.99, 65.90, 62.67, 62.43, 59.20, 58.96, 58.78, 58.53, 44.07, 43.90, 43.77, 43.59, 24.61, 24.52, 24.43, 24.34, 24.04, 23.94, 20.80, 20.77, 20.68, 20.56, 20.34, 20.25, 20.14, 20.06; ^31^P NMR (121 MHz, CDCl_3_, δ) 153.31, 150.00.

#### MPC-1

ETT (72 mg, 0.55 mmol, 1.5 equiv) was dissolved in anhydrous DCM (2 mL), **β-4Ac-Man-CEP** (223 mg, 0.41 mmol, 1.1 equiv) was added, and the resultant solution was added to a solution of **Cit-BZP-yne** (150 mg, 0.37 mmol, 1 equiv) dissolved in anhydrous DCM (2 mL). The reaction mixture was stirred for 30 min at rt under argon. Afterwards, a *t*-BuOOH solution (201 μL, 1.11 mmol, 3 equiv, ≈5.5 M in decane) was added, and the mixture was stirred for an additional 15 min. The reaction mixture was diluted with toluene (60 mL) and then washed with a saturated sodium hydrogen carbonate solution (30 mL) and brine (30 mL). The organic layer was dried over MgSO_4_, and the volatile components were removed under reduced pressure. The resulting residue was dissolved in methanol and purified by PLC (using ethyl acetate/*n*-hexane 2:1, v/v as a mobile phase). The broad band, located at the very bottom of the plate (*R*_f_ ≈ 0.10 using ethyl acetate/*n*-hexane 1:1, v/v) was removed, and the intermediate **β-4Ac-Man-P-Cit-BZP-yne** was extracted with methanol (150 mL). The solvent was removed under reduced pressure on a rotary evaporator. Then, an ammonia solution (3 mL, 2.0 M in methanol) was added, and the reaction mixture was stirred overnight at rt. The resulting solution was first diluted with methanol (2 mL), and the volatile components were removed under reduced pressure. Then, distilled water (2 mL) was added, and the material was lyophilized overnight to afford **MPC-1** (110 mg, 0.17 mmol, 46%) as yellowish solid. ^1^H NMR (400 MHz, DMSO-*d*_6_, δ) 7.75–7.66 (m, 4H), 7.17–7.04 (m, 4H), 5.58 (t, *J* = 6.7 Hz, 1H), 4.92 (d, *J* = 2.4 Hz, 2H), 4.87 (d, *J* = 8.5 Hz, 1H), 4.51 (s, 2H), 3.77–3.61 (m, 5H), 3.62–3.32 (m, 2H), 3.33–3.25 (m, 2H), 2.05 (m, 2H), 1.69 (s, 3H), 1.62–1.48 (m, 2H), 1.42–1.26 (m, 2H), 1.26–1.13 (m, 1H), 0.87 (d, *J* = 6.2 Hz, 3H); ^13^C NMR (101 MHz, DMSO-*d*_6_, δ) 193.64, 162.46, 160.86, 132.29, 132.13, 131.24, 130.63, 130.30, 129.66, 115.07, 115.02, 96.07, 96.04, 79.25, 79.20, 74.05, 73.98, 71.53, 71.48, 67.39, 63.02, 62.97, 61.82, 56.20, 37.85, 37.78, 36.76, 36.36, 29.33, 25.14, 19.69, 14.17; ^31^P NMR (121 MHz, DMSO-*d*_6_, δ) 25.54, 4.74, −2.89.

#### Cit-Dod-NBD

**DMT-Cit-Dod-NBD** (787 mg, 0.96 mmol) was dissolved in a trichloroacetic acid solution (3% in DCM/MeOH 1:1, v/v) at rt. The reaction mixture was stirred for 2 h. The mixture was diluted with DCM, washed with brine, and the organic phase was dried with Na_2_SO_4_. The purification by column chromatography on silica gel (hexane/EtOAc 6:4, v/v) gave **Cit-Dod-NBD** (455 mg, 92%) as red oil (*R*_f_ = 0.5 in hexane/EtOAc 1:1, v/v). ^1^H NMR (DMSO-*d*_6_, 300 MHz, δ) 9.55 (s, 1H), 8.50 (d, *J* = 8.8 Hz, 1H), 6.49–6.31 (m, 1H), 5.33 (t, *J* = 7.2 Hz, 1H), 4.28 (t, *J* = 5.1 Hz, 1H), 3.73 (s, 2H), 3.59–3.36 (m, 4H), 3.26 (t, *J* = 6.4 Hz, 2H), 1.74–1.61 (m, 2H), 1.55 (s, 3H), 1.52–1.40 (m, 4H), 1.24 (s, 19H), 0.84 (d, *J* = 6.5 Hz, 3H); ^13^C NMR (DMSO-*d*_6_, 75 MHz, δ) 132.41, 127.51, 99.47, 79.67, 76.21, 69.19, 63.78, 59.24, 37.01, 33.97, 30.68, 29.63, 29.47, 29.42, 29.18, 28.81, 28.06, 26.85, 26.19, 25.00, 24.92, 19.86, 19.09, 14.06, 13.99.

#### MPC-2

ETT (38 mg, 0.29 mmol, 1.5 equiv) was dissolved in anhydrous DCM (2 mL), **β-4Ac-Man-CEP** (116 mg, 0.21 mmol, 1.1 equiv) was added, and the resultant solution was added to **Cit-Dod-NBD** (100 mg, 0.19 mmol, 1 equiv) dissolved in anhydrous DCM (1 mL). The reaction mixture was stirred for 30 min at rt under argon. Afterwards, a *t*-BuOOH solution (110 μL, 0.61 mmol, 3 equiv, ≈5.5 M in decane) was added, and the mixture was stirred for an additional 15 min. The reaction mixture was poured into a separating funnel containing DCM (15 mL) and a saturated sodium hydrogen carbonate solution (15 mL). The organic layer was further washed with brine (15 mL) and then dried with MgSO_4_. The volatile components were removed under reduced pressure, the brownish residue was redissolved in acetonitrile (1.5 mL) and purified by PLC (using a mixture of ethyl acetate/*n*-hexane 1:1, v/v as the mobile phase). The broad band located at the very bottom of the plate (*R*_f_ ≈ 0.10 using ethyl acetate/*n*-hexane 1:1, v/v) was removed, and the intermediate **β-4Ac-Man-P-Cit-Dod-NBD** was extracted with acetonitrile (180 mL). The solvent was removed under reduced pressure on a rotary evaporator. The material was then treated overnight with an ammonia solution (2 mL, 2.0 M in methanol). Afterwards, the reaction mixture was diluted with methanol (2 mL), the volatile components were removed under reduced pressure, and the orange-brownish residue (redissolved in 1.5 mL methanol) was purified by PLC (using chloroform/methanol/water 60:25:4, v/v/v as the mobile phase). The broad band, centered at around an *R*_f_ of about 0.22, was removed, and the product was extracted with methanol (170 mL). The solvent was removed under reduced pressure on a rotary evaporator, the residue dissolved in distilled water (1.3 mL), and the material was lyophilized overnight to give **MPC-2** (65 mg, 0.09 mmol, 45%). ^1^H NMR (300 MHz, D_2_O, δ) 8.27 (s, 1H), 6.17 (s, 1H), 5.35 (s, 1H), 5.13 (d, *J* = 8.4 Hz, 1H), 4.14–3.53 (m, 10H), 3.50–3.19 (m, 4H), 2.05–1.99 (m, 2H), 1.78–1.42 (m, 9H), 1.33–1.08 (m, 21H), 0.91 (d, *J* = 6.0 Hz, 3H); ^13^C NMR (101 MHz, D_2_O, δ) 131.98, 131.89, 131.83, 128.40, 128.33, 95.42, 95.38, 76.90, 76.65, 72.73, 71.17, 71.12, 69.33, 69.32, 66.19, 64.79, 61.20, 60.82, 37.11, 29.76, 29.61, 29.52, 29.39, 29.23, 26.26, 25.09, 18.99, 13.57; ^31^P NMR (121 MHz, D_2_O, δ) 7.22, −1.64.

#### MPC-3

Compound **α-4Ac-Man-CEP** was prepared according to published procedures [22] (for more information see Supporting Information File 1).

**α-4Ac-Man-CEP** (252 mg, 0.46 mmol, 1.1 equiv) and ETT (82 mg, 0.63 mmol, 1.5 equiv) were dissolved in anhydrous DCM (1 mL) and **Cit-BZP-yne** (170 mg, 0.42 mmol, 1 equiv, dissolved in 1.5 mL anhydrous DCM) was added. The reaction mixture was stirred for 30 min at rt under argon. Afterwards, a *t*-BuOOH solution (182 μL, 1.31 mmol, 3 equiv, 70 wt % in H_2_O) was added, and the solution was stirred for an additional 15 min. The reaction mixture was diluted with toluene (15 mL) and poured into a separating funnel containing a saturated sodium hydrogen carbonate solution (10 mL). The organic phase was washed with brine, dried with MgSO_4_, and the volatile components were removed under reduced pressure. The resulting material was then dissolved in DCM and purified by flash chromatography on silica gel using ethyl acetate/*n*-hexane 7:3, v/v as eluent. The fractions containing the intermediate **α-4Ac-Man-P-Cit-BZP-yne** (*R*_f_ = 0.33 using the same solvent system as for the column) were combined, and the solvents were removed under reduced pressure on a rotary evaporator. To the white residue, an ammonia solution (4 mL, 2.0 M in methanol) was added, and the reaction mixture was stirred overnight at rt. Afterwards, the volatile components were removed under reduced pressure, the residue was dissolved in methanol (2 mL), and the material was purified by PLC (using chloroform/methanol/water 60:25:4, v/v/v as the mobile phase). The broad band, centered at around *R*_f_ ≈ 0.19, was removed, and the product was extracted with methanol (100 mL). The solvent was removed under reduced pressure on a rotary evaporator to give **MPC-3** (65 mg, 0.10 mmol, 24%) as white solid. ^1^H NMR (400 MHz, DMSO-*d*_6_, δ) 7.75–7.64 (m, 4H), 7.16–7.04 (m, 4H), 5.58 (t, *J* = 7.2 Hz, 1H), 5.17 (dd, *J* = 7.8, 1.9 Hz, 1H), 4.91 (d, *J* = 2.4 Hz, 2H), 4.88–4.87 (m, 3H), 4.76–4.71 (m, 2H), 4.50 (s, 2H), 3.77–3.63 (m, 2H), 3.63–3.51 (m, 4H), 3.42–3.23 (m, 2H), 2.04 (m, 2H), 1.69 (s, 3H), 1.60–1.45 (m, 2H), 1.41–1.24 (m, 2H), 1.24–1.10 (m, 1H), 0.85 (d, *J* = 6.4 Hz, 3H); ^13^C NMR (101 MHz, DMSO-*d*_6_, δ) 193.74, 162.47, 160.84, 132.31, 132.15, 131.22, 130.61, 130.26, 129.71, 115.07, 115.03, 96.05, 95.99, 79.24, 79.14, 74.51, 74.06, 71.46, 71.38, 70.92, 67.64, 62.81, 62.76, 61.80, 56.17, 37.90, 37.83, 36.75, 29.30, 25.11, 19.68, 14.16; ^31^P NMR (121 MHz, DMSO-*d*_6_, δ) −2.86.

## Supporting Information

File 1 Complete descriptions of the syntheses, including the precursors **THP-Cit-BZP-yne** and **DMT-Cit-Dod-NBD**, additional information about **α-4Ac-Man**, and the analytical data: NMR and HRMS spectra.
