# Supplementary material for: Nonenzymatic synthesis of anomerically pure, mannosyl-based molecular probes for scramblase identification studies
Source: Beilstein J Org Chem. 2020 Jul 20;16:1732–9. doi: 10.3762/bjoc.16.145 (PMC7385334; doi:10.3762/bjoc.16.145)

BEILSTEIN JOURNAL OF ORGANIC CHEMISTRY

Supporting Information

for

Nonenzymatic synthesis of anomerically pure, mannosyl-based molecular probes for scramblase identification studies

Giovanni Picca, Markus Probst, Simon M. Langenegger, Oleg Khorev, Peter Bütikofer, Anant K. Menon and Robert Häner

Beilstein J. Org. Chem. **2020,** *16,* 1732–1739. doi:10.3762/bjoc.16.145

Complete descriptions of the syntheses, including the precursors THP-Cit-BZP-yne and DMT-Cit-Dod-NBD, additional information about α-4Ac-Man, and the analytical data: NMR and HRMS spectra

License and Terms: This is a supporting information file under the terms of the Creative Commons Attribution License (http://creativecommons.org/licenses/by/4.0). Please note that the reuse, redistribution and reproduction in particular requires that the authors and source are credited.
The license is subject to the *Beilstein Journal of Organic Chemistry* terms and conditions: (https://www.beilstein-journals.org/bjoc)

Table of Contents

Overview Synthesis S3

Scheme 1: S3

Scheme 2: S4

General Procedures S5

Ratio of α- to β-anomer in commercially available tetra-*O*-acetylated D-Mannose S6

Procedures S7

Compound **THP-Cit-BZP-yne** S7

Compound **Cit-BZP-yne** S7

Compound **β-4Ac-Man-CEP** S8

Compound **MPC-1** S8

Compound **DMT-Cit** S9

Compound **DMT-Cit-OH** S9

Compound **DMT-Cit-Dod-TBDMS** S10

Compound **DMT-Cit-Dod** S10

Compound **DMT-Cit-Dod-Pht** S11

Compound **DMT-Cit-Dod-NH2** S11

Compound **DMT-Cit-Dod-NBD** S11

Compound **Cit-Dod-NBD** S12

Compound **MPC-2** S12

Compound **α-4Ac-Man-CEP** S13

Compound **MPC-3** S14

Analytical data NMR S15

Compound **THP-Cit-BZP-yne** S15

Compound **Cit-BZP-yne** S16

Compound **β-4Ac-Man-CEP** S17

Compound **β-4Ac-Man-CEP** S18

Compound **MPC-1** S18

Compound **MPC-1** S19

Compound **MPC-1** S20

Compound **DMT-Cit** S21

Compound **DMT-Cit-OH** ... S22
Compound **DMT-Cit-Dod-TBDMS** ... S23
Compound **DMT-Cit-Dod** ... S24
Compound **DMT-Cit-Dod-Pht** ... S25
Compound **DMT-Cit-Dod-NH2** ... S26
Compound **DMT-Cit-Dod-NBD** ... S27
Compound **Cit-Dod-NBD** ... S28
Compound **MPC-2** ... S29
Compound **MPC-2** ... S30
Compound **MPC-2** ... S31
Compound **α-4Ac-Man-CEP** ... S32
Compound **α-4Ac-Man-CEP** ... S33
Compound **MPC-3** ... S33
Compound **MPC-3** ... S34
Compound **MPC-3** ... S35
Analytical data RP-HPLC ... S36
Compound **MPC-2** ... S36
Data MS analysis ... S37
Compounds **THP-Cit-BZP-yne**, **Cit-BZP-yne** and **β-4Ac-Man-CEP** ... S37
Compounds **MPC-1**, **DMT-Cit** and **DMT-Cit-OH** ... S38
Compounds **DMT-Cit-Dod-TBDMS**, **DMT-Cit-Dod** and **DMT-Cit-Dod-Pht** ... S39
Compounds **DMT-Cit-Dod-NH2**, **DMT-Cit-Dod-NBD** and **Cit-Dod-NBD** ... S40
Compounds **MPC-2** and **MPC-3** ... S41

Overview Synthesis

The synthetic steps are outlined in Schemes 1 and 2:

Scheme 1:

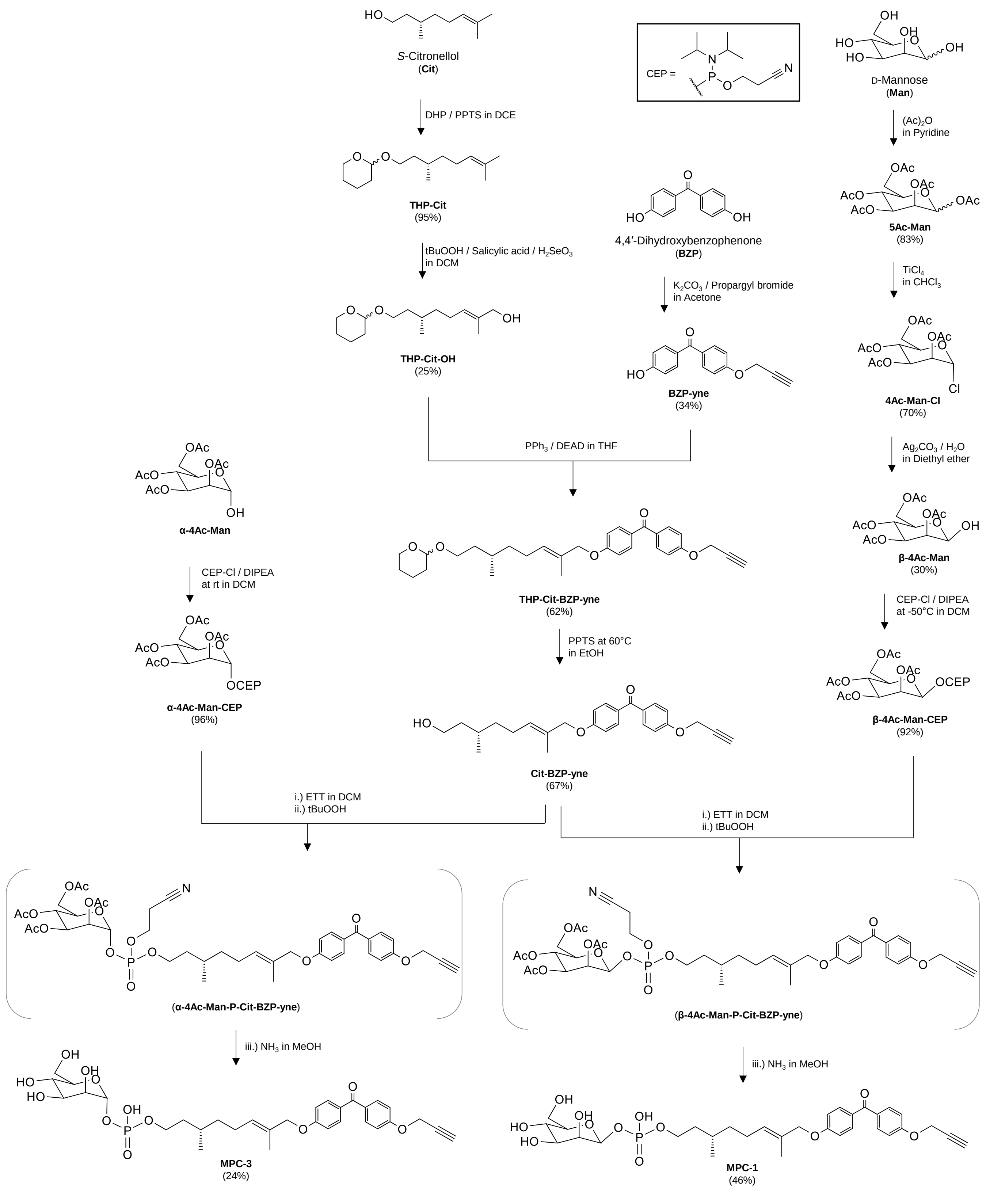

Scheme 2:

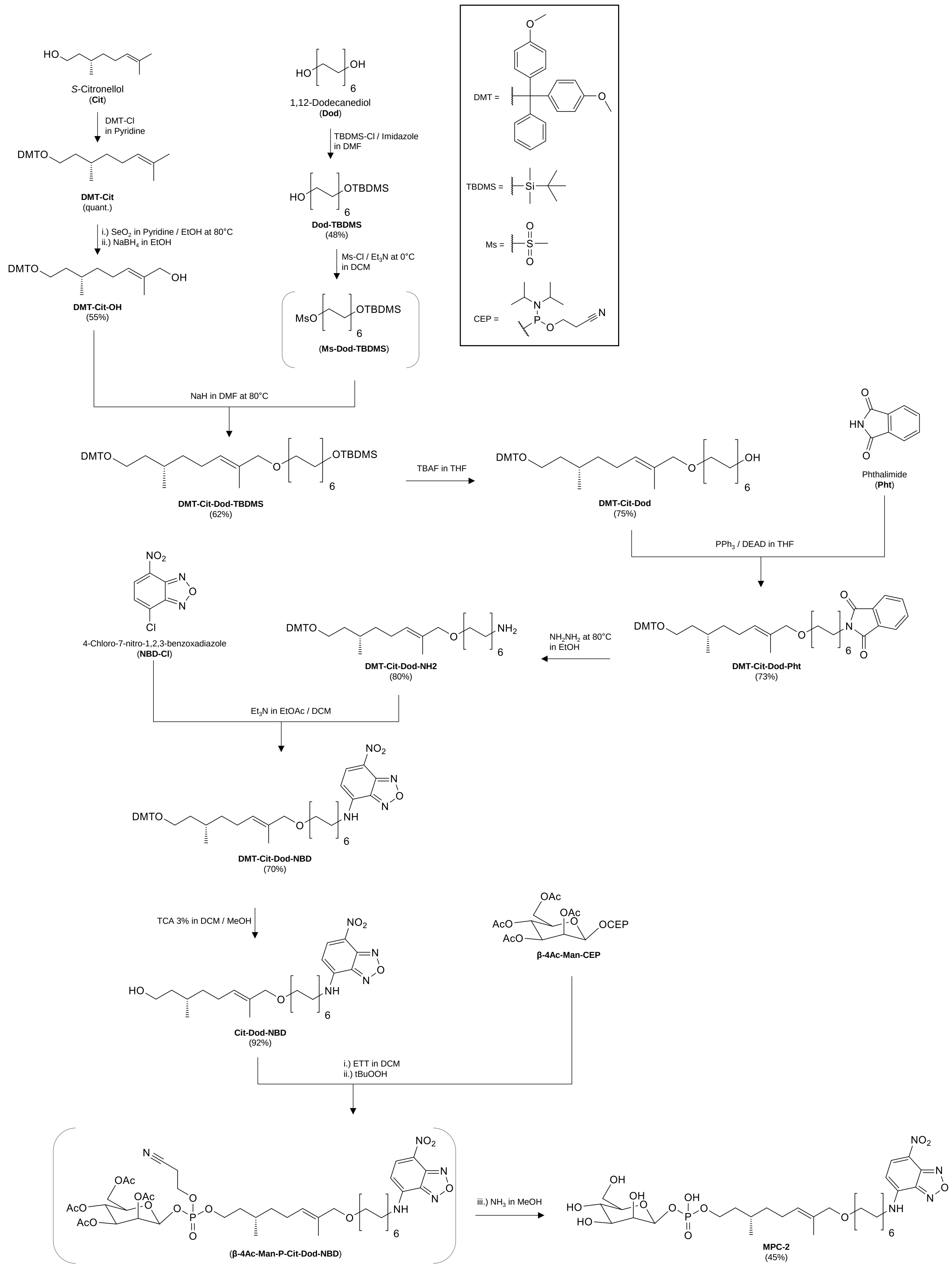

S-Citronellol
(Cit)
DMT-Cl
in Pyridine
DMT-Cit
(quant.)
i.) SeO_2 in Pyridine / EtOH at 80°C
ii.) $NaBH_4$ in EtOH
DMT-Cit-OH
(55%)
1,12-Dodecanediol
(Dod)
TBDMS-Cl / Imidazole
in DMF
Dod-TBDMS
(48%)
Ms-Cl / Et_3N at 0°C
in DCM
(Ms-Dod-TBDMS)
DMT =
TBDMS =
Ms =
CEP =
NaH in DMF at 80°C
DMT-Cit-Dod-TBDMS
(62%)
TBAF in THF
DMT-Cit-Dod
(75%)
Phthalimide
(Pht)
PPh_3 / DEAD in THF
DMT-Cit-Dod-Pht
(73%)
NH_2NH_2 at 80°C
in EtOH
DMT-Cit-Dod-NH2
(80%)
4-Chloro-7-nitro-1,2,3-benzoxadiazole
(NBD-Cl)
Et_3N in EtOAc / DCM
DMT-Cit-Dod-NBD
(70%)
TCA 3% in DCM / MeOH
Cit-Dod-NBD
(92%)
β-4Ac-Man-CEP
i.) ETT in DCM
ii.) tBuOOH
(β-4Ac-Man-P-Cit-Dod-NBD)
iii.) NH_3 in MeOH
MPC-2
(45%)

General Procedures

S-Citronellol (**Cit**), 4,4′ -Dihydroxybenzophenone (**BZP**), D-Mannose (**Man**), 1,12-Dodecanediol (**Dod**) and the 2,3,4,6-Tetra-*O*-acetyl-D-mannopyranose (**α-4Ac-Man**) are commercially available. Compounds **THP-Cit** and **THP-Cit-OH**;[1,2] **BZP-yne**;[3] **5Ac-Man**, **4Ac-Man-Cl**, and **β-4Ac-Man**;[4,5] and **Dod-TBDMS**;[6] were prepared according to published procedures.
The synthesis of ***α*-4Ac-Man-CEP** is described in the literature.[7] Its analytical data (^{1}H-, ^{13}C and ^{31}P-NMR), however, are published herein for the first time (see below), making the comparison with ***β*-4Ac-Man-CEP** easier.
NMR spectra were recorded on a Bruker Avance III HD 300 or Avance II 400 / Avance III HD 400. MS data were obtained from a ThermoScientific LTQ Orbitrap XL, equipped with a Nanoelectrospray Ion Source (NSI). Analytical and semi-preparative RP-HPLC runs were performed on a Shimadzu (LC-10ADvp with SPD-M10Avp and a C18, 5 µm column from Dr. Maisch GmbH). All chemicals and reagents were purchased from commercial suppliers (Sigma-Aldrich Chemie GmbH, Buchs CH; TCI Deutschland GmbH; Dr. Grogg Chemie AG, Stettlen-Deisswil CH; VWR International GmbH), if not otherwise stated. For better visualization (where necessary) of the spots on the thin-layer chromatography (TLC) plates, the following staining methods were applied: Vanillin stain (5% ethanolic solution of sulfuric acid; 1% ethanolic vanillin solution) for compounds containing terpenoids. Carbohydrates (and derivatives) were stained with "Cerium-Molybdate" (40 mM aqueous ammonium molybdate tetrahydrate; 4 mM aqueous ammonium cerium(IV) nitrate; ca. 8% sulfuric acid).
2D HSQC NMR: The HSQC (phased) pulse sequence was set to default pulse sequence (from the Bruker library "hsqcedetgpsisp2.2"). 2D ^{1}H/^{13}C correlation via double inept transfer using sensitivity improvement and phase sensitive using Echo/Antiecho-TPPI gradient selection (no ^{13}C decoupling during acquisition).

[1] **J. S. Rush, T. Subramanian, K. L. Subramanian, F. O. Onono, C. J. Waechter, H. P. Spielmann**. Curr. Chem. Biol., **2015**, *9(2)*, 123–141.

[2] **Y.-W. Wu, K. Alexandrov, L. Brunsveld**. Nature Protocols, **2007**, *2(11)*, 2704–2711.

[3] **B. Lee, W. Sun, H. Lee, H. Basavarajappa, R. S. Sulaiman, K. Sishtla, X. Fei, T. W. Corson, S.-Y. Seo**. Bioorganic & Medicinal Chemistry Letters, **2016**, *26*, 4277–4281.

[4] **W. A. Bonner**. J. Am. Chem. Soc., **1958**, *80(13)*, 3372–3379.

[5] **C. D. Warren, I. Y. Liu, A. Herscovics, R. W. Jeanloz**. J. Biol. Chem., **1975**, *250*, 8069–8078.

[6] **T. Ren, G. Zhang, D. Liu**. Tetrahedron Letters, **2001**, *42(6)*, 1007–1010.

[7] **G. A. Elsayed, G.-J. Boons**. Synlett, **2003**, *9*, 1373–1375.

Ratio of α- to β-anomer in commercially available tetra-*O*-acetylated D-Mannose

The commercially available 2,3,4,6-Tetra-*O*-acetyl-D-mannopyranose (Sigma-Aldrich, product number: 772283) consists almost exclusively of the α-anomer, as indicated by NMR measurements (in solution, see Figure below). Hence, the designation **α-4Ac-Man**. In contrast, the β-anomer (**β-4Ac-Man**) was obtained in 3 steps from D-Mannose (**Man**), leading to anomerically pure starting material for the subsequent phosphitylation reaction. Performing this reaction at lower temperatures (≤ -15°C) inhibits anomerization, and thus, the configuration is retained once the material is converted into the corresponding phosphoramidite.

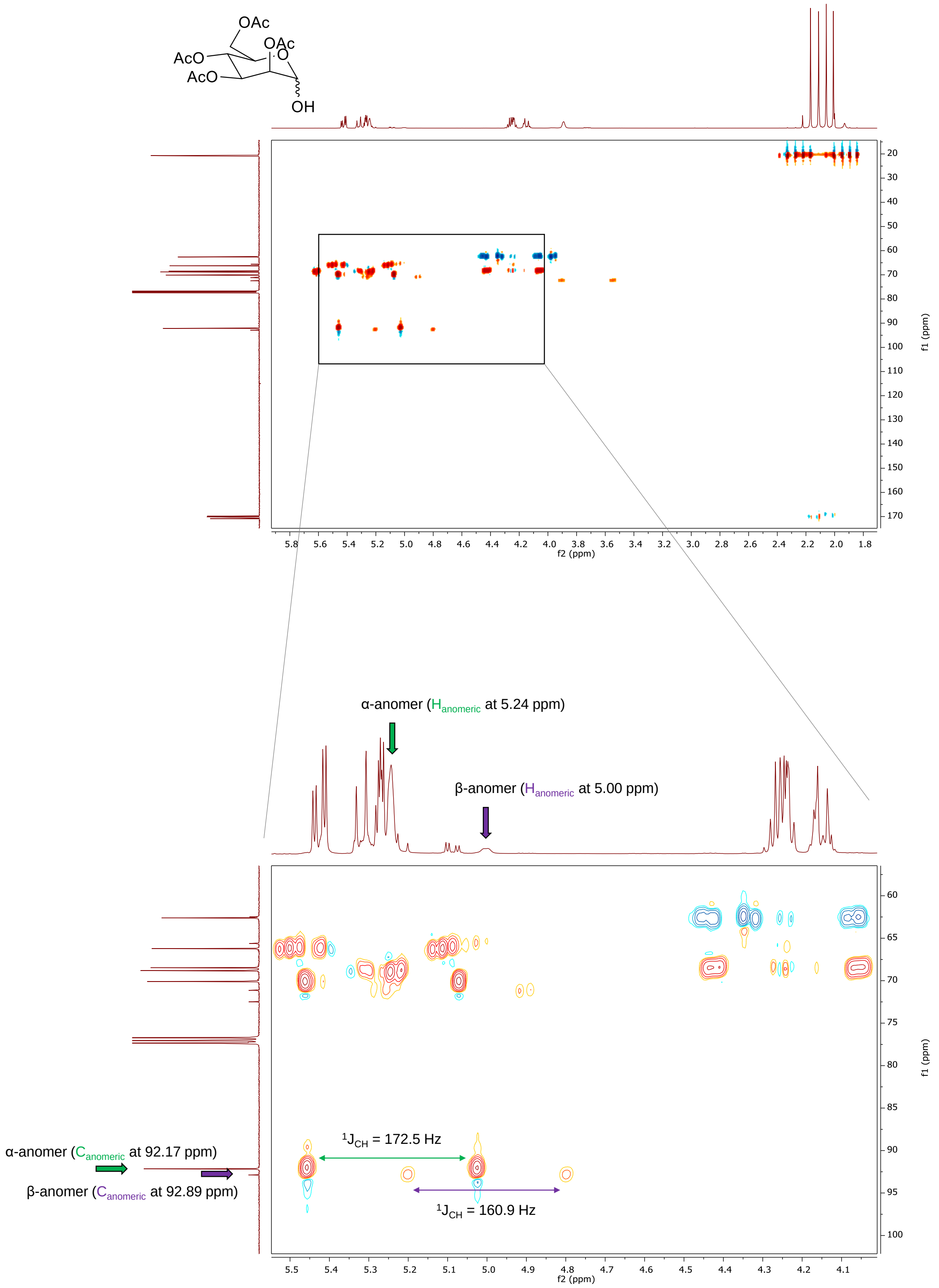

Procedures

*Compound **THP-Cit-BZP-yne***

Diethylazodicarboxylate (DEAD) solution (254 µL, 0.59 mmol, 1 eq; ca. 40% in toluene) was added at 0°C to triphenylphosphine (PPh_3) (153 mg, 0.58 mmol, 1 eq; dissolved in 3 mL dry THF). Then, **THP-Cit-OH** (149 mg, 0.58 mmol, 1 eq) and **BZP-yne** (147 mg, 0.58 mmol, 1 eq; dissolved in 3 mL dry THF) were added to the mixture. The reaction was stirred overnight at rt under argon. The orange solution was concentrated under reduced pressure on a rotary evaporator and purified via an automated flash chromatography system (Biotage Isolera Four; 20 g silica gel SNAP cartridge; using an automatically generated gradient of ethyl acetate/n-hexane for elution). The fractions containing the product (R_f = 0.30; ethyl acetate/n-hexane 1:5) were combined and the solvents were removed under reduced pressure on a rotary evaporator, to give **THP-Cit-BZP-yne** (177 mg, 0.36 mmol, 62% yield) as a colorless oil.

^1H NMR ($CDCl_3$, 300 MHz): δ (ppm) 7.85 – 7.72 (m, 4H), 7.09 – 6.99 (m, 2H), 7.02 – 6.91 (m, 2H), 5.61 – 5.50 (m, 1H), 4.77 (d, J=2.4 Hz, 2H), 4.61 – 4.52 (m, 1H), 4.46 (s, 2H), 3.93 – 3.70 (m, 2H), 3.57 – 3.43 (m, 1H), 3.48 – 3.32 (m, 1H), 2.56 (t, J=2.4 Hz, 1H), 2.21 – 1.98 (m, 2H), 1.91 – 1.76 (m, 1H), 1.76 – 1.64 (m, 4H), 1.70 – 1.44 (m, 7H), 1.48 – 1.34 (m, 1H), 1.33 – 1.17 (m, 1H), 0.92 (d, J=6.4 Hz, 3H).

^{13}C NMR ($CDCl_3$, 75 MHz): δ (ppm) 194.44, 162.45, 160.63, 132.23, 132.18, 132.12, 131.67, 130.47, 130.21, 129.95, 114.36, 114.33, 99.05, 98.88, 77.93, 76.10, 74.26, 65.93, 65.84, 62.43, 55.89, 36.70, 36.56, 30.82, 29.74, 25.52, 25.22, 19.72, 19.64, 19.54, 13.84.

HR-MS (NSI): calc. m/z for $[C_{31}H_{38}O_5 + Na^+]^+$ = 513.26, found m/z = 513.2612.

*Compound **Cit-BZP-yne***

THP-Cit-BZP-yne (279 mg, 0.57 mmol, 1 eq) was dissolved in anhydrous ethanol (8 mL) and pyridinium-p-toluenesulfonate (PPTS) (286 mg, 1.14 mmol, 2 eq) was added. The solution was stirred at 60°C for 2.5 h. The reaction mixture (at rt) was poured into a separating funnel containing diethyl ether (40 mL) and brine (16 mL). The organic layer was dried over $MgSO_4$ and the volatile components were removed under reduced pressure. The yellowish residue (crude product) was purified by flash chromatography on silica gel with a mixture of n-hexane/ethyl acetate (3:2) as eluent. The fractions containing the product (R_f = 0.22; n-hexane/ethyl acetate 3:2) were combined and the solvents were removed under reduced pressure on a rotary evaporator, to give **Cit-BZP-yne** (154 mg, 0.38 mmol, 67% yield) as a white solid.

^1H NMR ($CDCl_3$, 300 MHz): δ (ppm) 7.85 – 7.72 (m, 4H), 7.09 – 6.99 (m, 2H), 7.02 – 6.91 (m, 2H), 5.61 – 5.49 (m, 1H), 4.77 (d, J=2.4 Hz, 2H), 4.47 (s, 2H), 3.69 (m, 2H), 2.56 (t, J=2.4 Hz, 1H), 2.21 – 1.98 (m, 2H), 1.74 (s, 3H), 1.70 – 1.50 (m, 1H), 1.50 – 1.15 (m, 4H), 0.92 (d, J=6.4 Hz, 3H).

^{13}C NMR ($CDCl_3$, 75 MHz): δ (ppm) 194.48, 162.39, 160.64, 132.22, 132.14, 131.64, 130.51, 130.29, 129.82, 114.37, 114.36, 77.92, 76.10, 74.21, 61.12, 55.89, 39.83, 36.63, 29.18, 25.20, 19.46, 13.85.

HR-MS (NSI): calc. m/z for $[C_{26}H_{30}O_4 + Na^+]^+$ = 429.20, found m/z = 429.2025.

*Compound **β-4Ac-Man-CEP***

β-4Ac-Man (100 mg, 0.29 mmol, 1 eq) was dissolved in anhydrous DCM (4 mL) at -60°C. Then, N,N-diisopropylethylamine (DIPEA) (75 µL, 0.43 mmol, 1.5 eq) was added, followed by the addition of 2-cyanoethyl N,N-diisopropylchlorophosphoramidite (CEP-Cl) (77 µL, 0.35 mmol, 1.2 eq). The mixture was stirred for 30 min at -50°C under argon. The crude product was then directly purified by flash chromatography on silica gel using ethyl acetate/n-hexane 1:1 + 2% triethylamine (Et_3N). The fractions containing the product (R_f = 0.40) were combined and the solvents were removed under reduced pressure on a rotary evaporator. The product was extensively dried to give **β-4Ac-Man-CEP** (145 mg, 0.26 mmol, 92% yield) of a colorless foam.

^{1}H NMR ($CDCl_3$, 300 MHz): δ (ppm) 5.37 (dd, J=25.2, 3.3 Hz, 1H), 5.19 (t, J=9.9 Hz, 1H), 5.12 – 4.95 (m, 2H), 4.24 – 4.09 (m, 2H), 3.91 – 3.48 (m, 5H), 2.66 – 2.53 (m, 2H), 2.14 (s, 3H), 2.05 – 2.00 (m, 8H), 1.95 (d, J=1.6 Hz, 3H), 1.12 (dd, J=10.8, 6.9 Hz, 12H).

^{13}C NMR ($CDCl_3$, 75 MHz): δ (ppm) 170.58, 170.55, 170.31, 170.10, 169.98, 169.94, 169.67, 169.63, 117.63, 93.69, 93.57, 93.36, 72.72, 72.66, 70.98, 69.78, 69.69, 65.99, 65.90, 62.67, 62.43, 59.20, 58.96, 58.78, 58.53, 44.07, 43.90, 43.77, 43.59, 24.61, 24.52, 24.43, 24.34, 24.04, 23.94, 20.80, 20.77, 20.68, 20.56, 20.34, 20.25, 20.14, 20.06.

^{31}P NMR ($CDCl_3$, 121 MHz): δ (ppm) 153.31, 150.00.

HR-MS (NSI): calc. m/z for $[C_{23}H_{37}N_2O_{11}P + H^+]^+$ = 549.22, found m/z = 549.2201.

*Compound **MPC-1***

5-(Ethylthio)-1H-tetrazole (ETT) (72 mg, 0.55 mmol, 1.5 eq) was dissolved in anhydrous DCM (2 mL), combined with **β-4Ac-Man-CEP** (223 mg, 0.41 mmol, 1.1 eq) and added to **Cit-BZP-yne** (150 mg, 0.37 mmol, 1 eq; dissolved in 2 mL anhydrous DCM). The reaction mixture was stirred for 30 min at rt under argon. Afterwards, tert-butyl hydroperoxide (tBuOOH) solution (201 µL, 1.11 mmol, 3 eq; ~5.5 M in decane) was added and the mixture was stirred for an additional 15 min. The reaction mixture was diluted with toluene (60 mL), then washed with saturated sodium hydrogen carbonate solution (30 mL) and brine (30 mL). The organic layer was dried over $MgSO_4$ and the volatile components were removed under reduced pressure. The resulting residue was dissolved in methanol and purified by PLC (using ethyl acetate/n-hexane 2:1 as mobile phase). The broad band, located at the very bottom of the plate (R_f ≈ 0.10, on a TLC using ethyl acetate/n-hexane 1:1) was removed and intermediate **β-4Ac-Man-P-Cit-BZP-yne** was extracted with methanol (150 mL). The solvent was removed under reduced pressure on a rotary evaporator. Then, an ammonia solution (3 mL; 2.0 M in methanol) was added and the reaction mixture was stirred overnight at rt. The resulting solution was first diluted with methanol (2 mL) and the volatile components were removed under reduced pressure, then, Milli-Q water (2 mL) was added and the material was lyophilized overnight to afford **MPC-1** (110 mg, 0.17 mmol, 46% yield) as a slightly yellowish solid.

^{1}H NMR (DMSO-d_6, 400 MHz): δ (ppm) 7.75 – 7.66 (m, 4H), 7.17 – 7.04 (m, 4H), 5.58 (t, J=6.7 Hz, 1H), 4.92 (d, J=2.4 Hz, 2H), 4.87 (d, J=8.5 Hz, 1H), 4.51 (s, 2H), 3.77 – 3.61 (m, 5H), 3.62 – 3.32 (m, 2H), 3.33 – 3.25 (m, 2H), 2.05 (m, 2H), 1.69 (s, 3H), 1.62 – 1.48 (m, 2H), 1.42 – 1.26 (m, 2H), 1.26 – 1.13 (m, 1H), 0.87 (d, J=6.2 Hz, 3H).

^{13}C NMR (DMSO-d_6, 101 MHz): δ (ppm) 193.64, 162.46, 160.86, 132.29, 132.13, 131.24, 130.63, 130.30, 129.66, 115.07, 115.02, 96.07, 96.04, 79.25, 79.20, 74.05, 73.98, 71.53, 71.48, 67.39, 63.02, 62.97, 61.82, 56.20, 37.85, 37.78, 36.76, 36.36, 29.33, 25.14, 19.69, 14.17.

^{31}P NMR (DMSO-d_6, 121 MHz): δ (ppm) 25.54, 4.74, -2.89.

HR-MS (NSI): calc. m/z for $[C_{32}H_{41}O_{12}P - H^+]^-$ = 647.23, found m/z = 647.2258.

*Compound **DMT-Cit***

4,4'-dimethoxytrityl chloride (DMT-Cl) (2.36 g, 7.04 mmol, 1.1 eq) was added to a solution of *S*-Citronellol (**Cit**) (1 g, 6.4 mmol) in dry pyridine (35 mL). The mixture was stirred for 6 h. The solvent was removed and the resulting oil was dissolved in DCM, washed with aq. citric acid solution (10%) and dried with Na_2SO_4. The crude product was purified by column chromatography on silica gel (hexane/ethyl acetate 9:1), to yield **DMT-Cit** (2.93 g, quant.) as a pale, yellow oil (R_f = 0.8; hexane/EtOAc 85:15).

^1H NMR (300 MHz, DMSO-d_6) δ 7.38 – 7.20 (m, 9H), 6.87 (d, J = 8.5 Hz, 4H), 5.04 (t, J = 7.1 Hz, 1H), 3.72 (s, 6H), 3.04 – 2.85 (m, 2H), 1.95 – 1.85 (m, 2H), 1.62 (s, 3H), 1.61 – 1.55 (m, 2H), 1.53 (s, 3H), 1.45 – 1.29 (m, 1H), 1.29 – 1.12 (m, 1H), 1.13 – 0.95 (m, 1H), 0.75 (d, J = 6.3 Hz, 3H).

^{13}C NMR (75 MHz, DMSO-d_6) δ 158.44, 145.76, 136.53, 130.91, 130.03, 128.20, 128.09, 127.00, 125.06, 117.48, 113.54, 85.65, 61.29, 55.45, 36.94, 36.79, 29.30, 25.96, 25.34, 19.98, 17.94.

HRMS (ESI): calc. m/z for $[C_{31}H_{38}O_3 + Na^+]^+$ = 481.28, found m/z = 481.27.

*Compound **DMT-Cit-OH***

DMT-Cit (1.02 g, 2.22 mmol) was dissolved in a mixture of pyridine/EtOH (10:1, 40 mL) under argon at rt. The mixture was stirred for 20 min. Then, SeO_2 (245 mg, 2.22 mmol, 1 eq) was added, the mixture was heated to 80°C and stirred overnight. Afterwards, the solvents were removed under vacuum, the resultant oil was dissolved in ethyl acetate, washed with brine and dried with Na_2SO_4. The solvent was removed and the crude product was re-dissolved in EtOH (30 mL) at 0°C. $NaBH_4$ (168 mg, 2 eq) was added and the mixture was stirred for 1 h. Then, H_2O was added, the crude product was extracted with DCM, dried with Na_2SO_4 and purified by column chromatography on silica gel (R_f = 0.2; hexane/EtOAc 8:2). **DMT-Cit-OH** (580 mg, 55% yield) was obtained as a yellow oil.

^1H NMR (400 MHz, DMSO-d_6) δ 7.38 – 7.20 (m, 9H), 6.88 (d, J = 8.9 Hz, 4H), 5.28 (t, J = 7.7 Hz, 1H), 4.62 (t, J = 5.6 Hz, 1H), 3.77 (d, J = 5.3 Hz, 2H), 3.72 (s, 6H), 3.02 – 2.94 (m, 2H), 1.97 – 1.91 (m, 2H), 1.64 – 1.54 (m, 2H), 1.52 (s, 3H), 1.41 – 1.30 (m, 1H), 1.29 – 1.18 (m, 2H), 1.11 – 1.06 (m, 1H), 0.76 (d, J = 6.4 Hz, 3H).

^{13}C NMR (101 MHz, DMSO-d_6) δ 158.45, 145.74, 136.56, 136.54, 135.68, 130.03, 128.21, 128.11, 127.01, 124.07, 113.56, 85.68, 66.97, 61.35, 55.46, 36.86, 36.80, 29.42, 24.84, 19.95, 13.93.

HRMS (ESI): calc. m/z for $[C_{31}H_{38}O_4 + Na^+]^+$ = 497.28, found m/z = 497.27.

Compound ***DMT-Cit-Dod-TBDMS***

First, the intermediate **Ms-Dod-TBDMS** was prepared: **Dod-TBDMS** (750 mg, 2.37 mmol = 1 eq) was dissolved in DCM (10 mL) at 0°C under argon. Et_3N (1.65 mL, 11.85 mmol, 5 eq) was added, followed by the addition of methanesulfonyl chloride (Ms-Cl) (0.37 mL, 4.74 mmol, 2 eq). The reaction mixture was allowed to slowly reach rt, and the mixture was stirred for 1 h. The mixture was washed with brine, the organic phase was dried with Na_2SO_4 and the volatile components were removed under reduced pressure. The resultant oil (**Ms-Dod-TBDMS**) was used in the next reaction step without any further purification.

DMT-Cit-OH (937 mg, 1.97 mmol = 1 eq) was dissolved in DMF (10 mL) under argon at rt. NaH (237 mg, 5.92 mmol, 3 eq; 60% dispersion in mineral oil) was added and the reaction mixture was stirred for 30 min. Then, **Ms-Dod-TBDMS** (935 mg, 2.37 mmol, 1.2 eq; see above) was dissolved in DMF (3 mL) under argon at rt and added to the reaction mixture *via* cannula. The mixture was stirred for 4 h at 80°C. Afterwards, H_2O was added, the crude product was extracted with ethyl acetate, dried with Na_2SO_4 and finally, purified by column chromatography on silica gel (hexane/EtOAc 85:15). **DMT-Cit-Dod-TBDMS** (943 mg, 62% yield) was obtained as a pale, yellow oil (R_f = 0.6; hexane/EtOAc 8:2).

^{1}H NMR (300 MHz, DMSO-*d$_6$*) δ 7.38 – 7.21 (m, 9H), 6.87 (d, J = 8.3 Hz, 5H), 5.29 (t, J = 6.9 Hz, 1H), 3.73 (s, 8H), 3.58 – 3.52 (m, 2H), 3.23 (t, J = 6.6 Hz, 2H), 3.00 – 2.93 (m, 2H), 1.96 – 1.85 (m, 2H), 1.61 – 1.55 (m, 2H), 1.52 (d, J = 4.4 Hz, 3H), 1.48 – 1.35 (m, 5H), 1.24 (m, 19H), 1.08 (t, J = 7.0 Hz, 3H), 0.85 (s, 9H), 0.76 (d, J = 6.3 Hz, 3H), 0.00 (s, 6H).

^{13}C NMR (75 MHz, DMSO-*d$_6$*) δ 158.44, 145.72, 136.55, 130.01, 128.21, 128.10, 113.55, 85.66, 68.37, 55.46, 40.83, 40.56, 40.28, 40.00, 39.72, 39.45, 39.17, 36.68, 32.68, 29.34, 26.28, 26.24, 25.72, 25.69, 19.95, 18.43, 13.68, -4.84.

HRMS (ESI): calc. m/z for $[C_{49}H_{76}O_5Si + Na^+]^+$ = 795.55, found m/z = 795.54.

Compound ***DMT-Cit-Dod***

DMT-Cit-Dod-TBDMS (943 mg, 1.22 mmol) was dissolved in THF (10 mL) under argon at rt. The tetra-n-butylammonium fluoride (TBAF) (462 mg, 1.46 mmol, 1.2 eq) was added and the mixture was stirred for 6 h. The solvent was removed, the remaining oil dissolved in DCM and washed with brine. The organic phase was dried with Na_2SO_4 and purified by column chromatography on silica gel (R_f = 0.2; hexane/EtOAc 8:2), to give **DMT-Cit-Dod** (603 mg, 75% yield) as a yellow oil.

^{1}H NMR (300 MHz, DMSO-*d$_6$*) δ 7.41 – 7.21 (m, 9H), 6.87 (d, J = 8.9 Hz, 4H), 5.30 (t, J = 6.9 Hz, 1H), 4.30 (t, J = 5.1 Hz, 1H), 3.73 (s, 8H), 3.39 – 3.34 (m, 2H), 3.23 (t, J = 6.4 Hz, 2H), 2.97 (m, 2H), 1.97-1.92 (m, 2H), 1.63-1.55 (m, 2H), 1.53 (s, 3H), 1.43-1.34 (m, 5H), 1.22 (m, 19H), 1.10 (t, J = 4.6 Hz, 1H), 0.76 (d, J = 6.3 Hz, 3H).

^{13}C NMR (75 MHz, DMSO-*d$_6$*) δ 158.44, 145.72, 136.53, 130.02, 128.21, 128.09, 117.53, 113.55, 85.65, 61.19, 55.47, 36.57, 33.02, 29.57, 29.47, 25.98, 19.94.

HRMS (ESI): calc. m/z for $[C_{43}H_{62}O_5 + Na^+]^+$ = 681.46, found m/z = 681.49.

Compound ***DMT-Cit-Dod-Pht***

DMT-Cit-Dod (1.07 g, 1.57 mmol), PPh_3 (670 mg, 2.52 mmol, 1.6 eq) and phthalimide (Pht) (347 mg, 2.36 mmol, 1.5 eq) were dissolved in THF (40 mL). Then, DEAD solution (1.15 mL, 2.52 mmol, 1.6 eq; 40% in toluene) was added at rt under argon. The reaction mixture was stirred for 2 h. Afterwards, the volatile components were removed under reduced pressure on a rotary evaporator, the resulting oil was re-dissolved in ethyl acetate and washed with brine. The organic phase was dried with Na_2SO_4 and purified by column chromatography on silica gel (hexane/EtOAc 8:2; R_f = 0.5). **DMT-Cit-Dod-Pht** (891 mg, 73% yield) was obtained as a yellow oil.

^{1}H NMR (300 MHz, DMSO-d_6) δ 7.84 (d, J = 162.3 Hz, 5H), 7.38 – 7.18 (m, 9H), 6.86 (dd, J = 613.1, 8.9 Hz, 4H), 5.29 (t, J = 6.9 Hz, 1H), 3.72 (s, 8H), 3.54 (t, J = 7.1 Hz, 2H), 3.22 (t, J = 6.4 Hz, 2H), 3.03 – 2.85 (m, 2H), 1.98 – 1.86 (m, 2H), 1.60 – 1.49 (m, 7H), 1.49 – 1.38 (m, 2H), 1.27 – 1.16 (m, 18H), 1.10 – 1.03 (m, 1H), 0.74 (d, J = 6.2 Hz, 3H).

^{13}C NMR (75 MHz, DMSO-d_6) δ 169.70, 168.36, 158.42, 145.71, 136.52, 134.79, 133.09, 132.48, 132.05, 130.00, 128.17, 128.09, 127.35, 126.97, 123.54, 123.42, 123.39, 113.51, 85.64, 76.17, 69.11, 61.28, 55.43, 40.84, 40.56, 40.28, 40.00, 39.73, 39.45, 39.17, 37.82, 36.78, 36.58, 29.59, 29.37, 29.30, 29.23, 29.00, 28.33, 26.69, 26.17, 24.87, 19.90, 14.04.

HRMS (ESI): calc. m/z for $[C_{51}H_{65}NO_6 + Na^+]^+$ = 810.47, found m/z = 810.47.

Compound ***DMT-Cit-Dod-NH2***

DMT-Cit-Dod-Pht (2.44 g, 3.13 mmol) was dissolved in EtOH (50 mL) under argon at rt. Then, hydrazine monohydrate (0.61 mL, 12.54 mmol, 4 eq) was added and the reaction mixture was stirred under reflux overnight. The mixture was diluted with ethyl acetate and washed with aq. Na_2CO_3 solution (2 M). The organic phase was dried over Na_2SO_4 and the volatile components were removed under reduced pressure to give a white solid (R_f = 0.4; DCM/MeOH 9:1). The crude product (1.67 g, 80% yield) was used for the next reaction without further purification.

^{1}H NMR (300 MHz, DMSO-d_6) δ 7.40 – 7.19 (m, 15H), 6.87 (d, J = 8.9 Hz, 7H), 5.30 (t, J = 6.9 Hz, 1H), 3.72 (s, 12H), 3.35 – 3.18 (m, 3H), 3.04 – 2.89 (m, 4H), 1.99 – 1.85 (m, 3H), 1.65 – 1.50 (m, 8H), 1.43 (s, 5H), 1.22 (s, 40H), 0.92 – 0.83 (m, 2H), 0.74 (d, J = 4.2 Hz, 6H).

^{13}C NMR (75 MHz, DMSO-d_6) δ 158.43, 145.72, 136.53, 130.01, 128.09, 127.37, 127.01, 123.15, 113.53, 85.65, 69.12, 61.36, 61.29, 55.46, 29.49, 26.93, 26.19, 24.88, 19.93, 14.07.

HRMS (ESI): calc. m/z for $[C_{43}H_{63}NO_4 + H^+]^+$ = 658.48, found m/z = 658.4828.

Compound ***DMT-Cit-Dod-NBD***

DMT-Cit-Dod-NH2 (905 mg, 1.38 mmol) was dissolved in a mixture of EtOAc/DCM (3:1, 12 mL) under argon at rt. Then, 4-chloro-7-nitrobenzofurazan (NBD-Cl) (357 mg, 1.79 mmol, 1.3 eq) was added and the mixture was stirred for 1 h. Next, Et_3N (0.25 mL, 1.79 mmol, 1.3 eq) was added and the reaction mixture was stirred for 3 h. Afterwards, the mixture was diluted with DCM, washed with brine and the organic phase was dried with Na_2SO_4. The crude product was purified by column chromatography on silica gel (hexane/EtOAc 7:3) to give **DMT-Cit-Dod-NBD** (787 mg, 70% yield) as a deep-red oil (R_f = 0.7; DCM/MeOH 9:1).

^{1}H NMR (300 MHz, DMSO-d_6) δ 9.55 (s, 1H), 8.50 (d, J = 9.0 Hz, 1H), 7.43 – 7.18 (m, 9H), 6.87 (d, J = 8.9 Hz, 4H), 6.39 (d, J = 9.0 Hz, 1H), 5.30 (t, J = 7.3 Hz, 1H), 3.73 (s, 8H), 3.52 – 3.40 (m, 2H), 3.23 (t, J = 6.4 Hz, 2H), 2.97 (m, 2H), 1.99 – 1.89 (m, 2H), 1.70 – 1.61 (m, 2H), 1.53 (s, 3H), 1.47 – 1.18 (m, 24H), 0.75 (d, J = 6.2 Hz, 3H).

^{13}C NMR (75 MHz, DMSO-d_6) δ 158.28, 148.83, 140.70, 130.36, 129.39, 128.70, 128.12, 127.87, 126.89, 114.14, 113.22, 80.37, 79.67, 55.46, 40.82, 29.41, 26.85.

HRMS (ESI): calc. m/z for $[C_{49}H_{64}N_4O_7 + Na^+]^+$ = 843.47, found m/z = 843.47.

*Compound **Cit-Dod-NBD***

DMT-Cit-Dod-NBD (787 mg, 0.96 mmol) was dissolved in a TCA (trichloroacetic acid) solution (3% in DCM/MeOH 1:1) at rt. The reaction mixture was stirred for 2 h. Then, the mixture was diluted with DCM, washed with brine and the organic phase was dried with Na_2SO_4. Purification by column chromatography on silica gel (hexane/EtOAc 6:4) gave **Cit-Dod-NBD** (455 mg, 92% yield) as a red oil (R_f = 0.5; hexane/EtOAc 1:1).

^{1}H NMR (300 MHz, DMSO-d_6) δ 9.55 (s, 1H), 8.50 (d, J = 8.8 Hz, 1H), 6.49 – 6.31 (m, 1H), 5.33 (t, J = 7.2 Hz, 1H), 4.28 (t, J = 5.1 Hz, 1H), 3.73 (s, 2H), 3.59 – 3.36 (m, 4H), 3.26 (t, J = 6.4 Hz, 2H), 1.74 – 1.61 (m, 2H), 1.55 (s, 3H), 1.52 – 1.40 (m, 4H), 1.24 (s, 19H), 0.84 (d, J = 6.5 Hz, 3H).

^{13}C NMR (75 MHz, DMSO-d_6) δ 132.41, 127.51, 99.47, 79.67, 76.21, 69.19, 63.78, 59.24, 37.01, 33.97, 30.68, 29.63, 29.47, 29.42, 29.18, 28.81, 28.06, 26.85, 26.19, 25.00, 24.92, 19.86, 19.09, 14.06, 13.99.

HRMS (ESI): calc. m/z for $[C_{28}H_{46}N_4O_5 + H^+]^+$ = 519.35, found m/z = 519.3539.

*Compound **MPC-2***

ETT (38 mg, 0.29 mmol, 1.5 eq) was dissolved in anhydrous DCM (2 mL), then combined with **β-4Ac-Man-CEP** (116 mg, 0.21 mmol, 1.1 eq) and added to **Cit-Dod-NBD** (100 mg, 0.19 mmol, 1 eq; dissolved in 1 mL anhydrous DCM). The reaction mixture was stirred for 30 min at rt under argon. Afterwards, tBuOOH solution (110 µL, 0.61 mmol, 3 eq; ~5.5 M in decane) was added and the mixture was stirred for an additional 15 min. The reaction mixture was poured into a separating funnel containing DCM (15 mL) and saturated sodium hydrogen carbonate solution (15 mL). The organic layer was further washed with brine (15 mL) and then dried with $MgSO_4$. The volatile components were removed under reduced pressure, the brownish residue was re-dissolved in acetonitrile (1.5 mL) and purified by PLC (using a mixture of ethyl acetate/n-hexane 1:1 as mobile phase). The broad band located at the very bottom of the plate (R_f ≈ 0.10, on a TLC using ethyl acetate/n-hexane 1:1) was removed and intermediate **β-4Ac-Man-P-Cit-Dod-NBD** was extracted with acetonitrile (180 mL). The solvent was removed under reduced pressure on a rotary evaporator. The material was then treated overnight with an ammonia solution (2 mL; 2.0 M in methanol). Afterwards, the reaction mixture was diluted with methanol (2 mL), the volatile components were removed under reduced pressure and the orange-brown residue (re-dissolved in 1.5 mL methanol) was purified by PLC (using chloroform/methanol/water 60:25:4 as the mobile phase). The broad band, centered at around an R_f of about 0.22, was removed and the product was extracted with methanol (170 mL). The solvent was removed on a rotary evaporator, and the residue was dissolved in Milli-Q water (1.3 mL) and lyophilized overnight, to give **MPC-2** (65 mg, 0.09 mmol, 45% yield).

^{1}H NMR (D_2O, 300 MHz): δ (ppm) 8.27 (s, 1H), 6.17 (s, 1H), 5.35 (s, 1H), 5.13 (d, J=8.4 Hz, 1H), 4.14 – 3.53 (m, 10H), 3.50 – 3.19 (m, 4H), 2.05 – 1.99 (m, 2H), 1.78 – 1.42 (m, 9H), 1.33 – 1.08 (m, 21H), 0.91 (d, J=6.0 Hz, 3H).

^{13}C NMR (D_2O, 101 MHz): δ (ppm) 131.98, 131.89, 131.83, 128.40, 128.33, 95.42, 95.38, 76.90, 76.65, 72.73, 71.17, 71.12, 69.33, 69.32, 66.19, 64.79, 61.20, 60.82, 37.11, 29.76, 29.61, 29.52, 29.39, 29.23, 26.26, 25.09, 18.99, 13.57.

^{31}P NMR (D_2O, 121 MHz): δ (ppm) 7.22, -1.64.

(HR-MS analysis, see below)

Note: Due to the apparent presence of a substantial amount of byproduct(s), estimated to be ⅓ (based on NMR data) and most likely resulting from degradation of the NBD-tag (when treated with alkaline conditions), further purification *via* RP-HPLC was necessary (see below).

RP-HPLC purification:
Ca. 20 mg of the obtained material (see above) were dissolved in 4 mL Milli-Q H_2O, to perform 4 runs (1 mL each). A gradient of: buffer A (95%)/MeCN (5%) → buffer A (0%)/MeCN (100%), within 30 min, was applied (buffer A = 25 mM ammonium hydrogen carbonate (NH_4HCO_3) in Milli-Q H_2O).
The collected fractions containing the main product (t_R = 21.04 min) were combined and lyophilized overnight, to yield a dark-orange, powder (ca. 8 mg).

HR-MS (NSI): calc. m/z for $[C_{34}H_{57}N_4O_{13}P - H^+]^-$ = 759.36, found m/z = 759.3590.

*Compound **α-4Ac-Man-CEP***

α-4Ac-Man (0.5 g, 1.44 mmol) was dissolved in anhydrous DCM (2 mL) and DIPEA (0.38 mL, 2.18 mmol, 1.5 eq) was added. Then, CEP-Cl (0.38 mL, 1.70 mmol, 1.2 eq) was added and the reaction mixture was stirred for 30 min at rt under argon. The crude product was directly purified by flash chromatography on silica gel using ethyl acetate/n-hexane 1:1 + 2% Et_3N as eluent. The fractions containing the product (R_f = 0.71; on a TLC, same solvent system as for the column) were combined and the solvents were removed on a rotary evaporator to give the product **α-4Ac-Man-CEP** (0.76 g = 1.39 mmol, 96% yield) as a colorless syrup.

^{1}H NMR ($CDCl_3$, 300 MHz): δ (ppm) 5.39 – 5.34 (m, 1H), 5.33 – 5.28 (m, 1H), 5.28 – 5.20 (m, 1H), 5.19 – 5.01 (m, 1H), 4.29 (td, J=12.4, 4.9 Hz, 1H), 4.21 – 4.05 (m, 2H), 3.95 – 3.77 (m, 2H), 3.77 – 3.44 (m, 2H), 2.66 (m, 2H), 2.16 (s, 3H), 2.10 – 2.04 (m, 7H), 1.99 (s, 3H), 1.24 – 1.16 (m, 12H).

^{13}C NMR (DMSO-d_6, 101 MHz): δ (ppm) 170.61, 170.53, 170.32, 170.22, 170.17, 170.11, 170.04, 119.32, 119.28, 92.89, 92.71, 92.60, 92.37, 70.19, 70.14, 69.99, 69.96, 69.51, 69.12, 68.82, 68.78, 65.69, 65.63, 62.45, 62.39, 59.41, 59.32, 59.21, 59.13, 55.27, 43.72, 43.68, 43.59, 43.55, 24.69, 24.65, 24.62, 21.03, 20.92, 20.88, 20.86, 20.82, 20.80, 20.27, 20.25, 20.19, 14.51.

^{31}P NMR ($CDCl_3$, 121 MHz): δ (ppm) 151.81, 149.61.

*Compound **MPC-3***

α-4Ac-Man-CEP (252 mg, 0.46 mmol, 1.1 eq) and ETT (82 mg, 0.63 mmol, 1.5 eq) were dissolved in anhydrous DCM (1 mL) and combined with **Cit-BZP-yne** (170 mg, 0.42 mmol, 1 eq; dissolved in 1.5 mL anhydrous DCM). The reaction mixture was stirred for 30 min at rt under argon. Afterwards, tBuOOH solution (182 µL, 1.31 mmol, 3 eq; 70 wt. % in H_2O) was added and the solution was stirred for an additional 15 min. The reaction mixture was diluted with toluene (15 mL) and poured into a separating funnel containing saturated sodium hydrogen carbonate solution (10 mL). The organic phase was washed with brine, dried with $MgSO_4$ and the volatile components were removed under reduced pressure. The resulting material was then dissolved in DCM and purified by flash chromatography on silica gel using ethyl acetate/n-hexane 7:3 as eluent. The fractions containing intermediate **α-4Ac-Man-P-Cit-BZP-yne** (R_f = 0.33; on TLC, same solvent system as for the column) were combined and the solvents were removed on a rotary evaporator. To the white residue, an ammonia solution (4 mL; 2.0 M in methanol) was added and the reaction mixture was stirred overnight at rt. Afterwards, the volatile components were removed under reduced pressure, the residue was dissolved in methanol (2 mL) and the material was purified by PLC (using chloroform/methanol/water 60:25:4 as the mobile phase). The broad band, centered at around an Rf ≈ 0.19, was removed and the product was extracted with methanol (100 mL). The solvent was removed on a rotary evaporator to give the product **MPC-3** (65 mg = 0.10 mmol, 24% yield) as a white solid.

^{1}H NMR (DMSO-d_6, 400 MHz): δ (ppm) 7.75 – 7.64 (m, 4H), 7.16 – 7.04 (m, 4H), 5.58 (t, J=7.2 Hz, 1H), 5.17 (dd, J=7.8, 1.9 Hz, 1H), 4.91 (d, J=2.4 Hz, 2H), 4.88 – 4.87 (m, 3H), 4.76 – 4.71 (m, 2H), 4.50 (s, 2H), 3.77 – 3.63 (m, 2H), 3.63 – 3.51 (m, 4H), 3.42 – 3.23 (m, 2H), 2.04 (m, 2H), 1.69 (s, 3H), 1.60 – 1.45 (m, 2H), 1.41 – 1.24 (m, 2H), 1.24 – 1.10 (m, 1H), 0.85 (d, J=6.4 Hz, 3H).

^{13}C NMR (DMSO-d_6, 101 MHz): δ (ppm) 193.74, 162.47, 160.84, 132.31, 132.15, 131.22, 130.61, 130.26, 129.71, 115.07, 115.03, 96.05, 95.99, 79.24, 79.14, 74.51, 74.06, 71.46, 71.38, 70.92, 67.64, 62.81, 62.76, 61.80, 56.17, 37.90, 37.83, 36.75, 29.30, 25.11, 19.68, 14.16.

^{31}P NMR (DMSO-d_6, 121 MHz): δ (ppm) -2.86.

HR-MS (NSI): calc. m/z for $[C_{32}H_{41}O_{12}P - H^+]^-$ = 647.23, found m/z = 647.2274.

Analytical data NMR

Compound ***THP-Cit-BZP-yne***

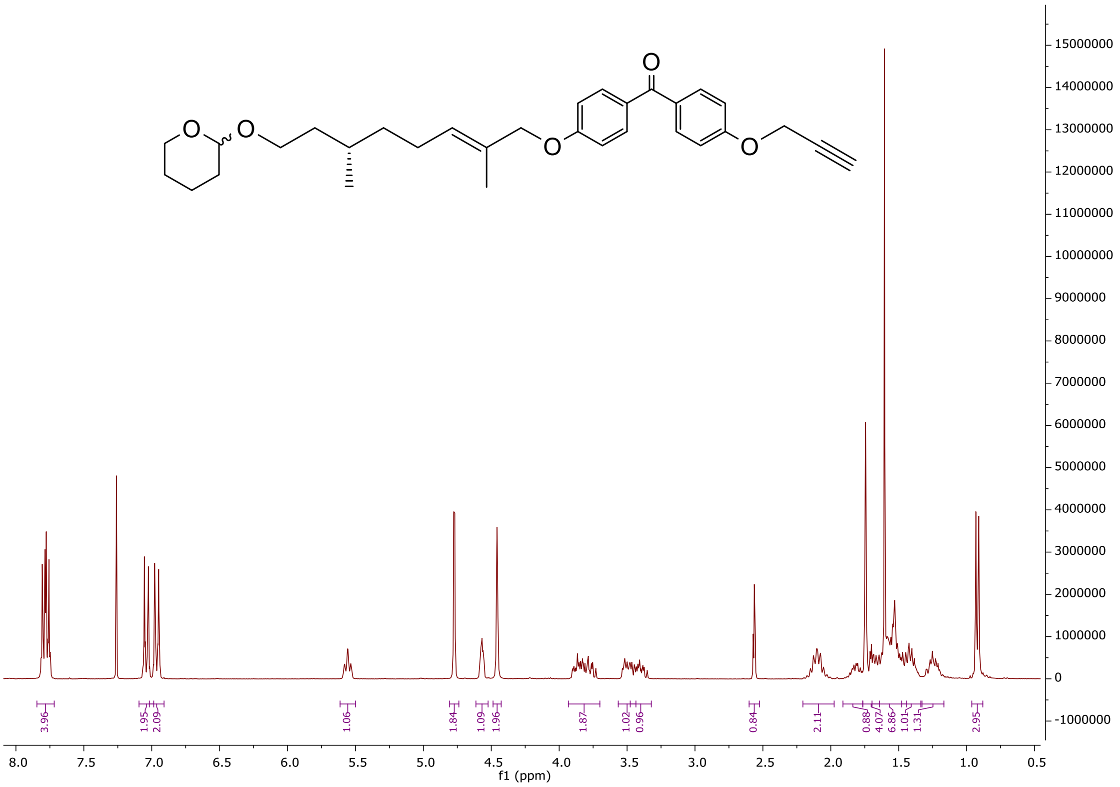

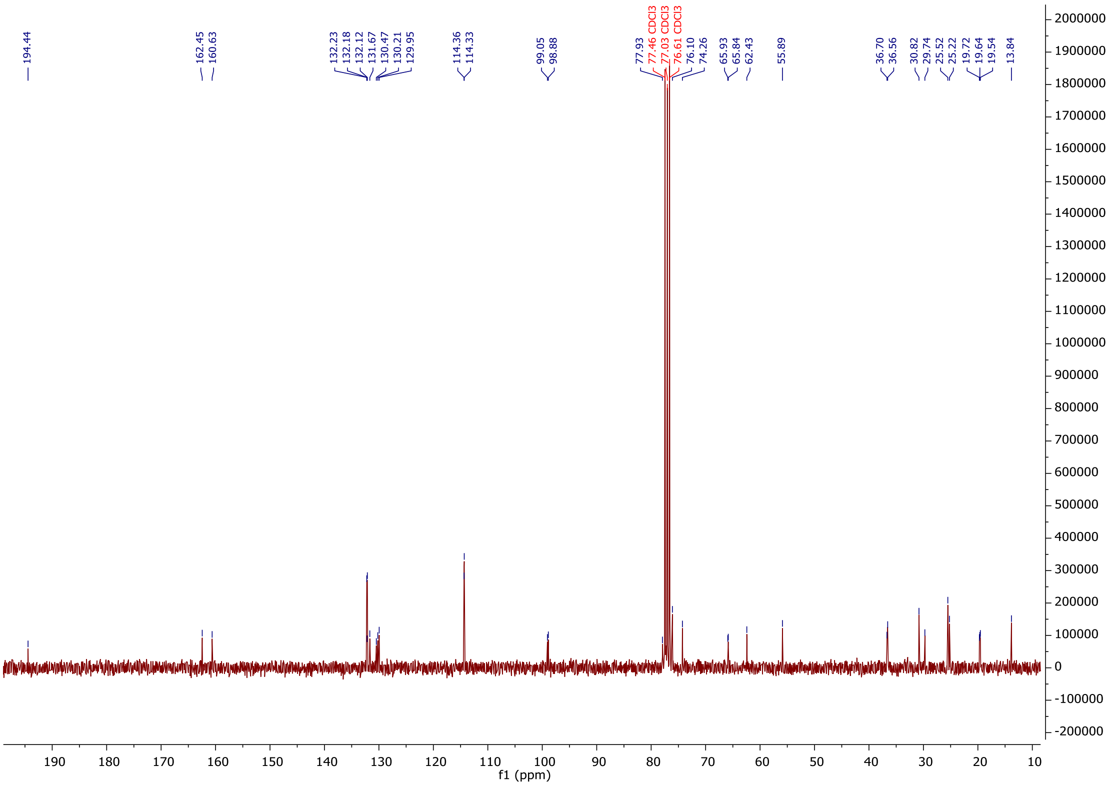

Compound ***Cit-BZP-yne***

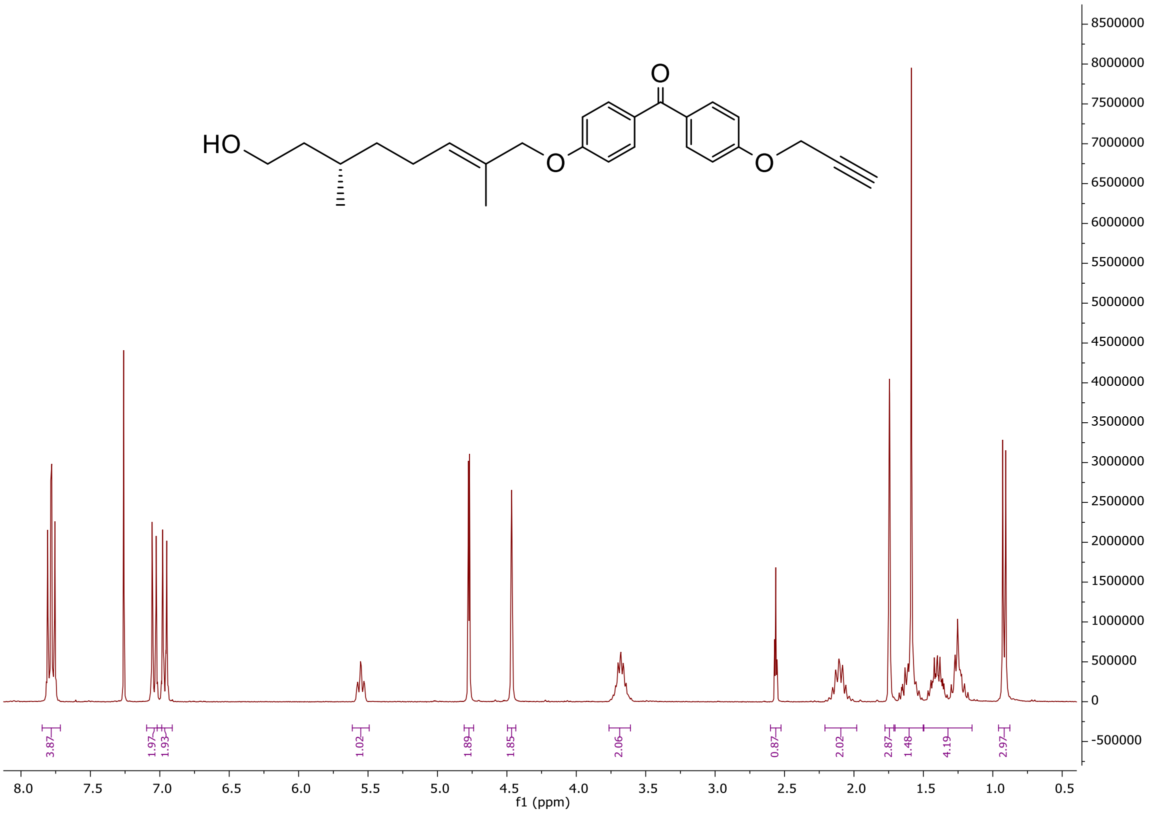

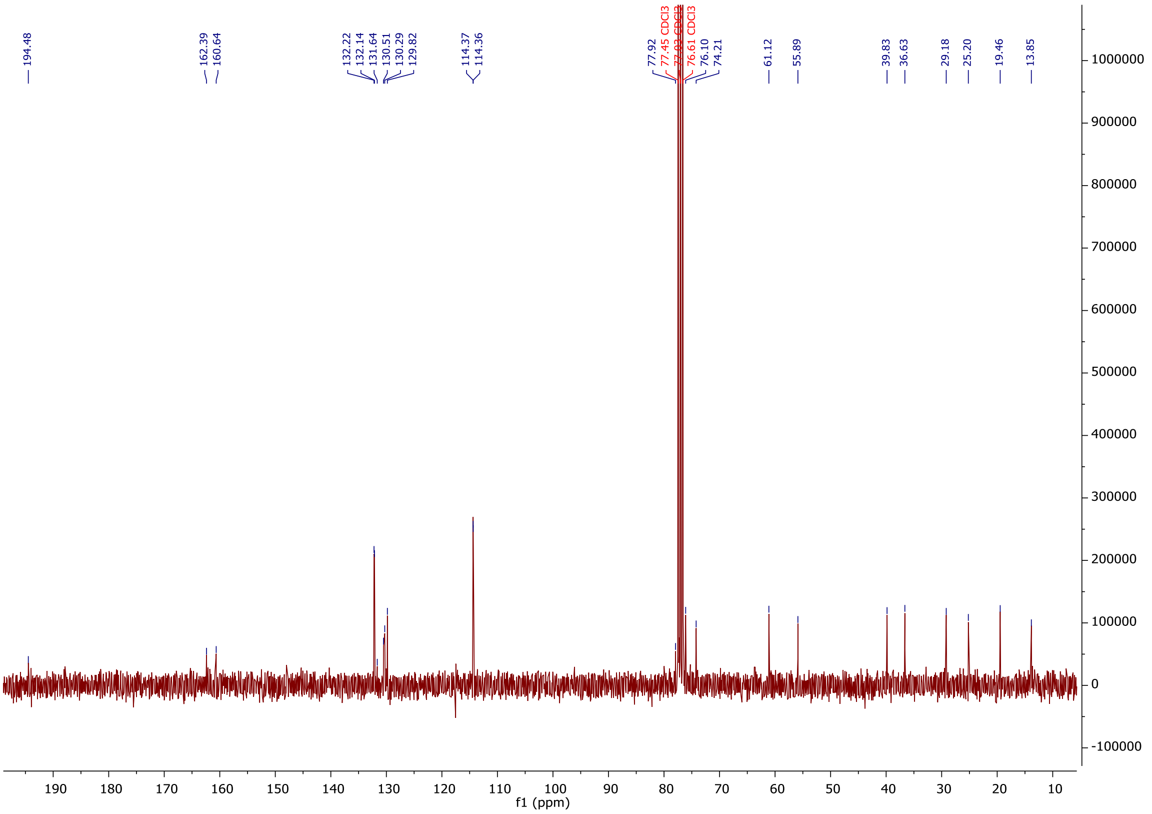

Compound ***β-4Ac-Man-CEP***

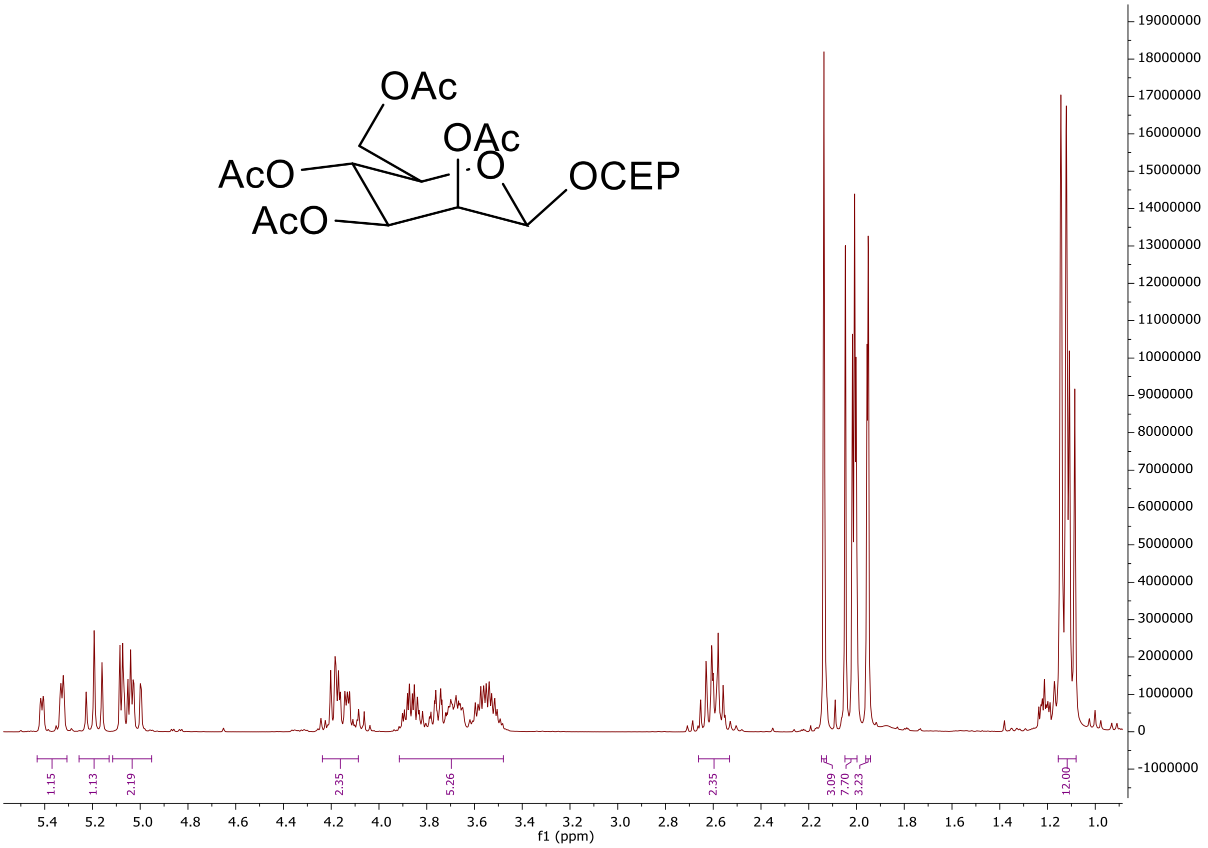

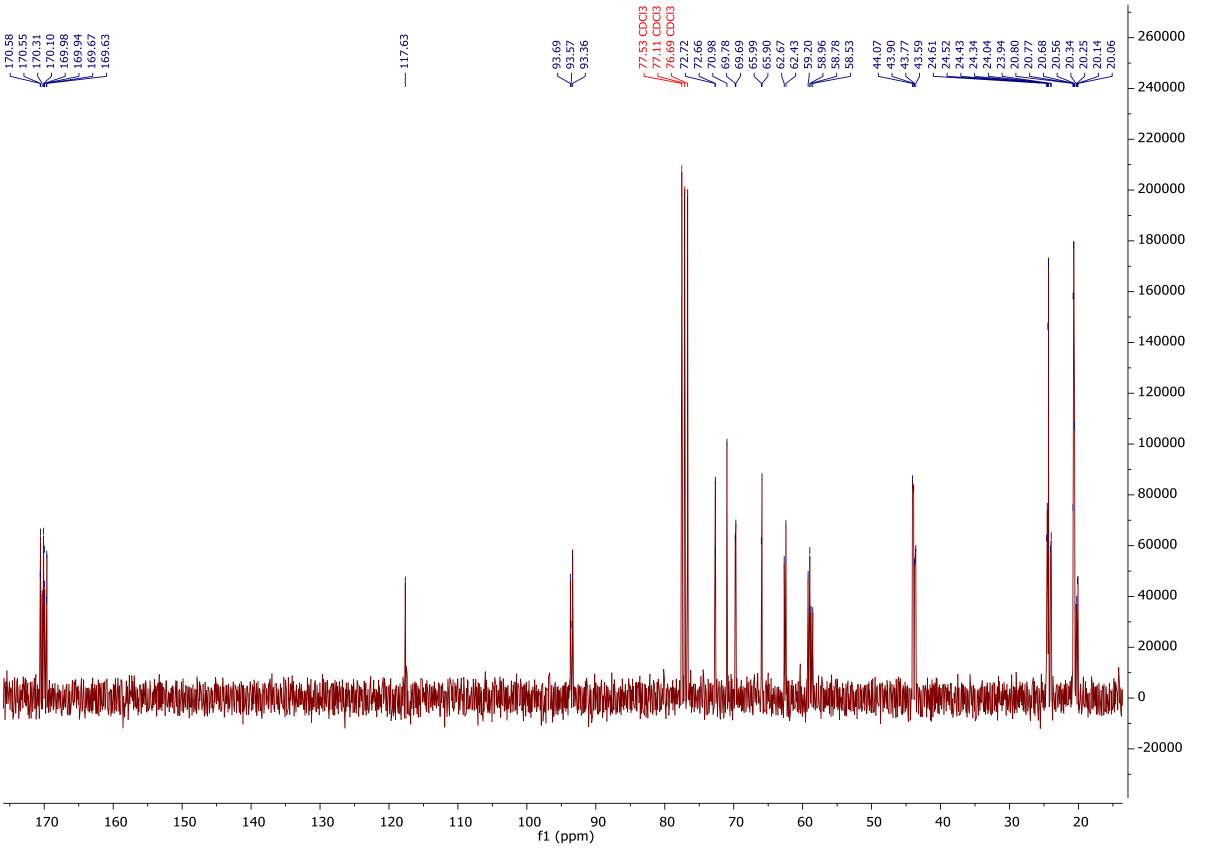

*Compound **β-4Ac-Man-CEP***

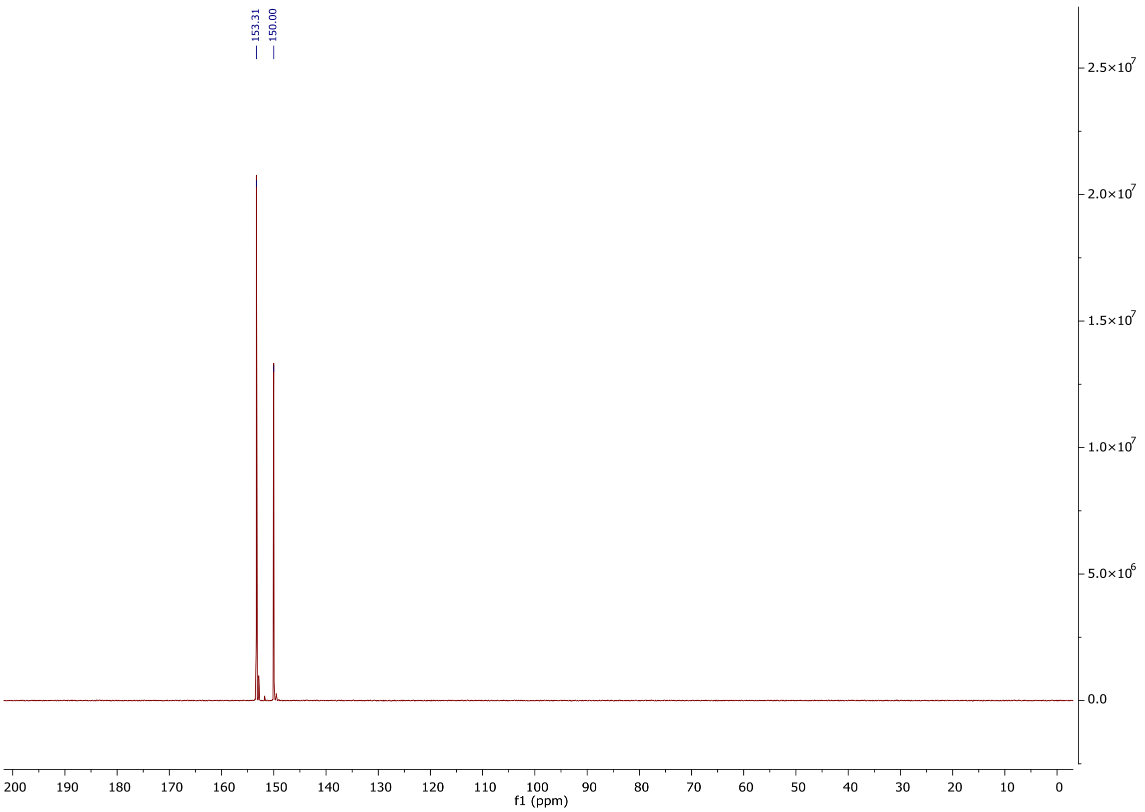

*Compound **MPC-1***

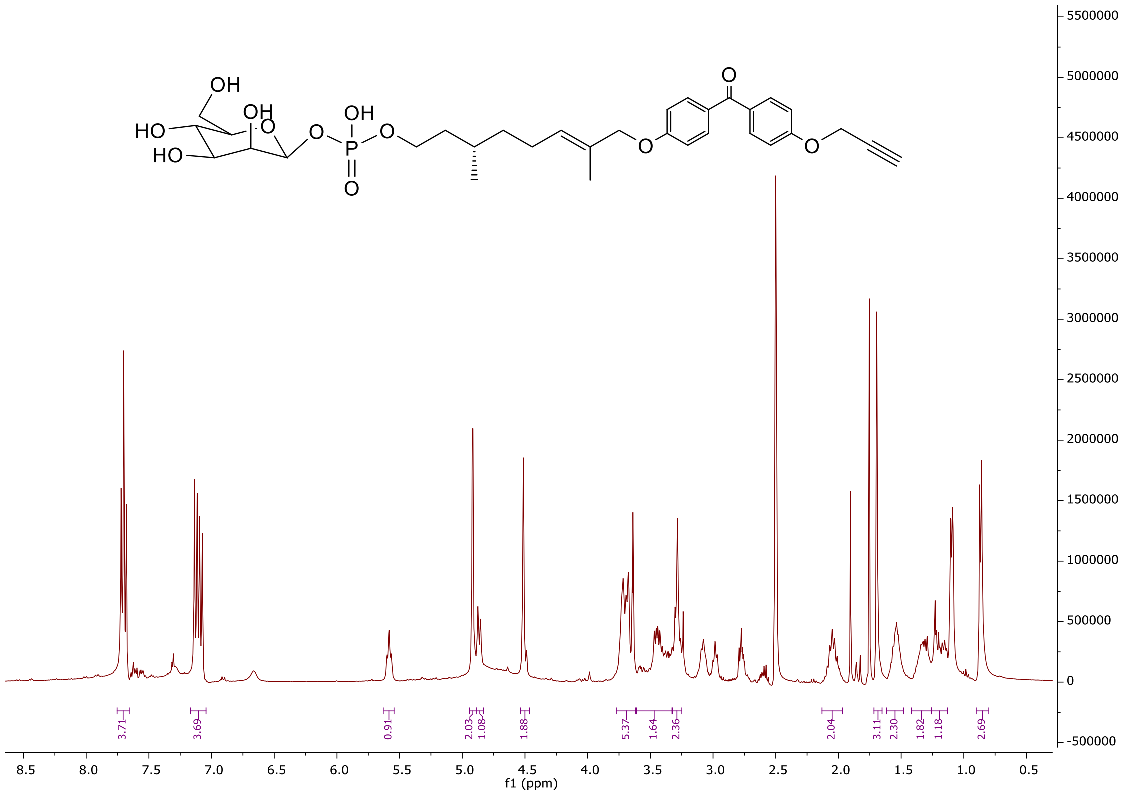

Compound ***MPC-1***

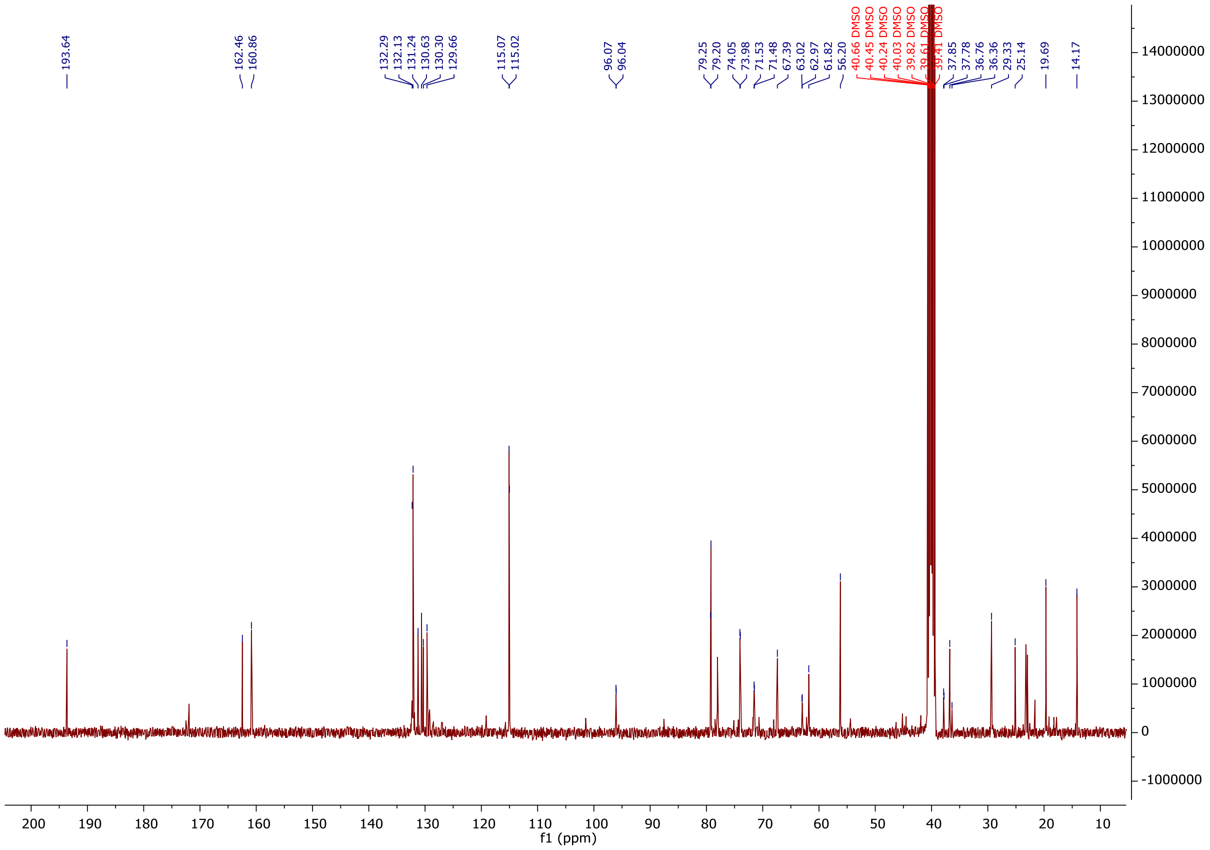

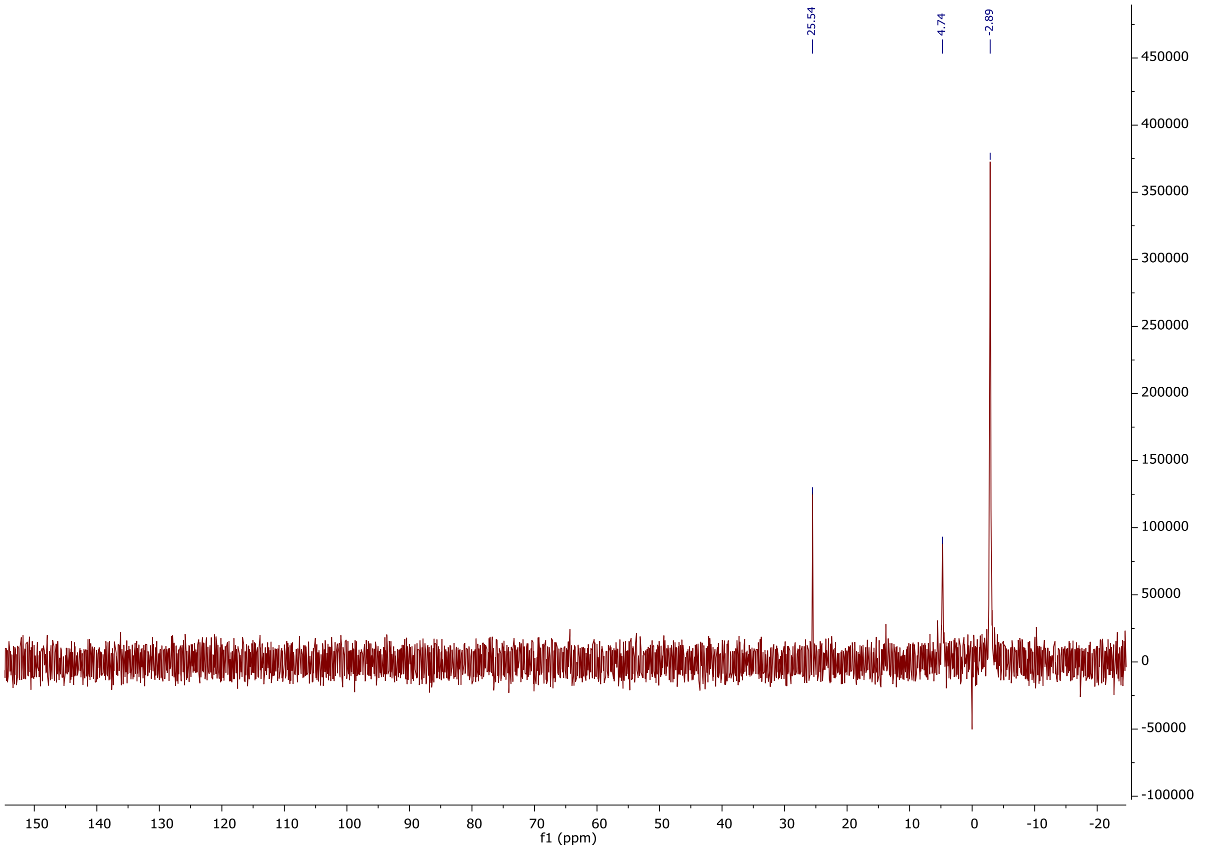

Compound ***MPC-1***

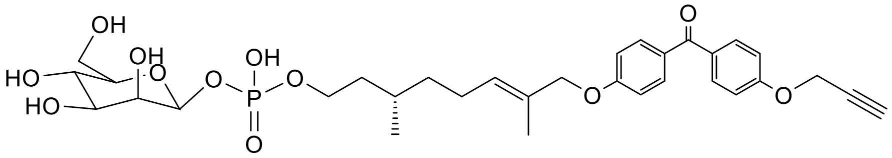

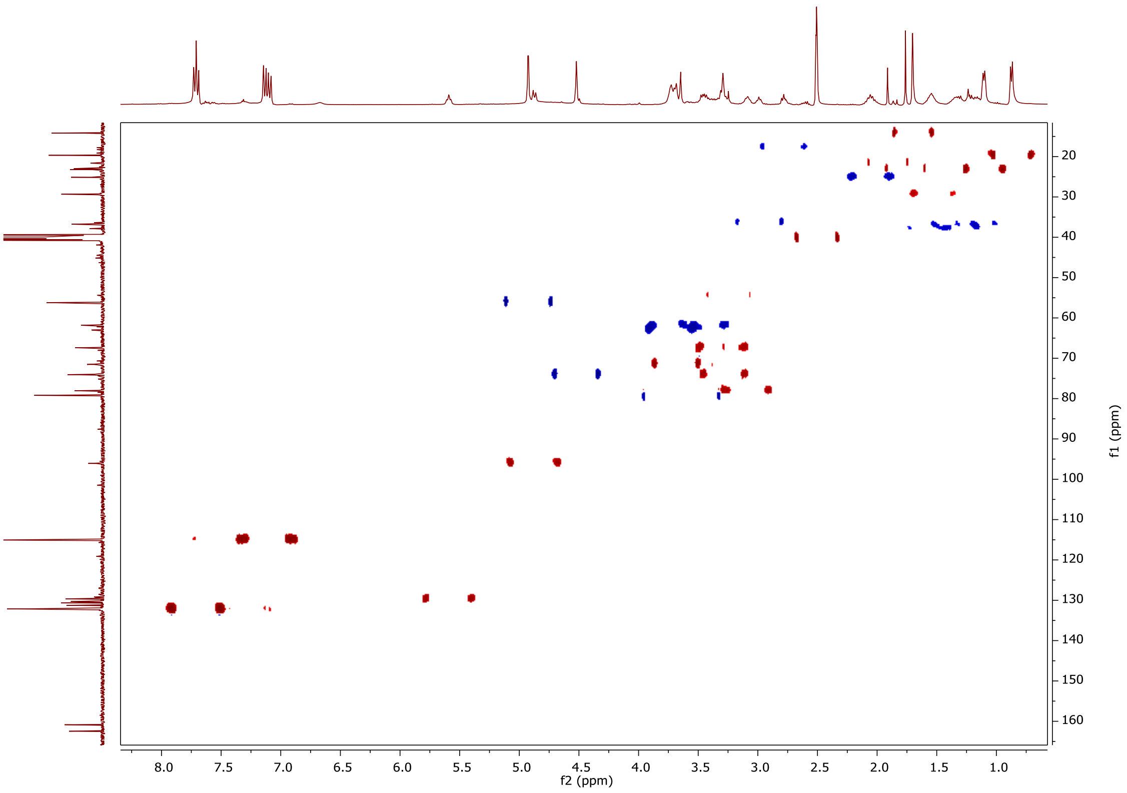

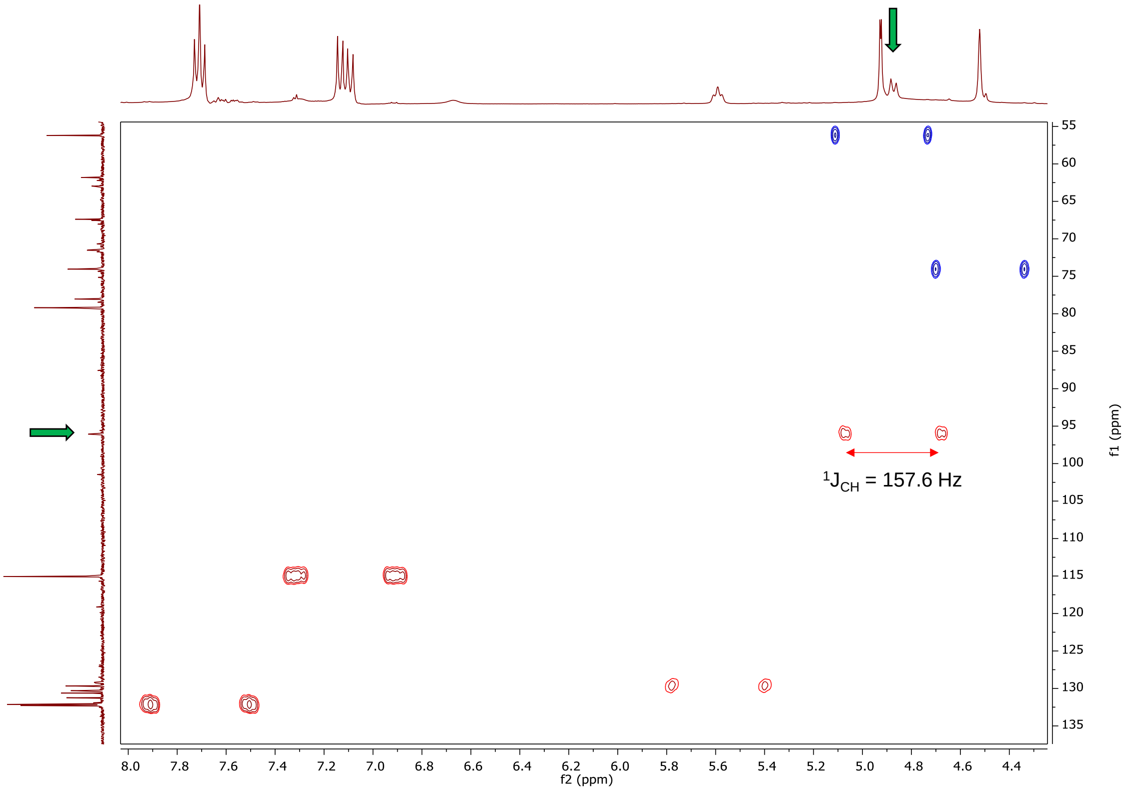

Compound ***DMT-Cit***

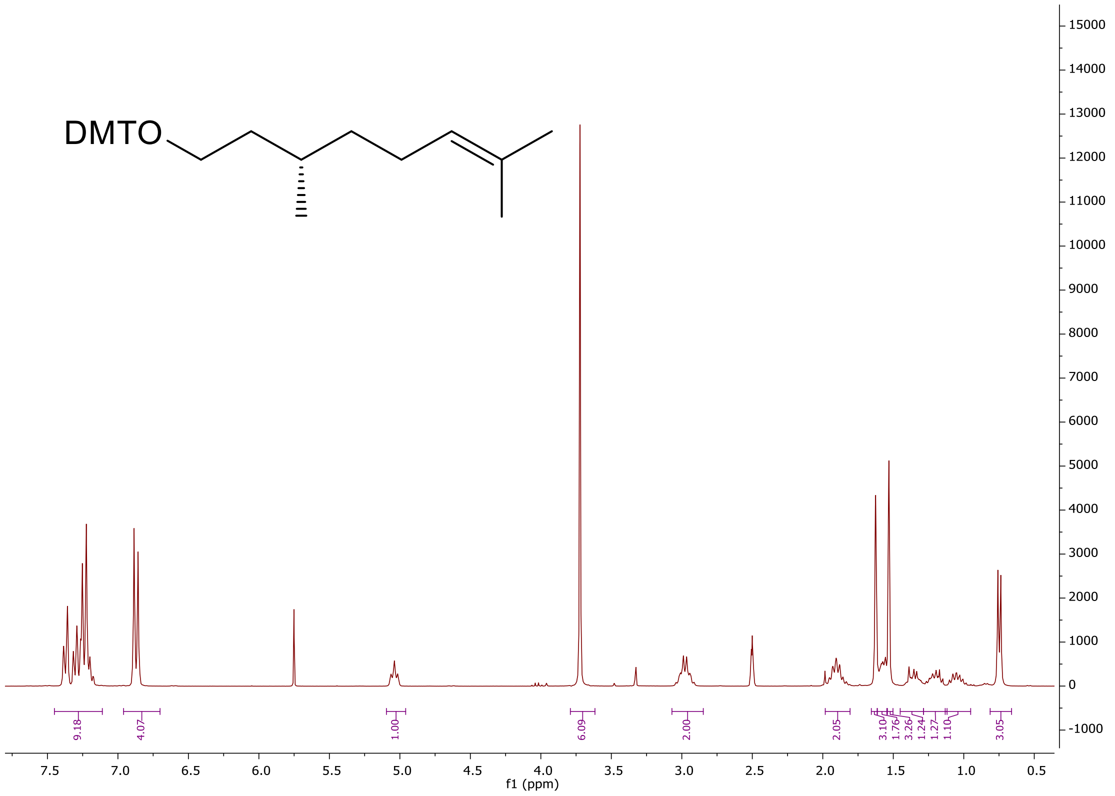

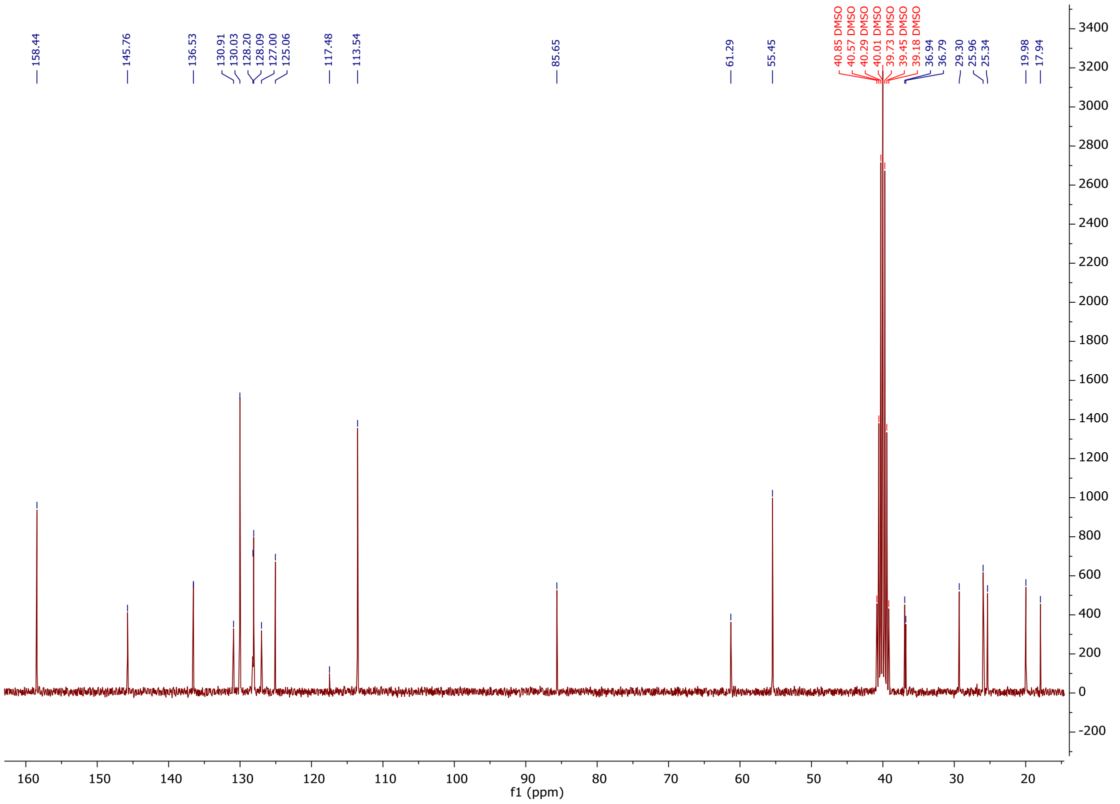

Compound ***DMT-Cit-OH***

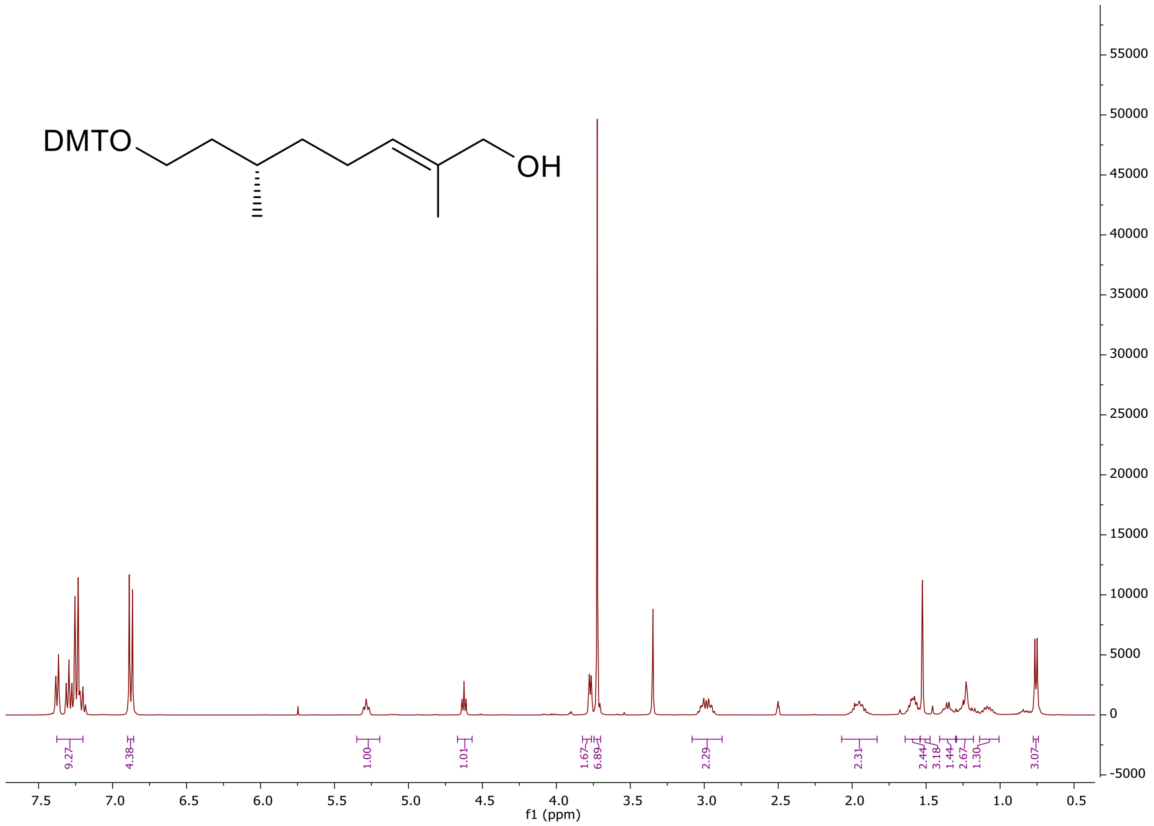

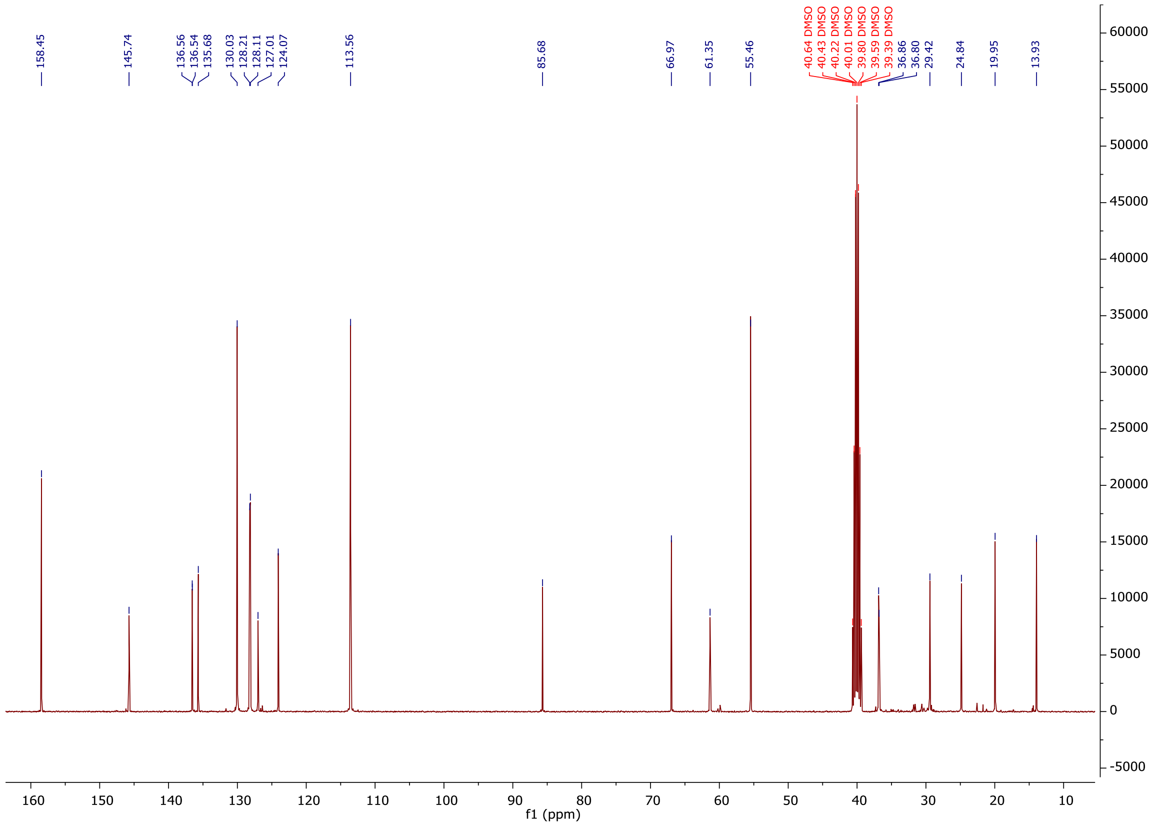

Compound ***DMT-Cit-Dod-TBDMS***

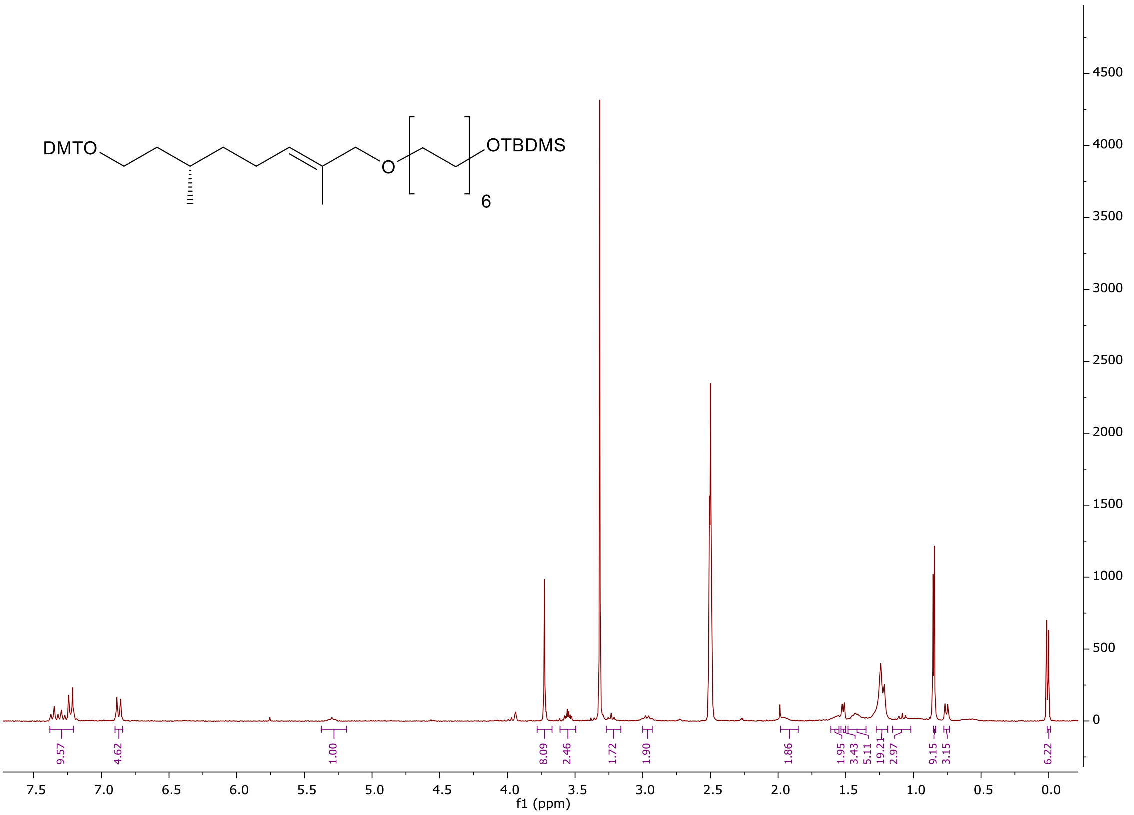

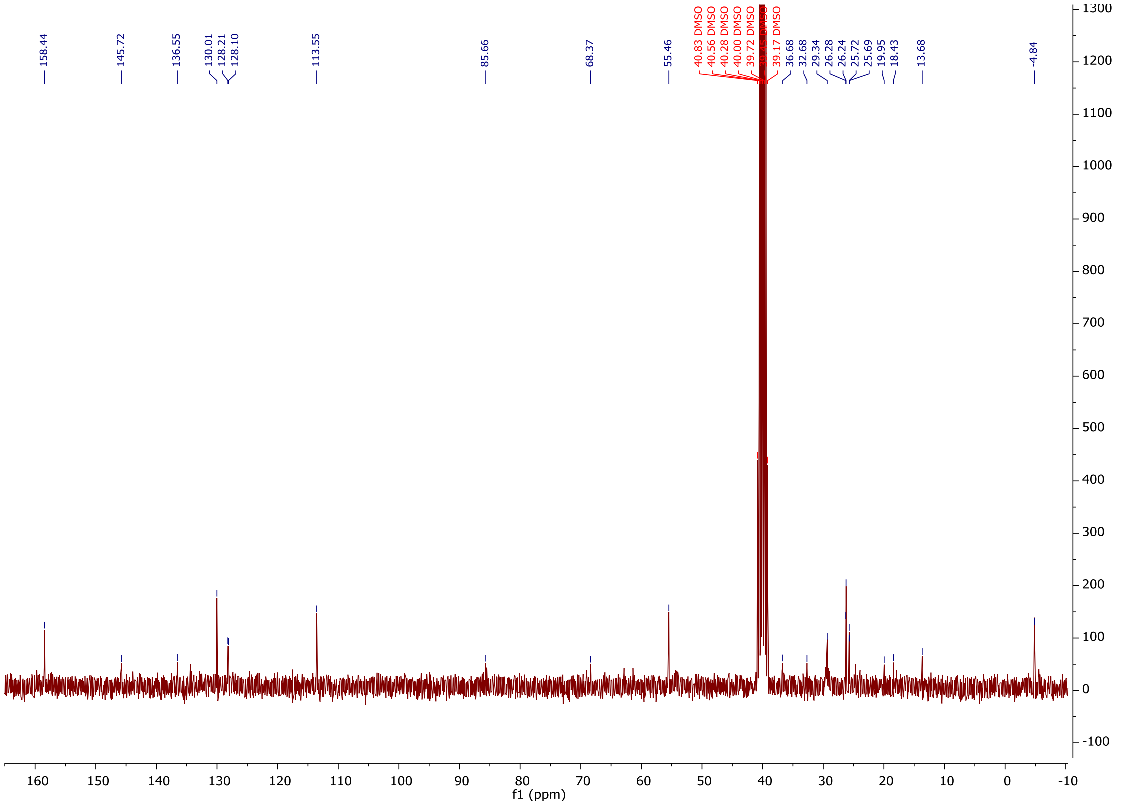

Compound ***DMT-Cit-Dod***

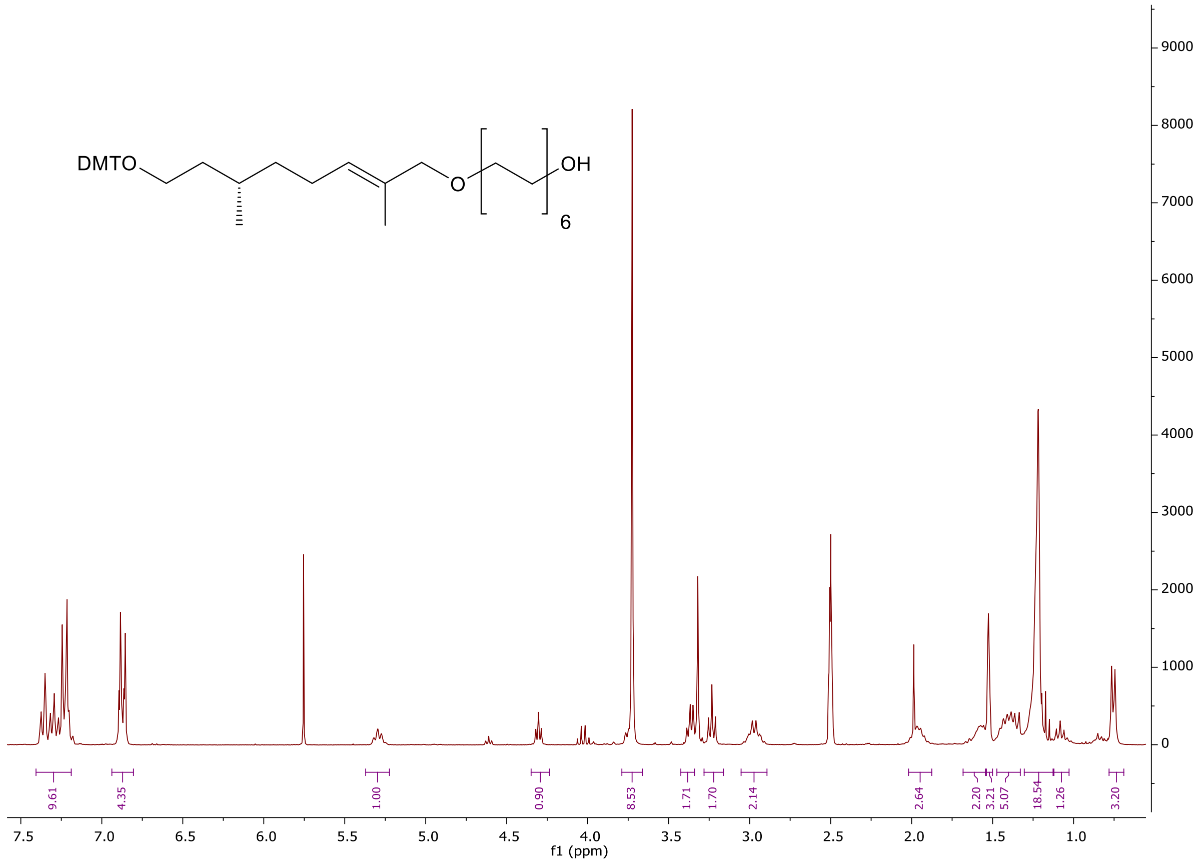

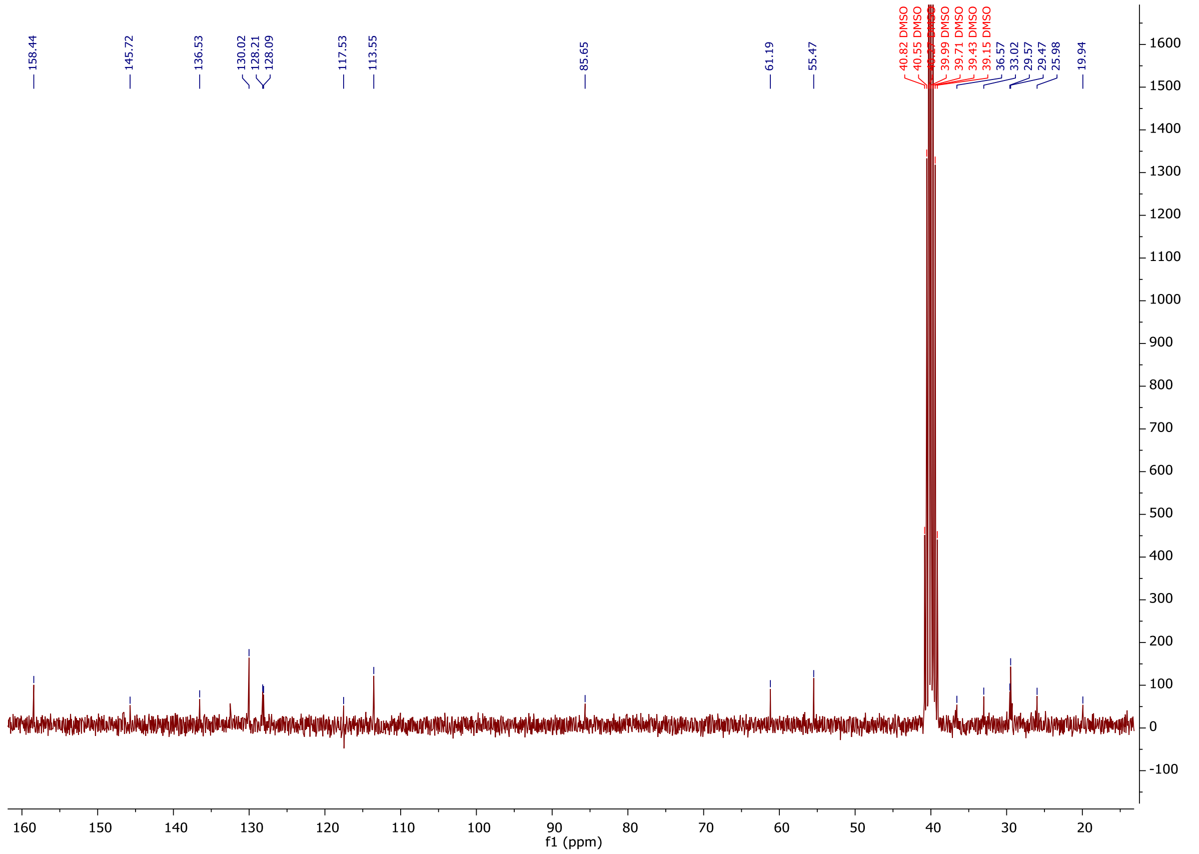

Compound ***DMT-Cit-Dod-Pht***

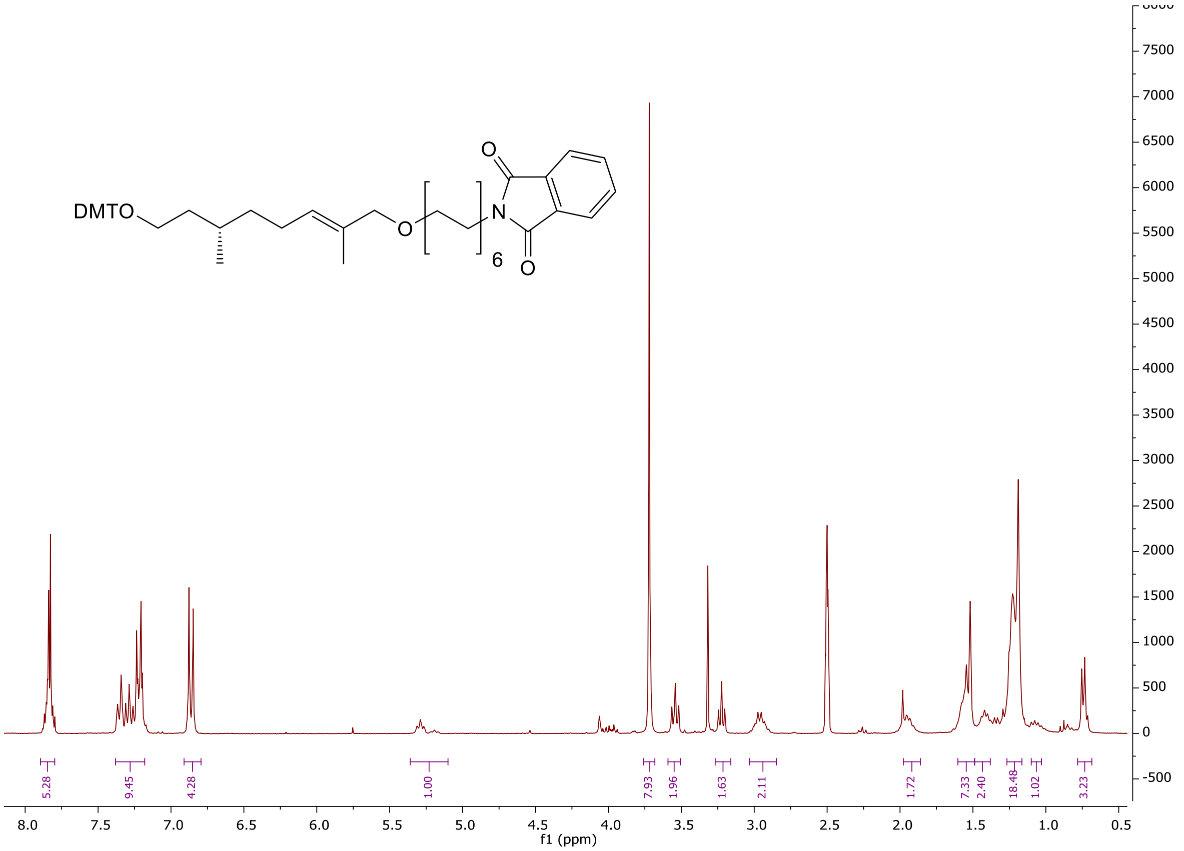

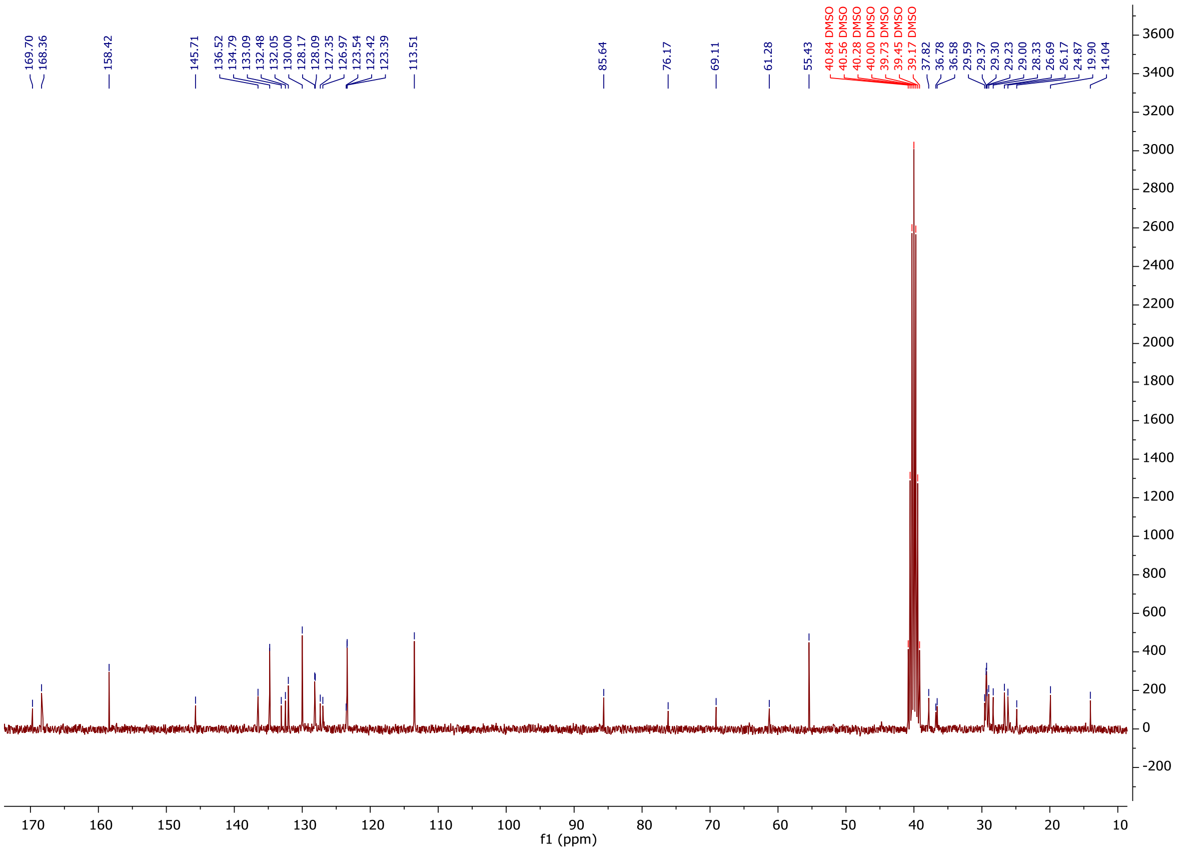

Compound ***DMT-Cit-Dod-NH2***

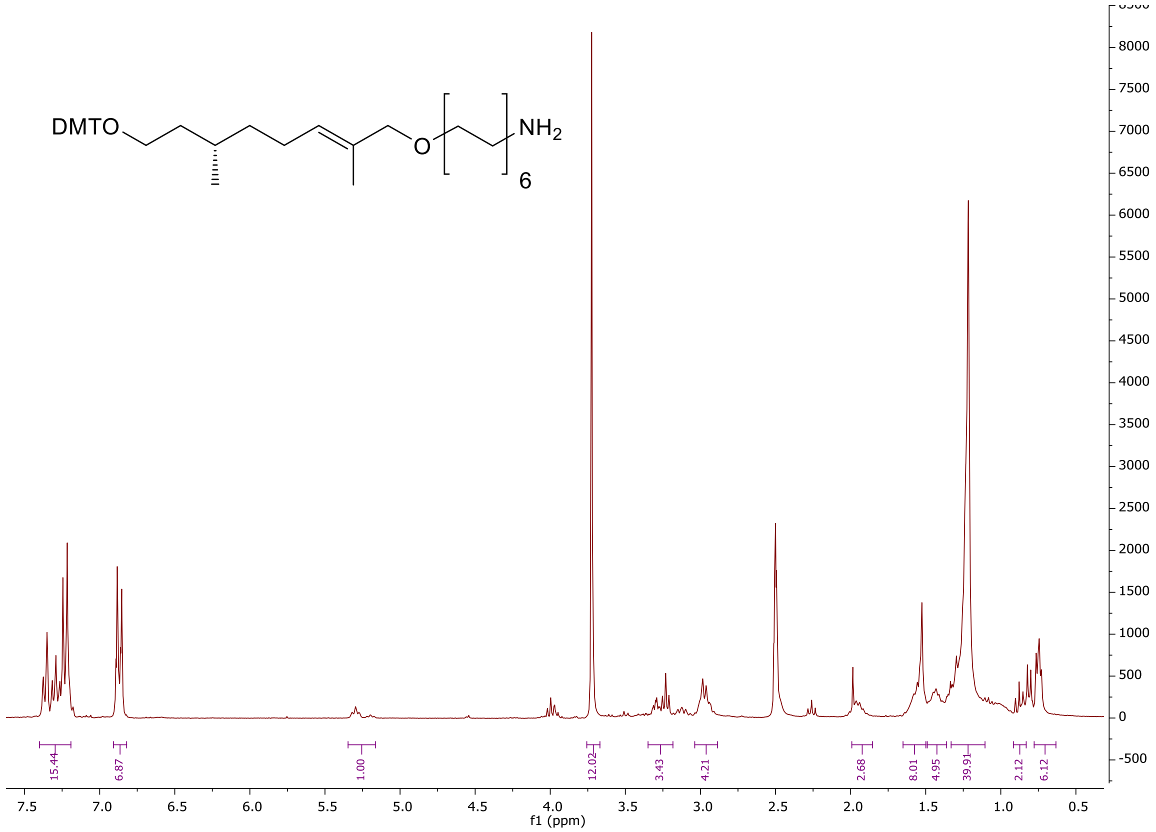

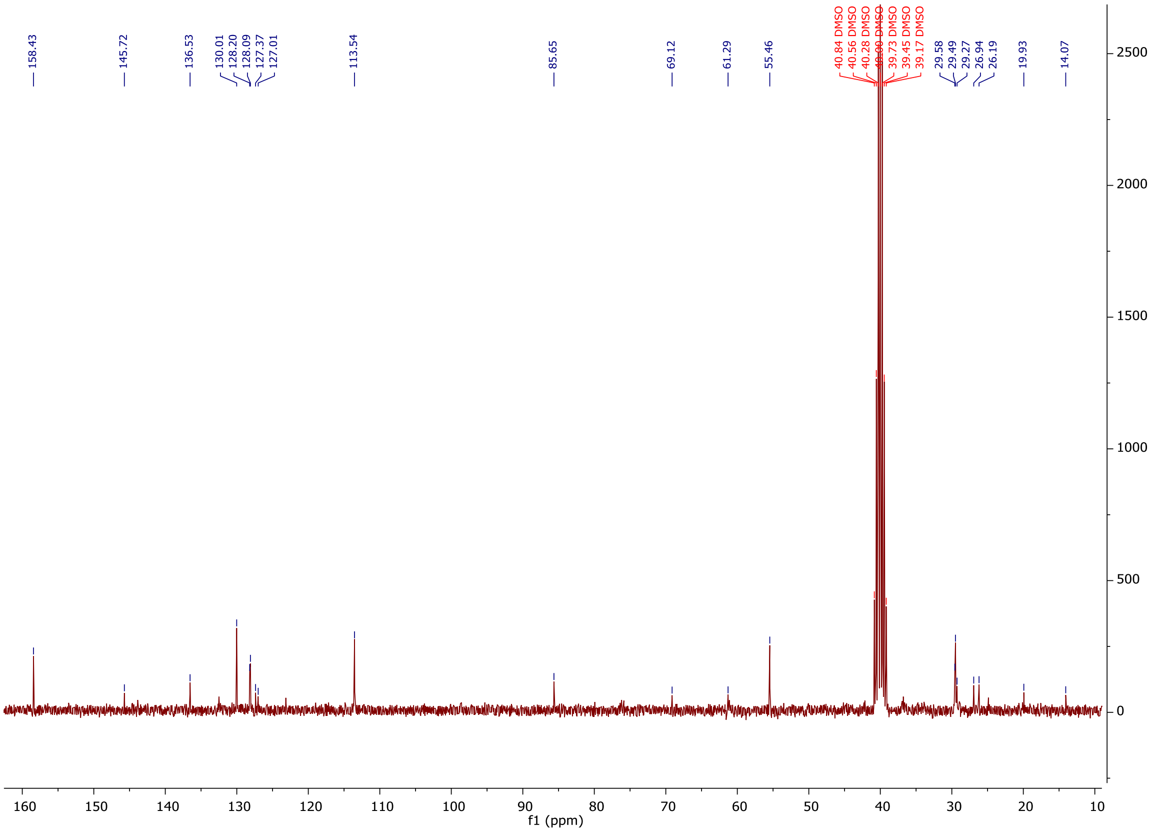

Compound ***DMT-Cit-Dod-NBD***

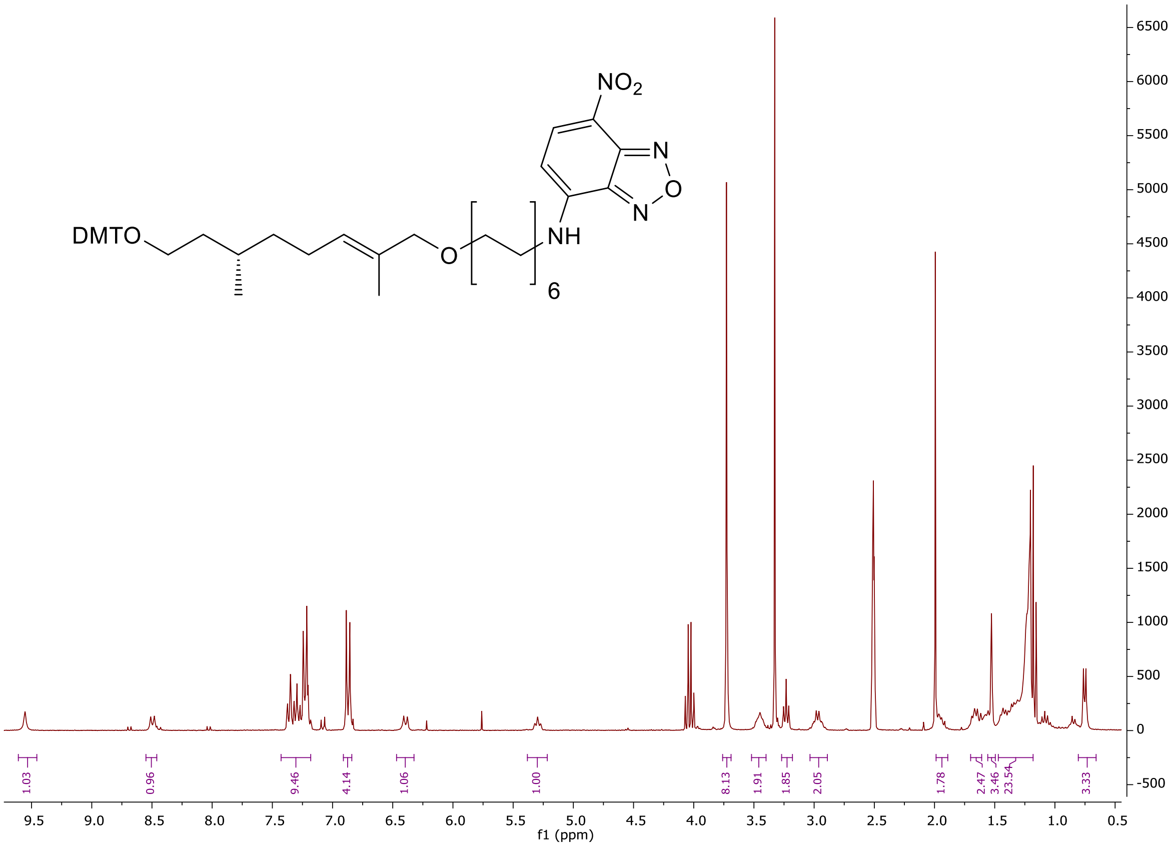

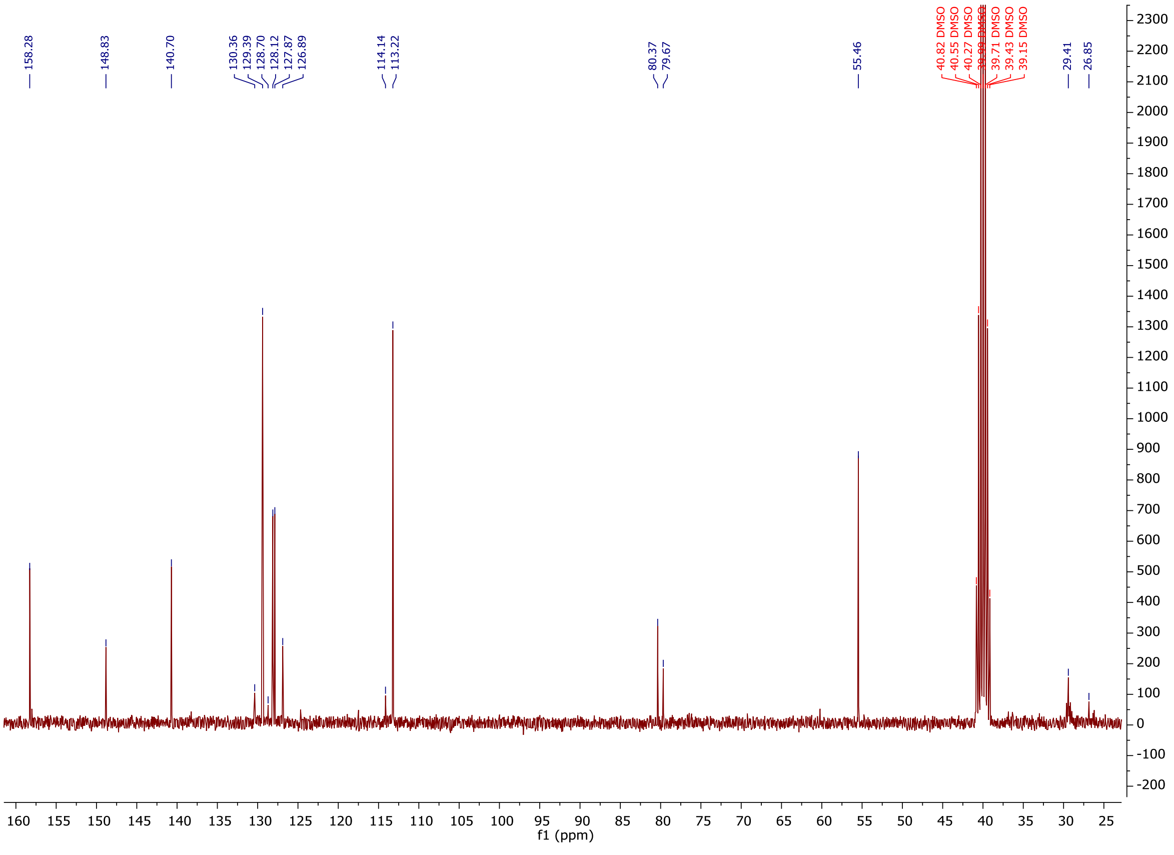

Compound ***Cit-Dod-NBD***

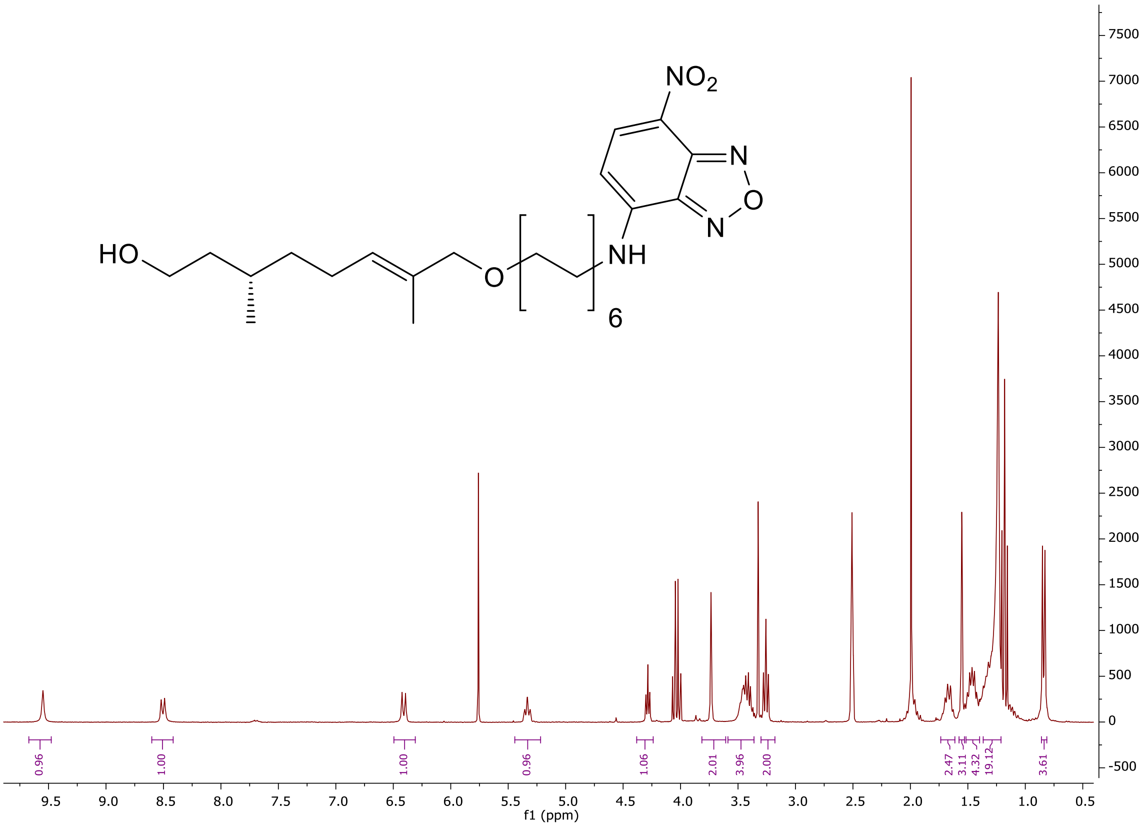

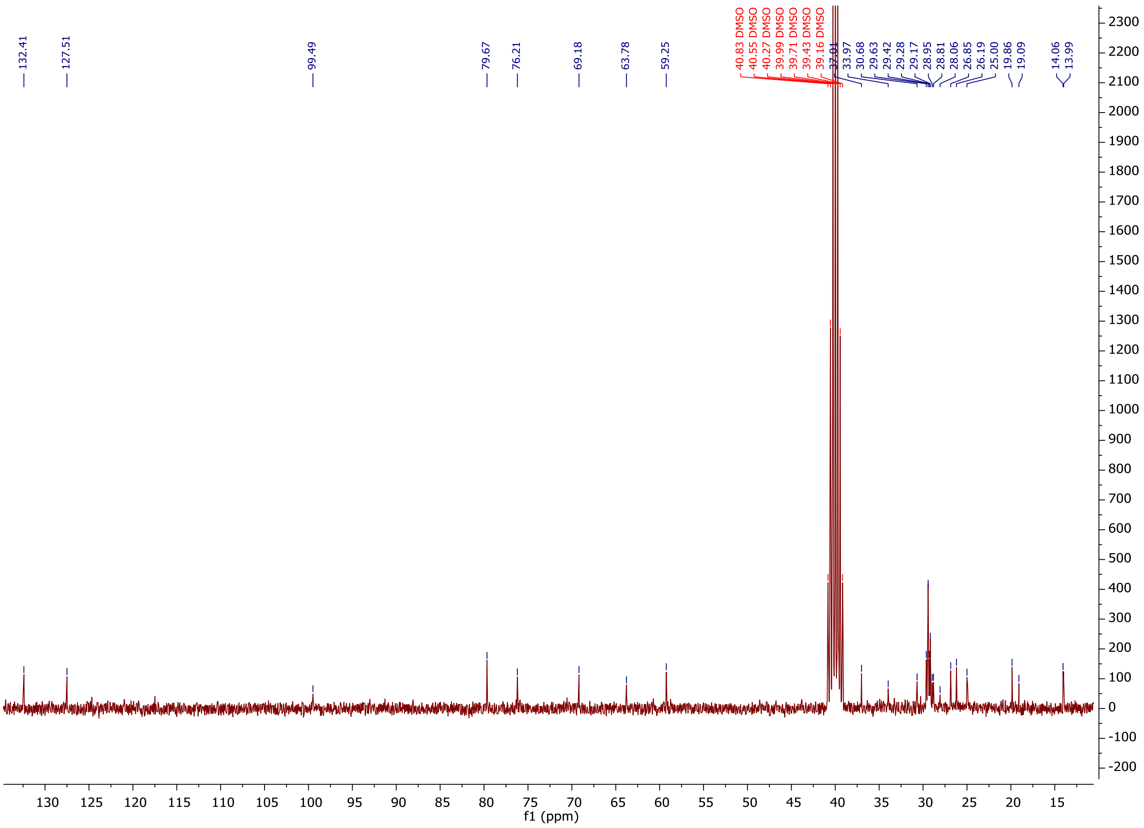

Compound ***MPC-2***

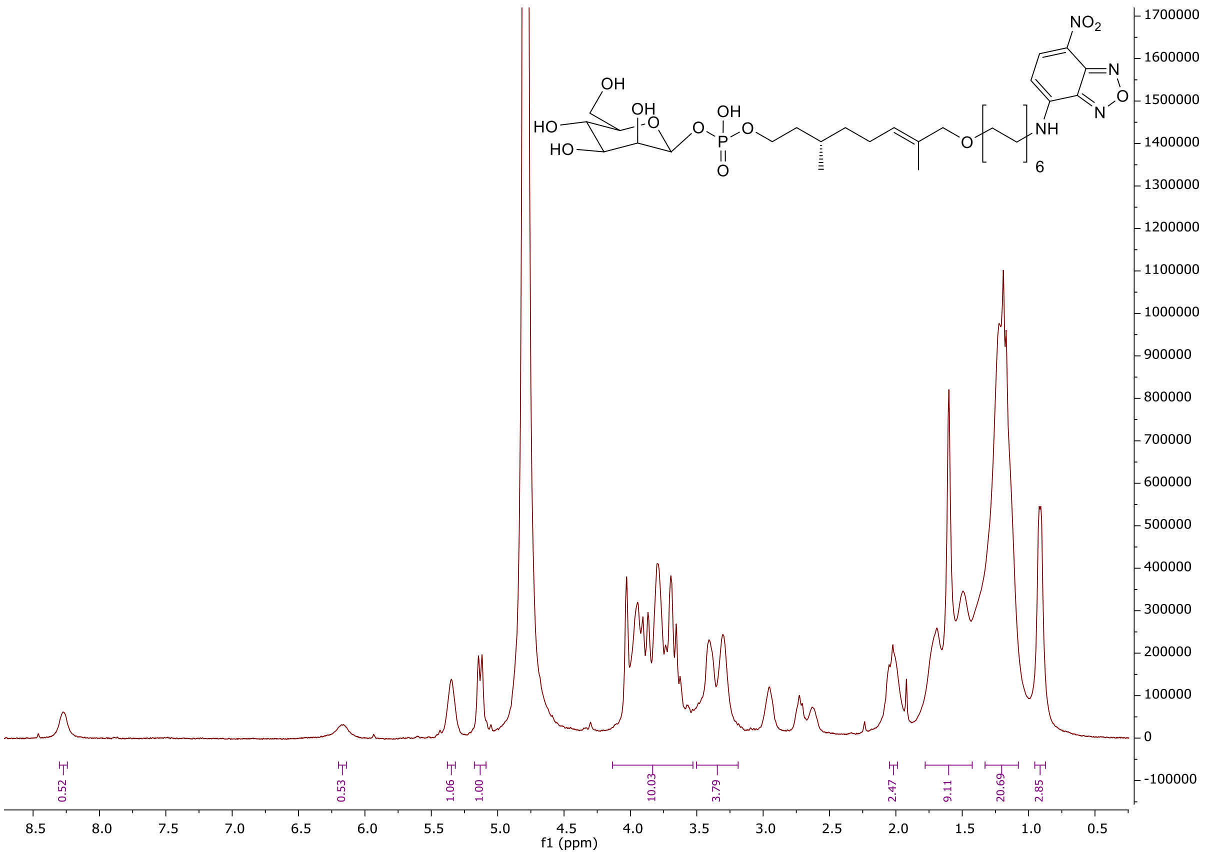

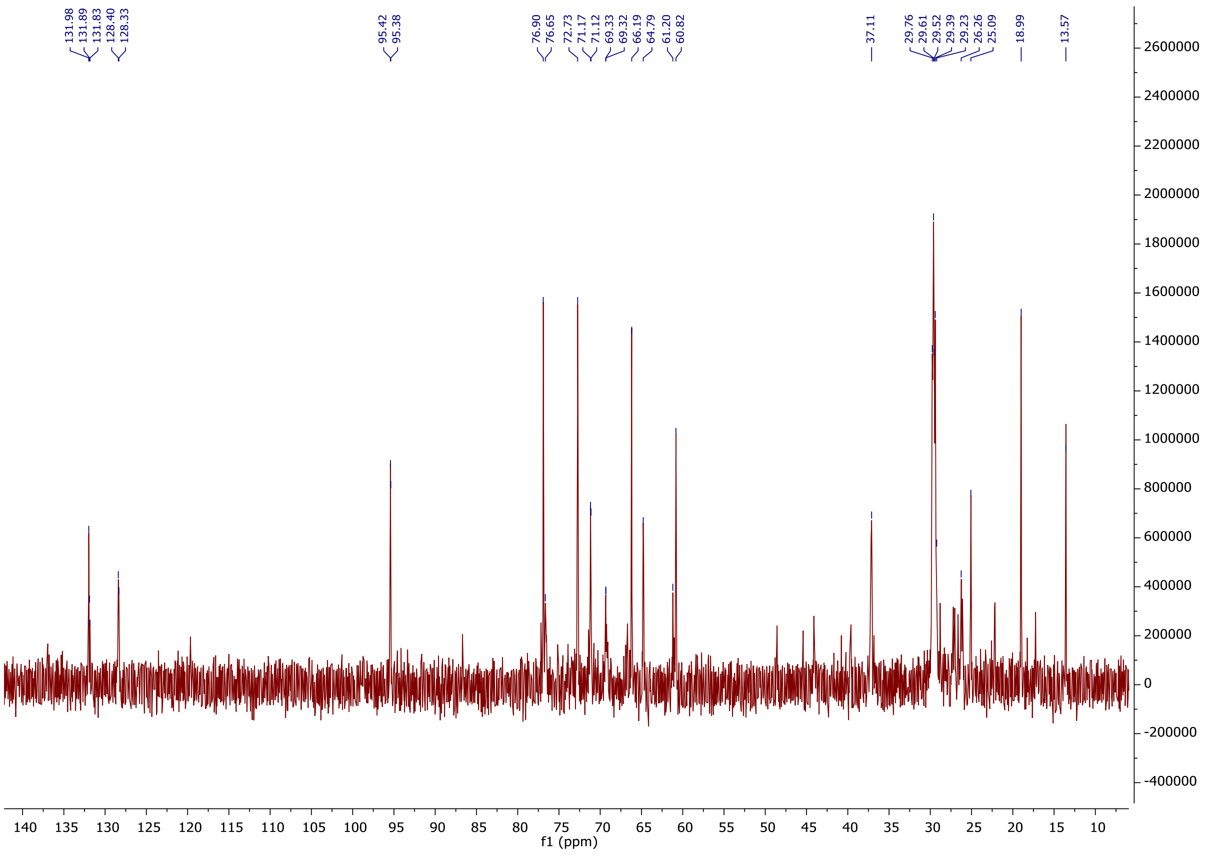

Compound ***MPC-2***

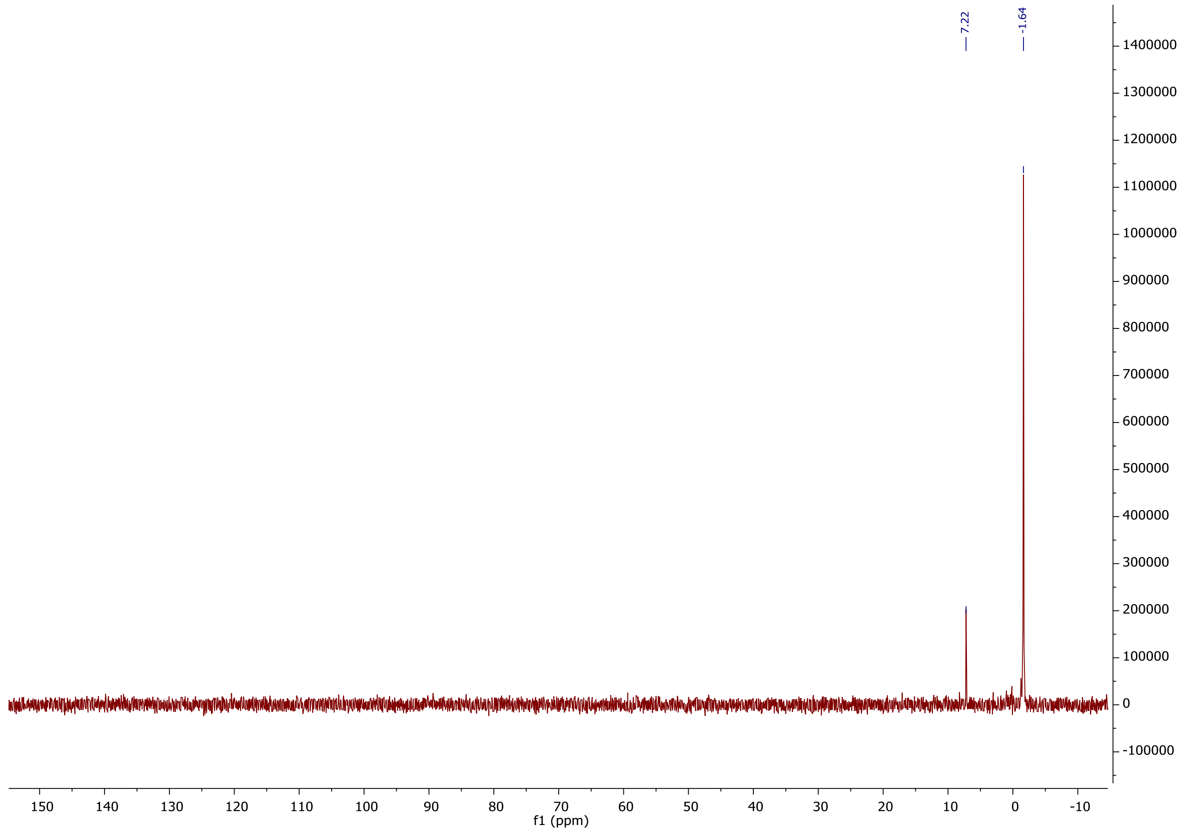

*Compound **MPC-2***

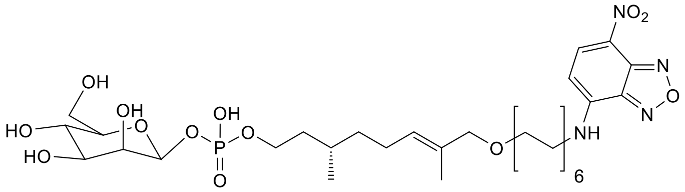

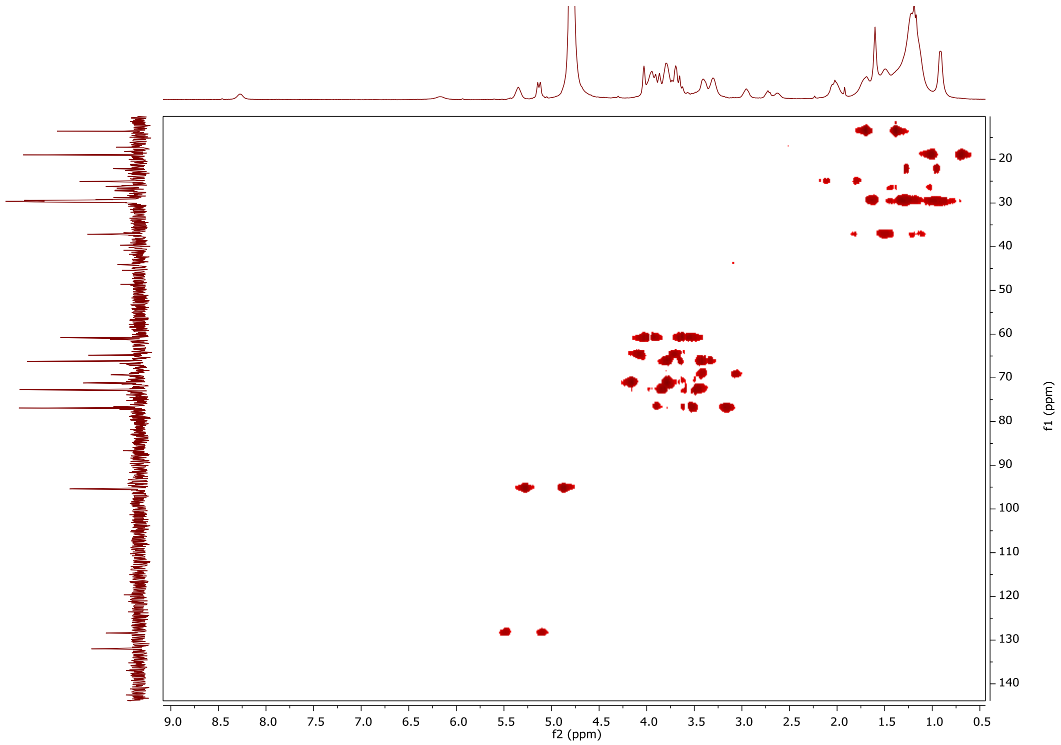

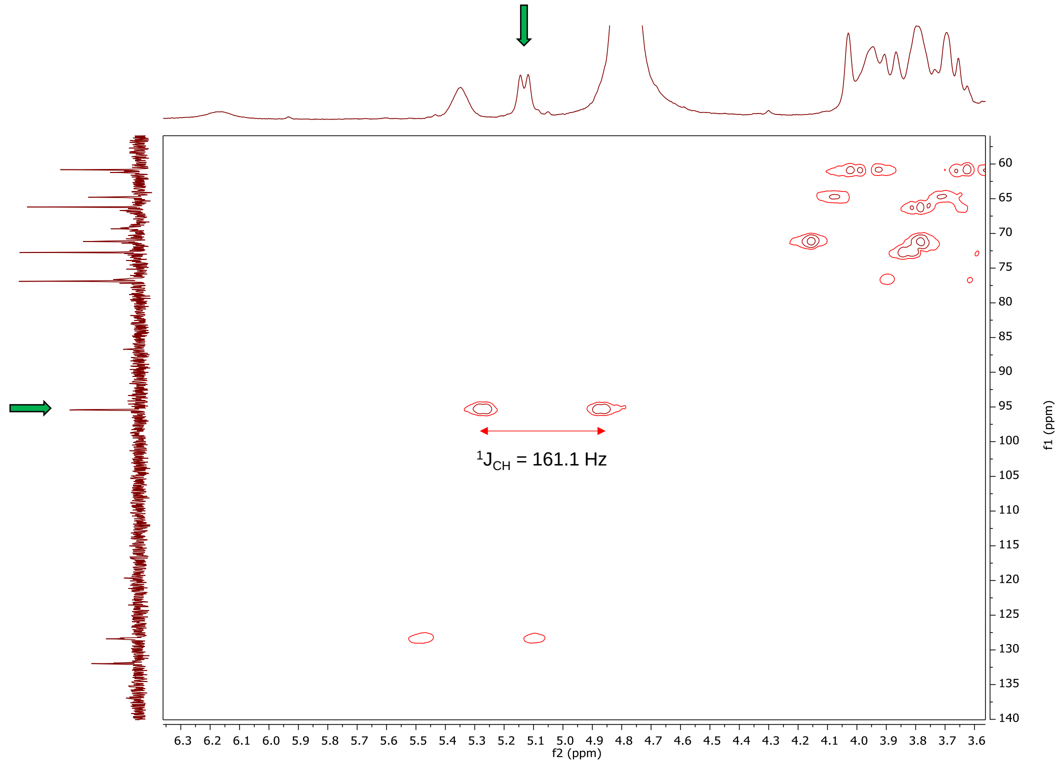

Compound ***α-4Ac-Man-CEP***

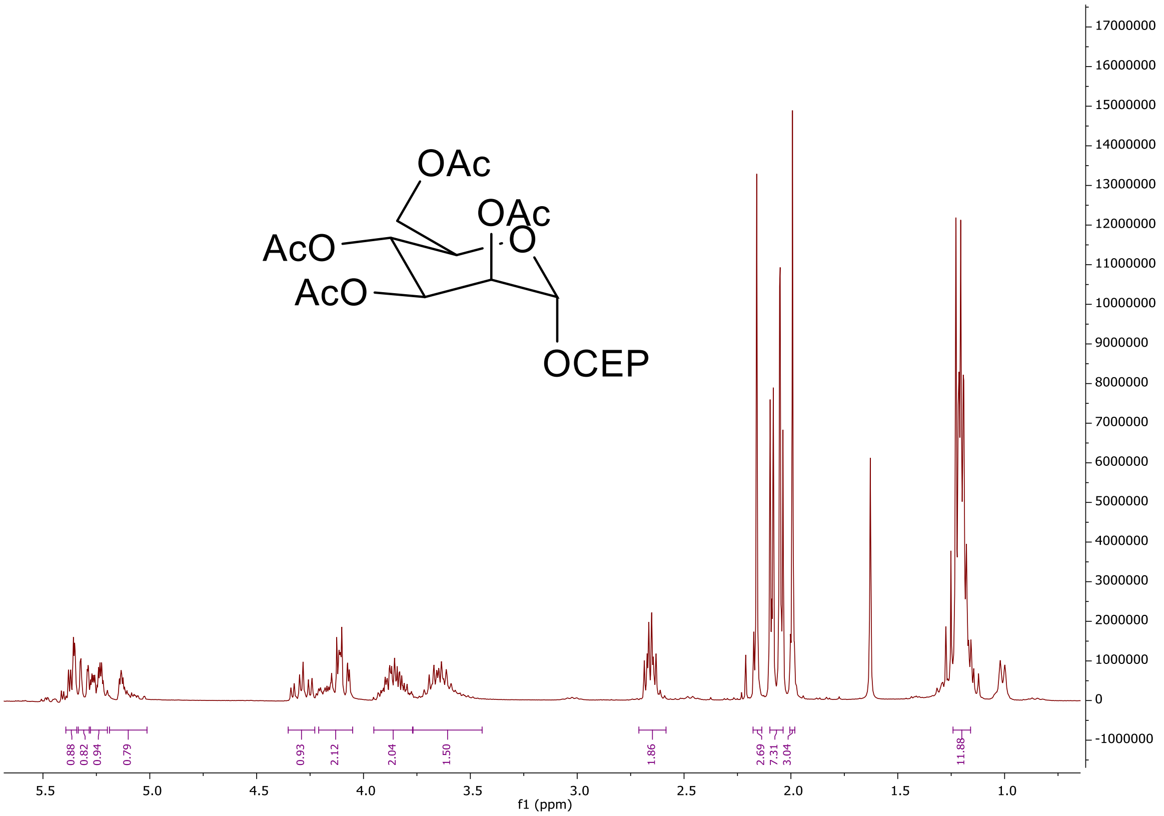

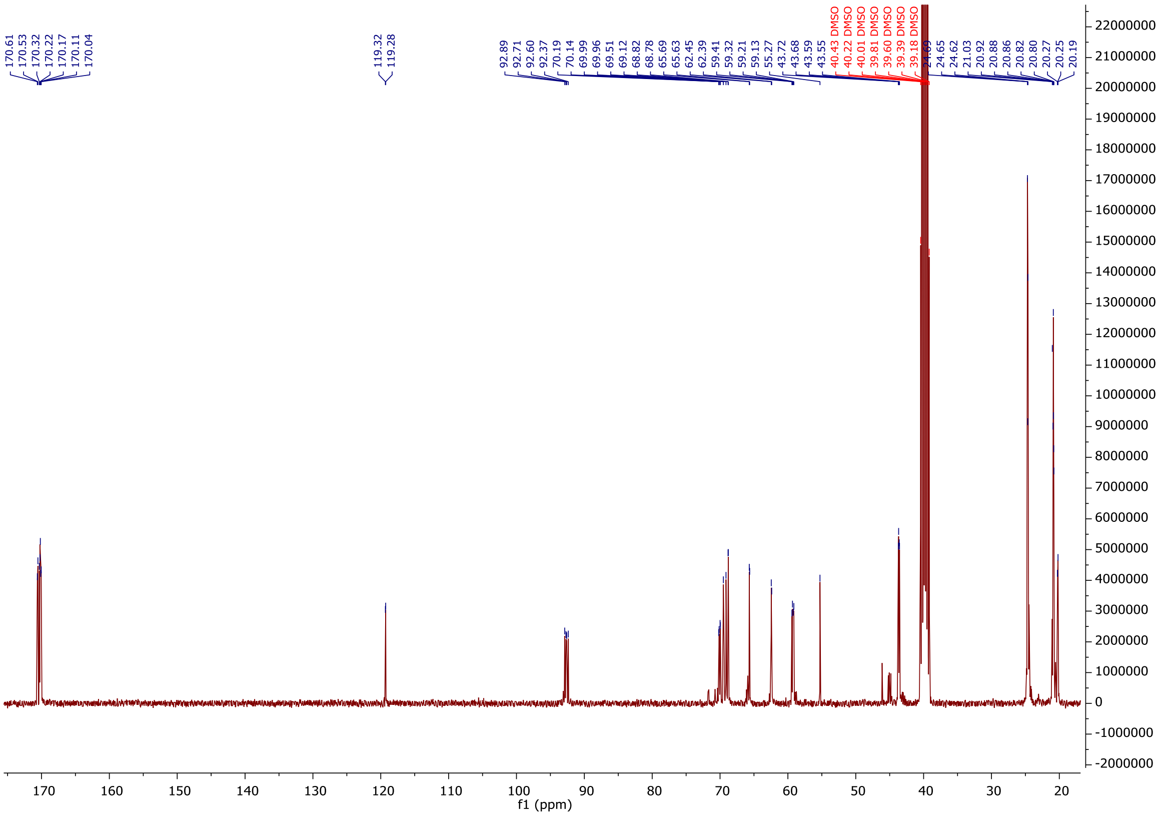

Compound ***α-4Ac-Man-CEP***

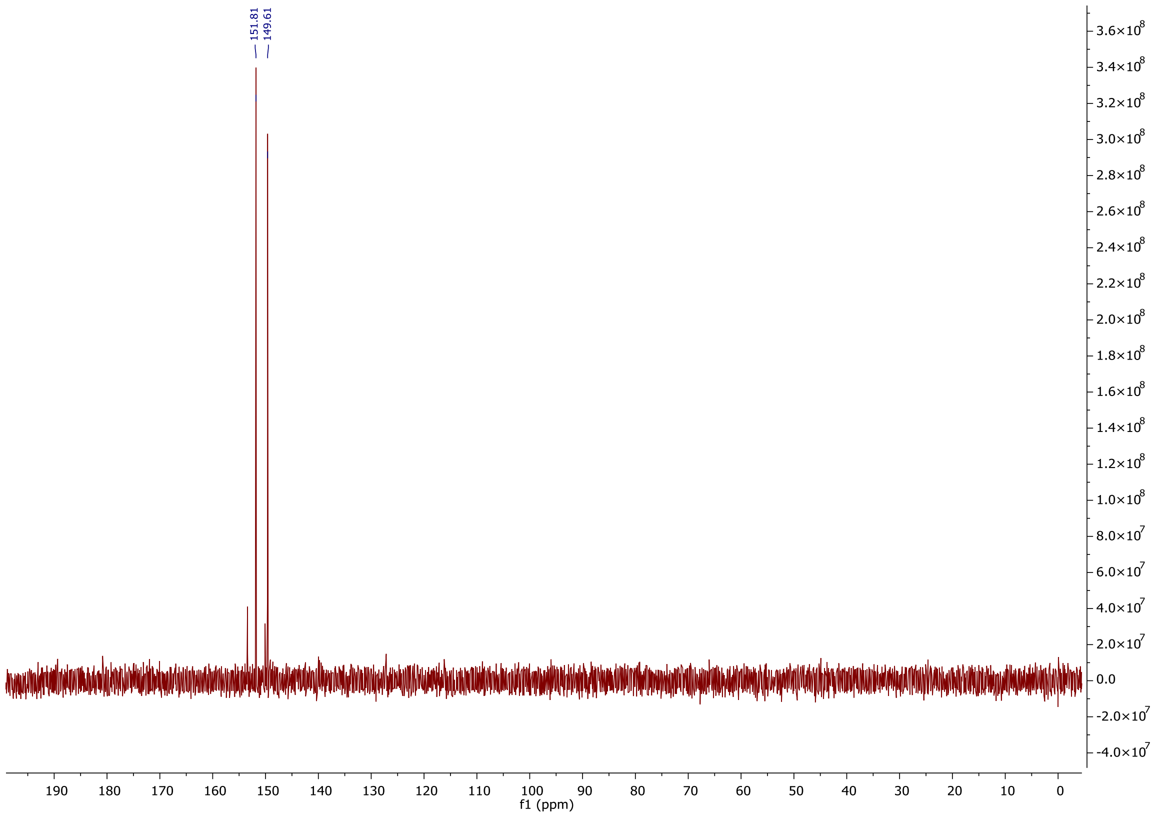

Compound ***MPC-3***

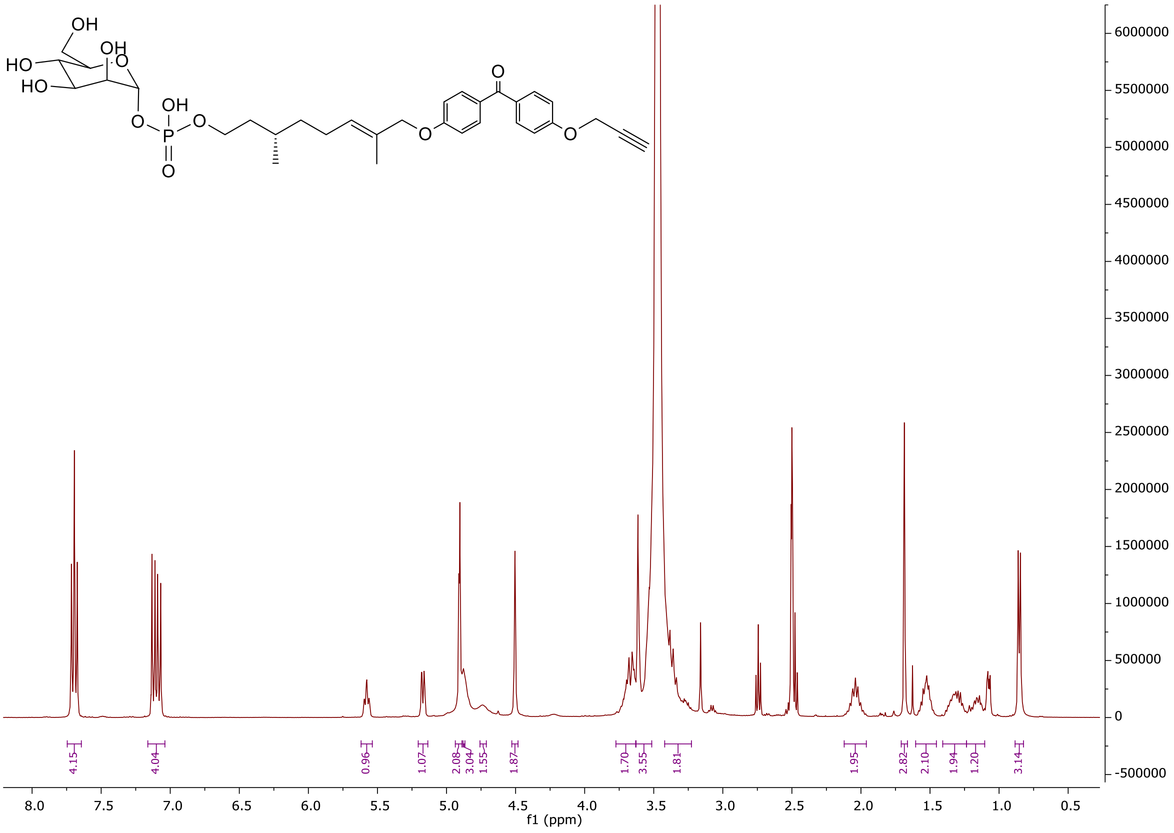

*Compound **MPC-3***

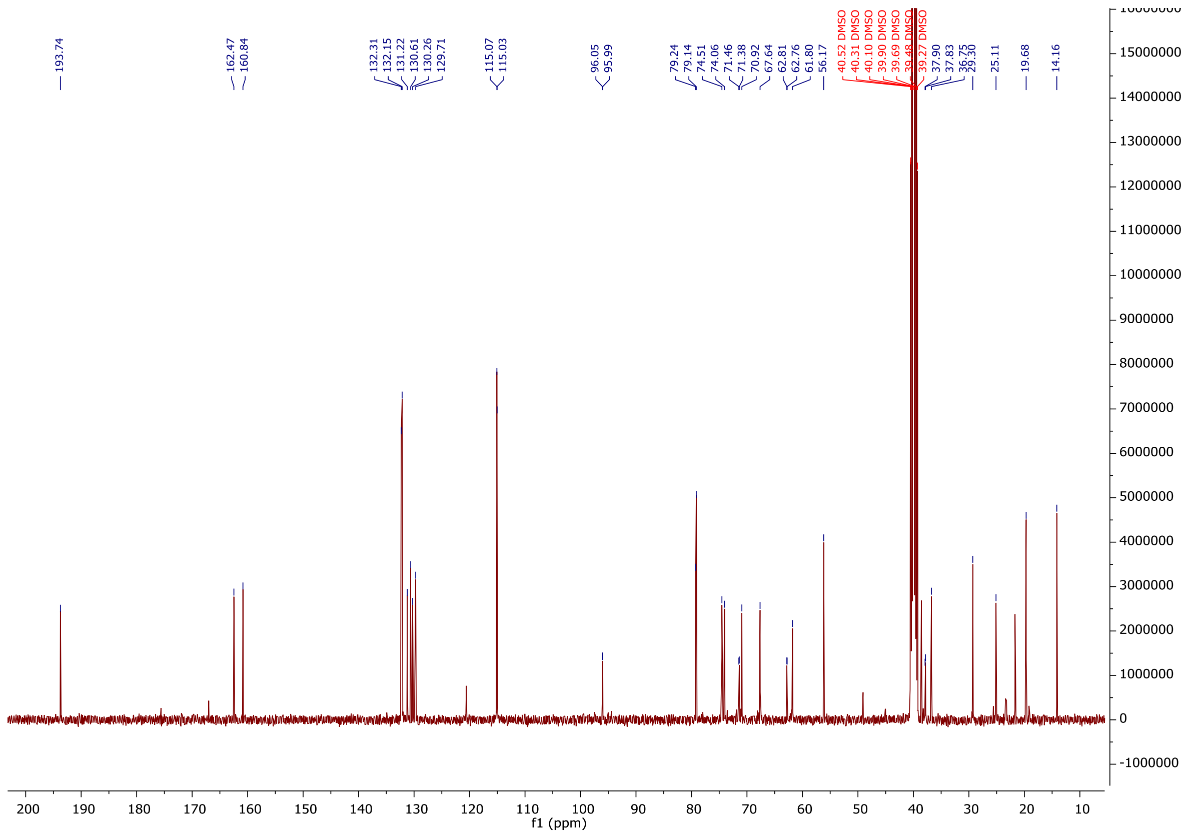

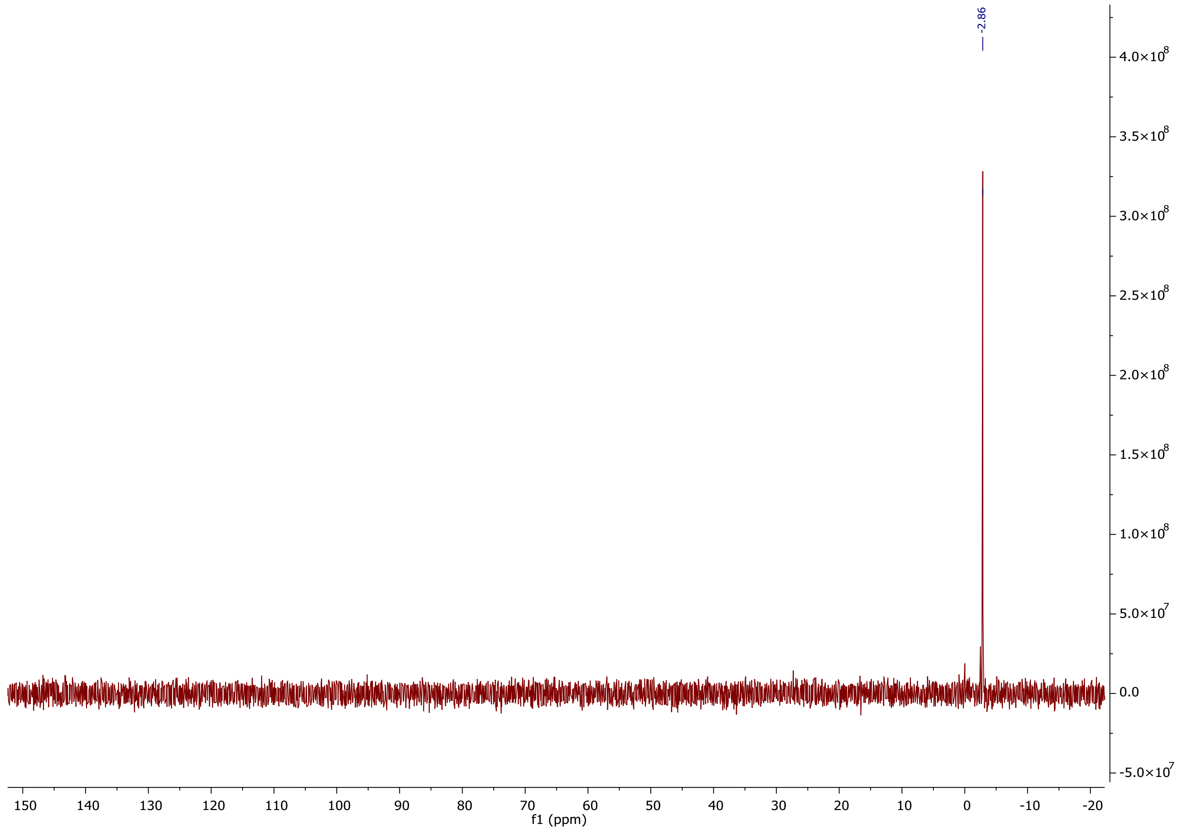

Compound ***MPC-3***

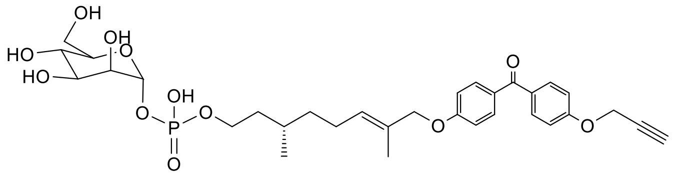

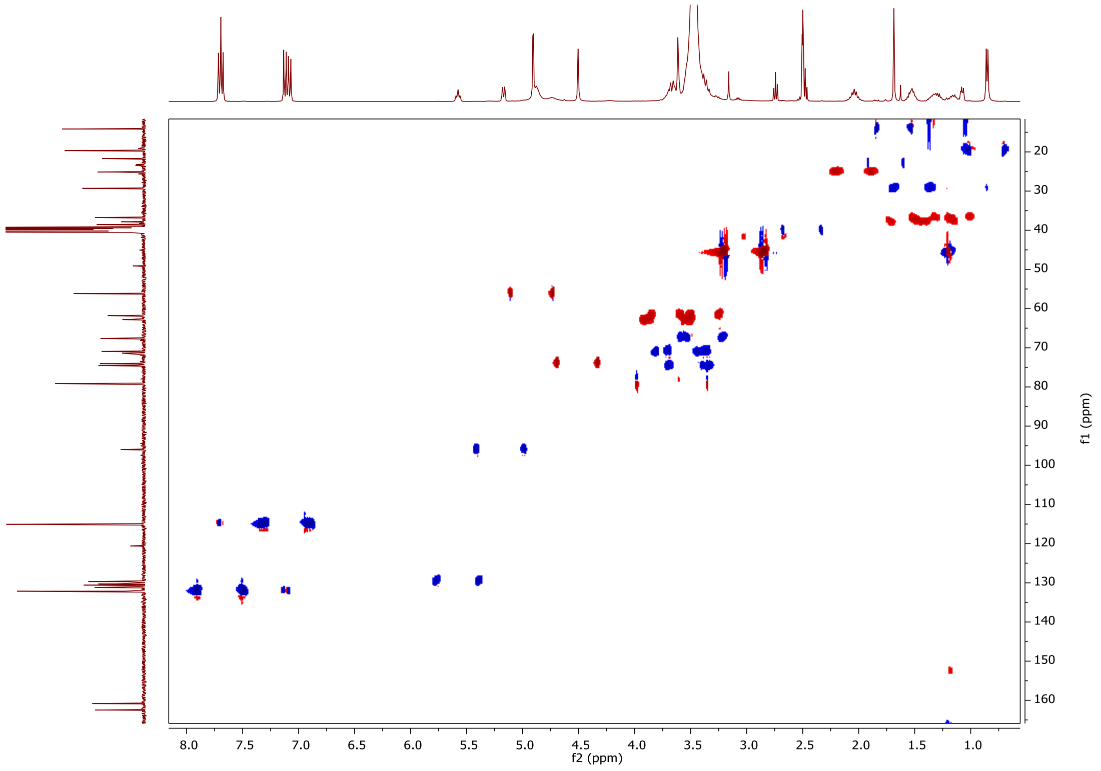

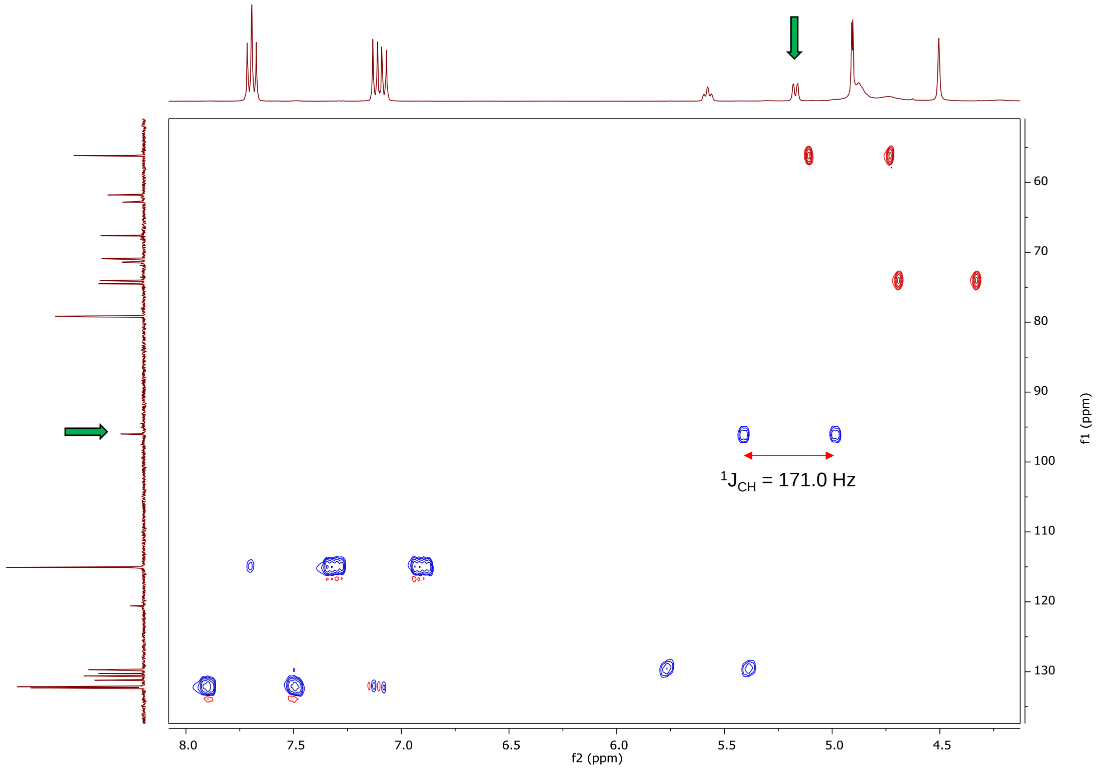

Analytical data RP-HPLC

Compound ***MPC-2***

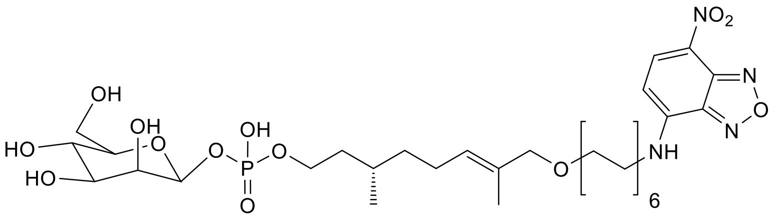

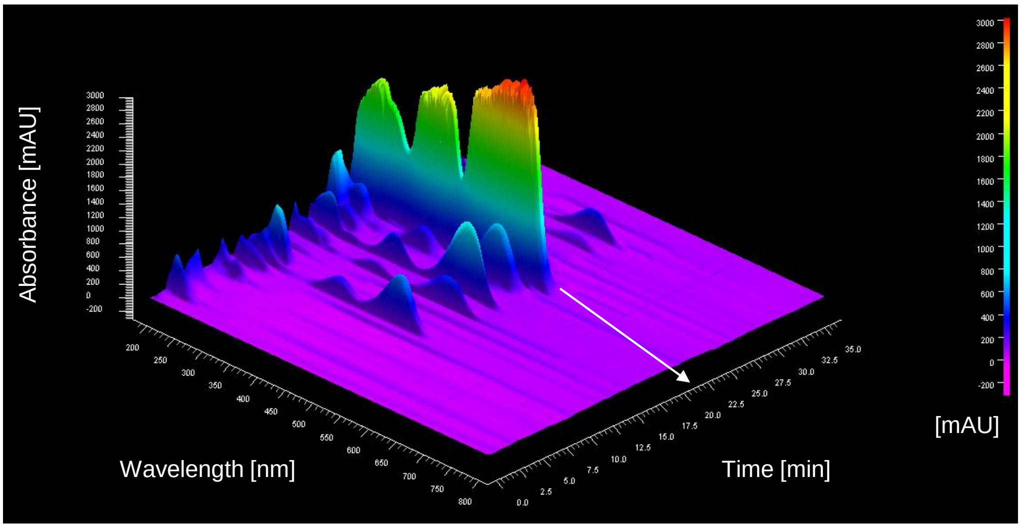

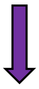

Main product at 21.04 min

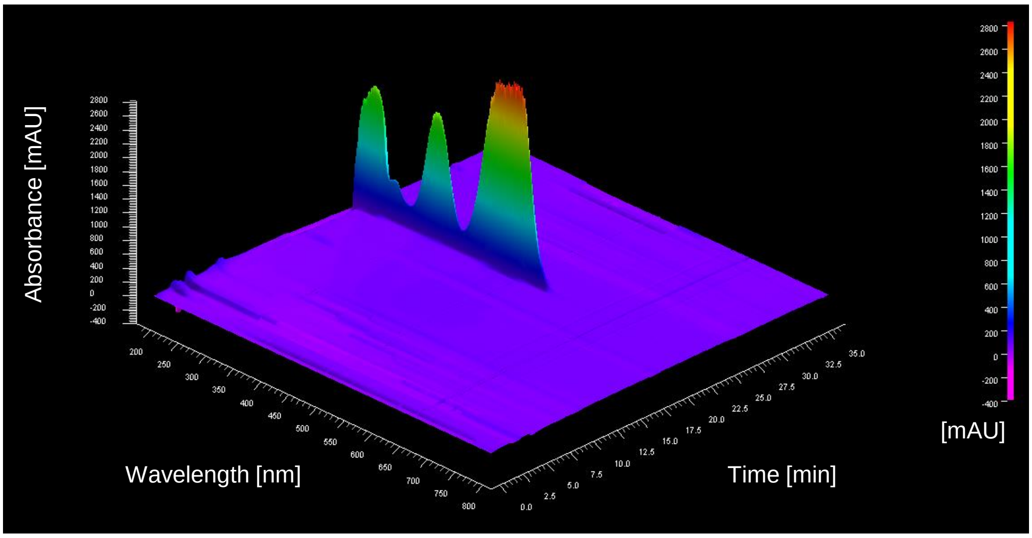

Data MS analysis

Compounds ***THP-Cit-BZP-yne***, ***Cit-BZP-yne*** *and* ***β-4Ac-Man-CEP***

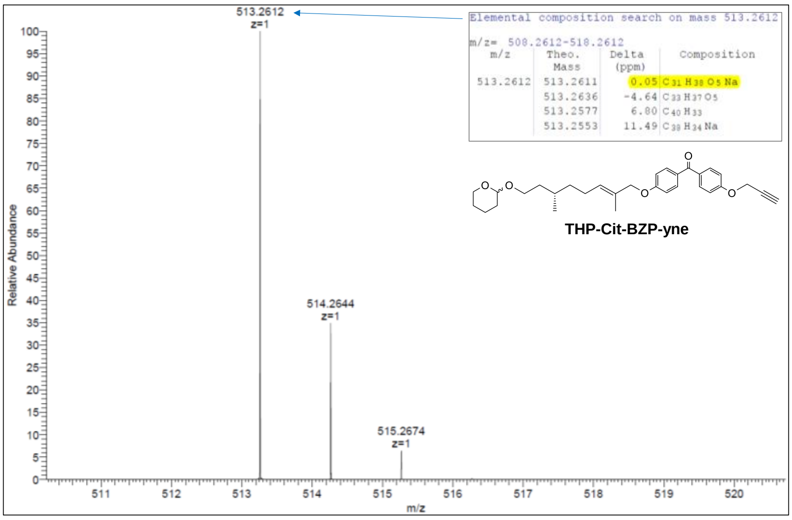

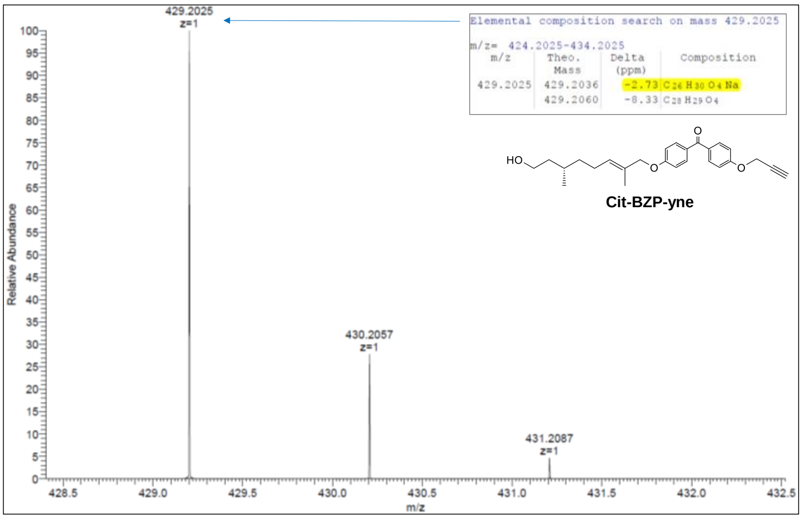

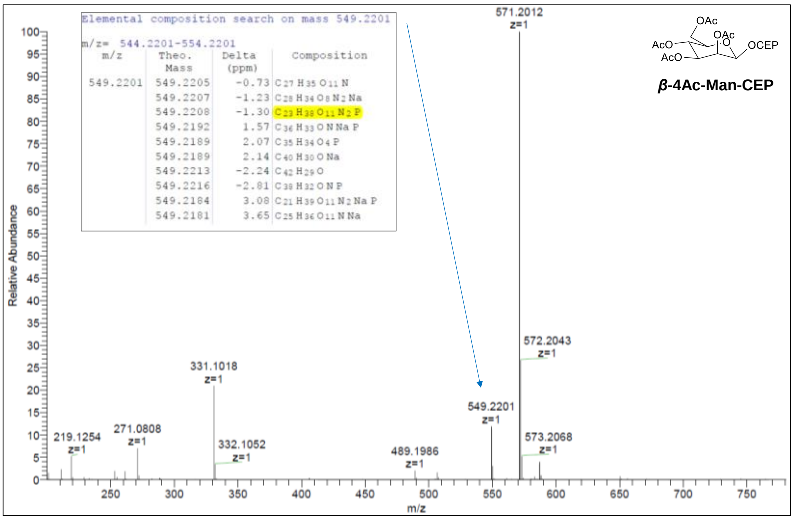

*Compounds **MPC-1**, **DMT-Cit** and **DMT-Cit-OH***

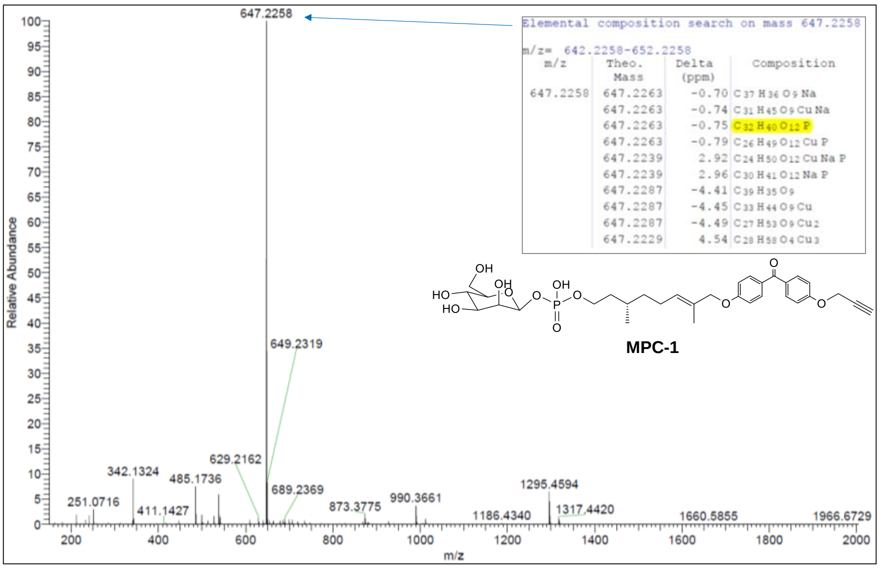

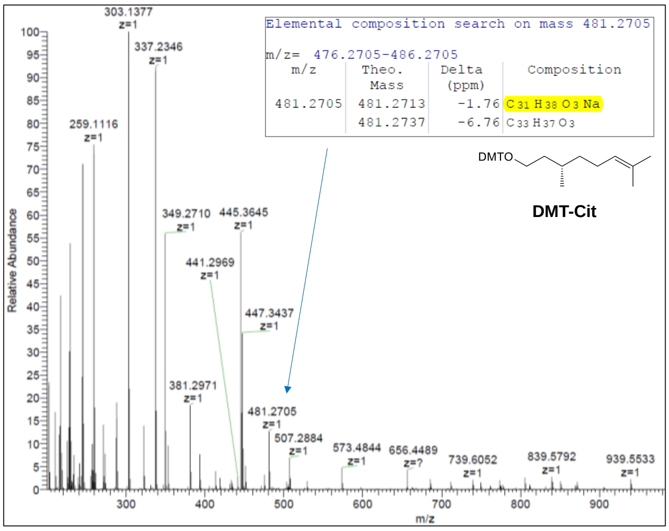

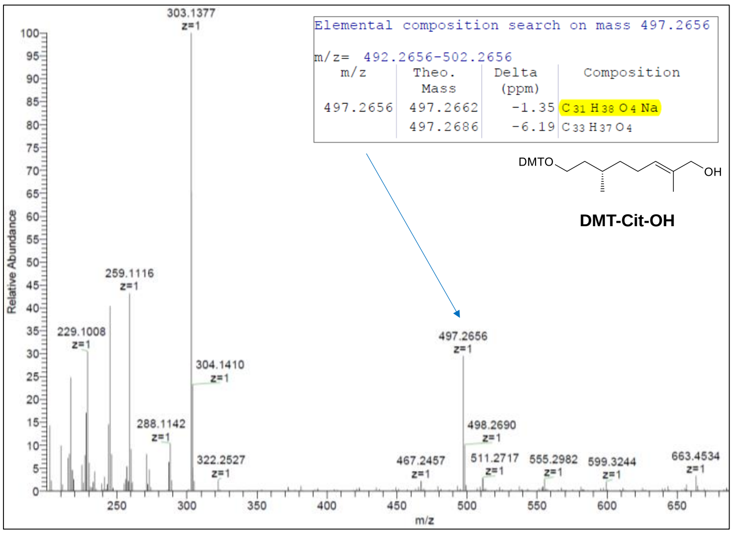

*Compounds **DMT-Cit-Dod-TBDMS**, **DMT-Cit-Dod** and **DMT-Cit-Dod-Pht***

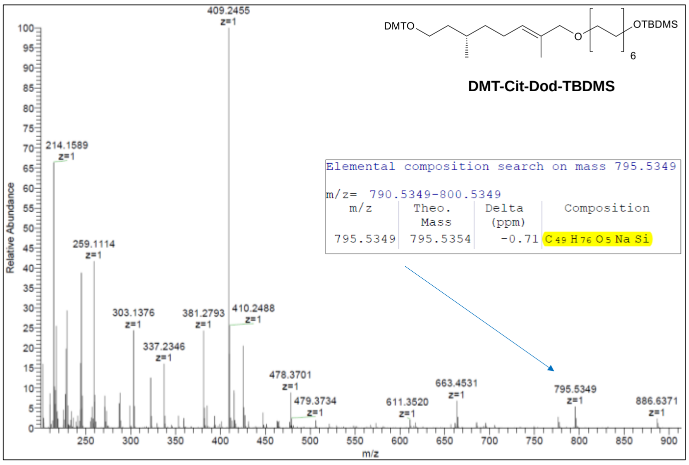

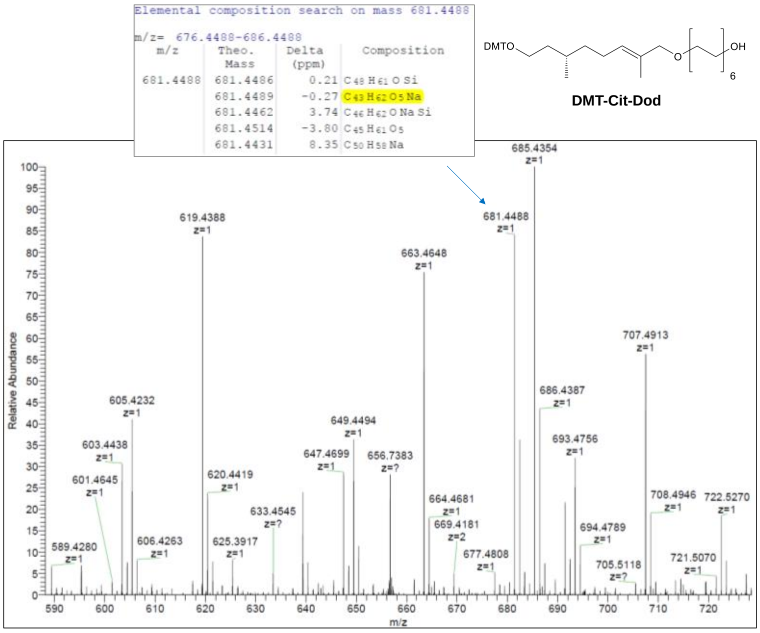

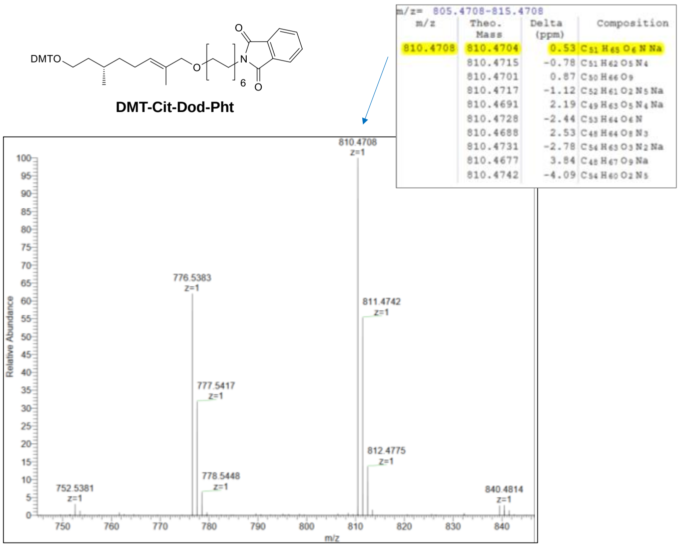

*Compounds **DMT-Cit-Dod-NH2**, **DMT-Cit-Dod-NBD** and **Cit-Dod-NBD***

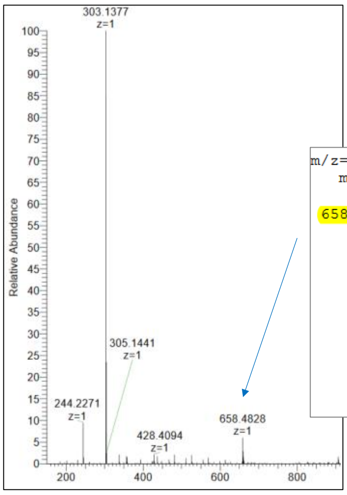

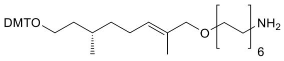

DMT-Cit-Dod-NH2

m/z= 653.4828-663.4828

m/z	Theo. Mass	Delta (ppm)	Composition
658.4828	658.4830	-0.28	$C_{43}H_{64}O_4N$
	658.4833	-0.70	$C_{44}H_{63}ON_2Na$
	658.4835	-1.05	$C_{29}H_{66}O_{10}N_6$
	658.4819	1.34	$C_{42}H_{61}N_5Na$
	658.4816	1.76	$C_{41}H_{62}O_3N_4$
	658.4843	-2.31	$C_{44}H_{60}N_5$
	658.4806	3.37	$C_{41}H_{65}O_4NNa$
	658.4851	-3.51	$C_{32}H_{67}O_8N_4Na$
	658.4803	3.79	$C_{40}H_{66}O_7$
	658.4857	-4.35	$C_{46}H_{62}ON_2$

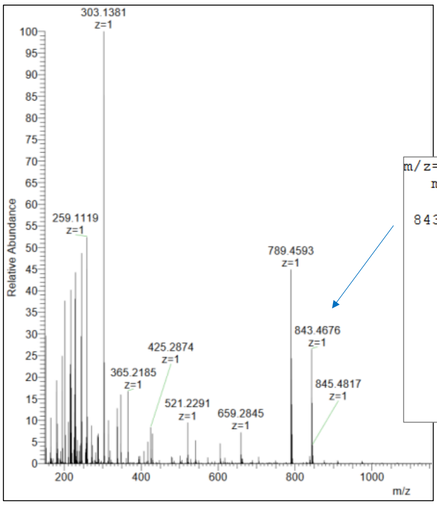

DMT-Cit-Dod-NBD

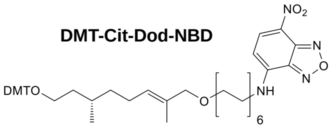

m/z= 838.4676-848.4676

m/z	Theo. Mass	Delta (ppm)	Composition
843.4676	843.4673	0.36	$C_{63}H_{59}N_2$
	843.4681	-0.57	$C_{51}H_{66}O_8NNa$
	843.4667	1.02	$C_{49}H_{64}O_7N_4Na$
	843.4664	1.34	$C_{48}H_{65}O_{10}N_3$
	843.4691	-1.83	$C_{51}H_{63}O_7N_4$
	843.4694	-2.16	$C_{52}H_{62}O_4N_5Na$
	843.4651	2.94	$C_{46}H_{63}O_9N_6$
	843.4649	3.21	$C_{61}H_{60}N_2Na$
	843.4705	-3.43	$C_{53}H_{65}O_8N$
	843.4646	3.54	$C_{60}H_{61}O_3N$

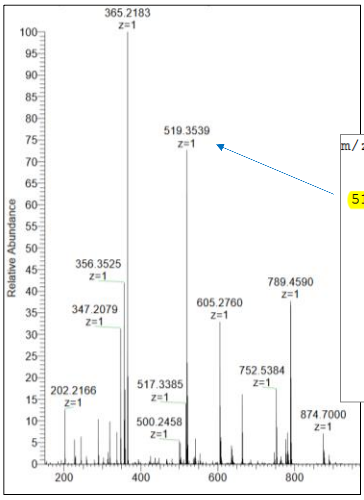

Cit-Dod-NBD

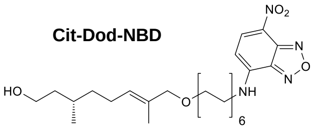

m/z= 514.3539-524.3539

m/z	Theo. Mass	Delta (ppm)	Composition
519.3539	519.3541	-0.40	$C_{28}H_{47}O_5N_4$
	519.3544	-0.93	$C_{29}H_{46}O_2N_5Na$
	519.3530	1.65	$C_{28}H_{50}O_6NNa$
	519.3528	2.18	$C_{27}H_{51}O_9$
	519.3554	-2.98	$C_{30}H_{49}O_6N$
	519.3557	-3.51	$C_{31}H_{48}O_3N_2Na$
	519.3517	4.23	$C_{26}H_{48}O_5N_4Na$
	519.3514	4.76	$C_{25}H_{49}O_8N_3$
	519.3568	-5.56	$C_{31}H_{45}O_2N_5$
	519.3504	6.81	$C_{25}H_{52}O_9Na$

*Compounds **MPC-2** and **MPC-3***

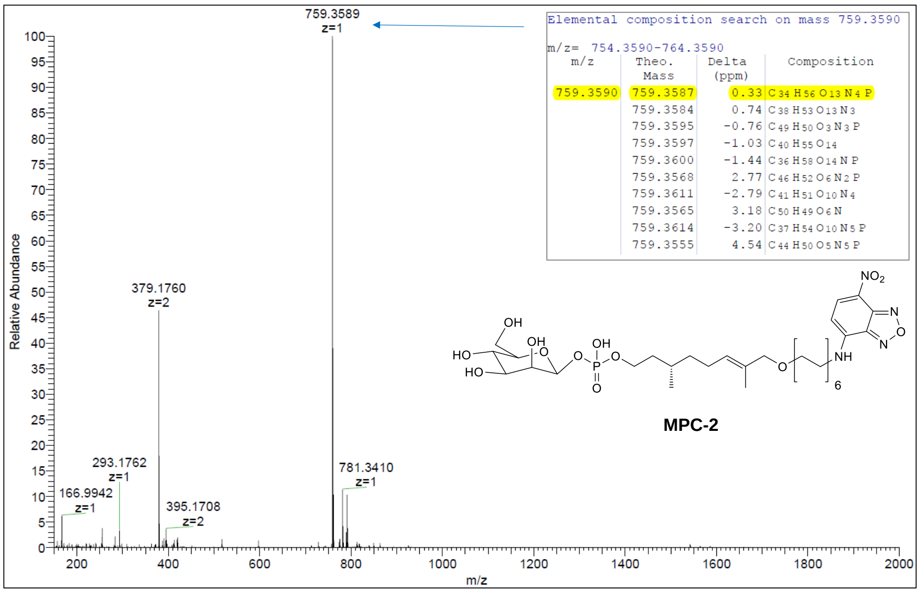

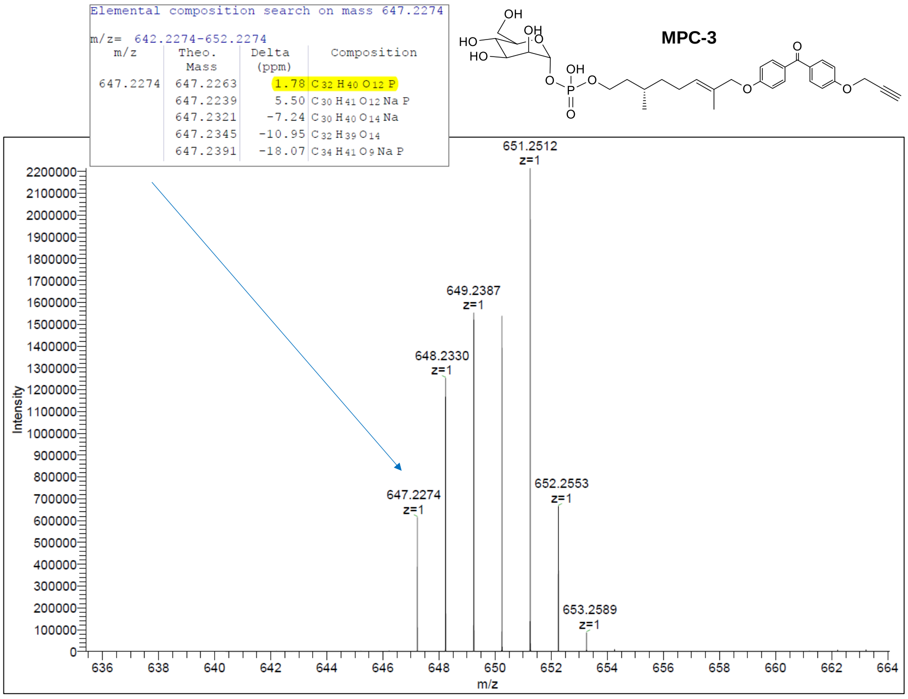

Supplement: File 1 — Complete descriptions of the syntheses, including the precursors THP-Cit-BZP-yne and DMT-Cit-Dod-NBD, additional information about α-4Ac-Man, and the analytical data: NMR and HRMS spectra. [file Beilstein_J_Org_Chem-16-1732-s001.pdf]
